# Cytoskeleton-associated protein 4 affects podocyte cytoskeleton dynamics in diabetic kidney disease

**DOI:** 10.1172/jci.insight.181298

**Published:** 2025-06-10

**Authors:** Roberto Boi, Emelie Lassén, Alva Johansson, Peidi Liu, Aditi Chaudhari, Ramesh Tati, Janina Müller-Deile, Mario Schiffer, Kerstin Ebefors, Jenny Nyström

**Affiliations:** 1 Institute of Neuroscience and Physiology, Sahlgrenska Academy, Gothenburg University, Gothenburg, Sweden.; 2 Bioscience Renal, Research and Early Development, Cardiovascular, Renal and Metabolism, BioPharmaceuticals R&D, AstraZeneca, Gothenburg, Sweden.; 3 Department of Nephrology, Friedrich-Alexander-Universität Erlangen-Nürnberg, Erlangen, Germany.; 4 Mount Desert Island Biological Laboratory, Salisbury Cove, Maine, USA.

**Keywords:** Cell biology, Nephrology, Chronic kidney disease, Cytoskeleton, Integrins

## Abstract

Podocytes are kidney glomerular cells that depend on rigorously regulated cytoskeleton components and integrins to form and maintain the so-called foot processes, apparatuses that attach podocytes to the glomerular basement membrane and connect them to neighboring podocytes. In diabetic kidney disease (DKD) these foot processes are effaced as a result of cytoskeleton dysregulation, a phenomenon that gradually reduces glomerular filtration. Cytoskeleton-associated protein 4 (CKAP4) is a known linker between the endoplasmic reticulum, integrins, and microtubular cytoskeleton. Since CKAP4 gene expression is downregulated in glomeruli from patients with DKD but not in other chronic kidney diseases, we hypothesized a role for CKAP4 in the mechanisms leading to foot process effacement (FPE) in DKD. CKAP4 mRNA reduction in podocytes in DKD was demonstrated in human kidney biopsies. Knockdown of CKAP4 in vivo in zebrafish resulted in edema, proteinuria, and foot process effacement, all typical features of DKD. Knockdown of CKAP4 in vitro led to disruption of the actin cytoskeleton and of the microtubular orientation. Moreover, it caused a downregulation of several integrins. These findings indicate that CKAP4 is crucial for foot process dynamics of podocytes. Its reduction, unique to DKD, is mechanistically connected to the pathophysiological processes leading to podocyte FPE.

## Abstract 2

CKAP4 is reduced in the glomeruli of diabetic kidney disease patients. We demonstrated that CKAP4 reduction causes dysregulation of the cytoskeleton and leads to proteinuria.

## Graphical abstract

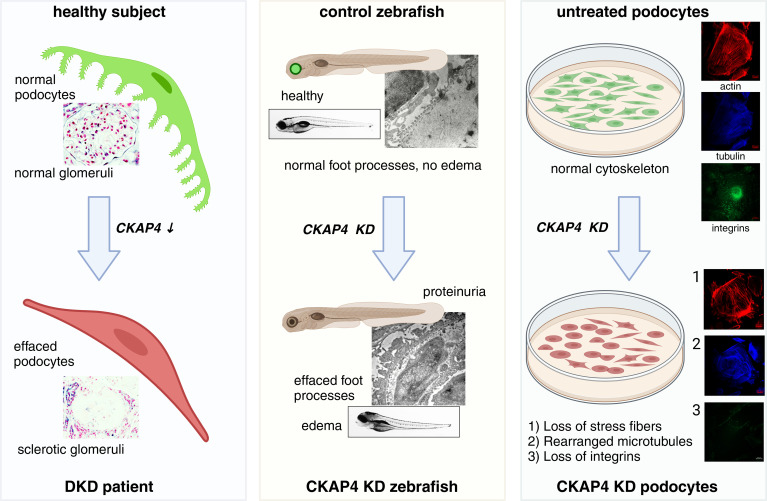

## Introduction

It is estimated that around 10%–40% of patients with diabetes will develop diabetic kidney disease (DKD) (1). DKD is characterized by several glomerular alterations: mesangial expansion, podocyte foot process effacement (FPE), glomerular basement membrane thickening, and loss of endothelial fenestrations (2). In particular, pathological alterations of the podocyte actin cytoskeleton and loss or dysfunction of several proteins in the podocyte slit diaphragm, the cell-to-cell junction structure responsible for blood filtration, have been implicated as causes of FPE (3, 4).

The cytoskeleton is composed of 3 distinct structures: actin microfilaments (stress fibers), intermediate filaments, and microtubules. In podocytes, the actin microfilaments extend to the primary and secondary foot processes and are directly connected to the slit diaphragm, while intermediate filaments and microtubules are found mainly in the cell body and are necessary for primary foot process formation (5–7). The 3 cytoskeleton components are connected at the end of adjacent major foot processes (7–9). Microtubules are hollow cylindrical structures made of α- and β-tubulin and have a polar orientation. The minus-end is found in the center of the cell and the plus-end in the periphery. Podocytes are characterized by a nonuniform microtubular polarity in that the minus-ends are also found in the cell periphery. This uniqueness is necessary for foot process formation (5). In the cell body, the microtubular network is interconnected to the endoplasmic reticulum (ER).

All 3 cytoskeletal components interact; hence, the disruption of one affects the others. Moreover, changes that take place at the podocyte slit diaphragm can be transmitted to the nuclei via crosstalk between actin microfilaments and microtubules. This enables podocytes to respond to and send signals from and to the foot processes (10). Since microtubules are important for foot process formation, alterations of the microtubules in the cell body might be transmitted to the peripheral actin microfilaments and cause FPE.

Given the importance of cytoskeleton architecture in podocytes, proteins that are able to interact with or modulate cytoskeletal components may have a role in the development of chronic kidney disease (CKD). One of the proteins involved in anchoring microtubules to the ER is the cytoskeleton-associated protein 4 (CKAP4) (11).

CKAP4 is a nonglycosylated, 63 kDa, type II integral protein that was first discovered in the ER (12, 13), where it stabilizes and facilitates the folding of ER sheets (14, 15). CKAP4 contains a 106–amino acid–long domain with ER-anchoring and microtubule-binding functionality (11). Overexpression of CKAP4 has been shown to increase the number of ER sheets in epithelial cells and to induce rearrangement in both ER and the microtubular network (14, 16, 17). Moreover, CKAP4 knockdown has been shown to cause reduction of actin polymerization and increased cell motility (18). Although CKAP4’s role in disease is often associated with cancer (with conflicting reports on whether it should be considered a cancer suppressor or a cancer promoter molecule) (19), CKAP4 has recently been connected to vascular calcification in CKD. Serum levels of CKAP4 were found to be elevated in patients with CKD of different etiology when compared with healthy patients with normal renal function, and CKAP4 was found to cause nuclear translocation of Yes1 associated transcriptional regulator (YAP) (20). Cytoplasmic YAP regulates cell transduction of mechanical stimuli by interacting with actin and integrins (21, 22), again connecting CKAP4 with cytoskeleton dynamics.

Since both depletion and overexpression of CKAP4 have been shown to affect the microtubular network, and as CKAP4 is also involved in actin regulation, we hypothesized that CKAP4 is essential for maintaining a functional cytoskeleton in podocytes and that loss of CKAP4 contributes to the cytoskeletal dysfunction and podocyte FPE observed in CKD.

## Results

### Patients with DKD have decreased glomerular CKAP4 expression.

**Figure 1:**
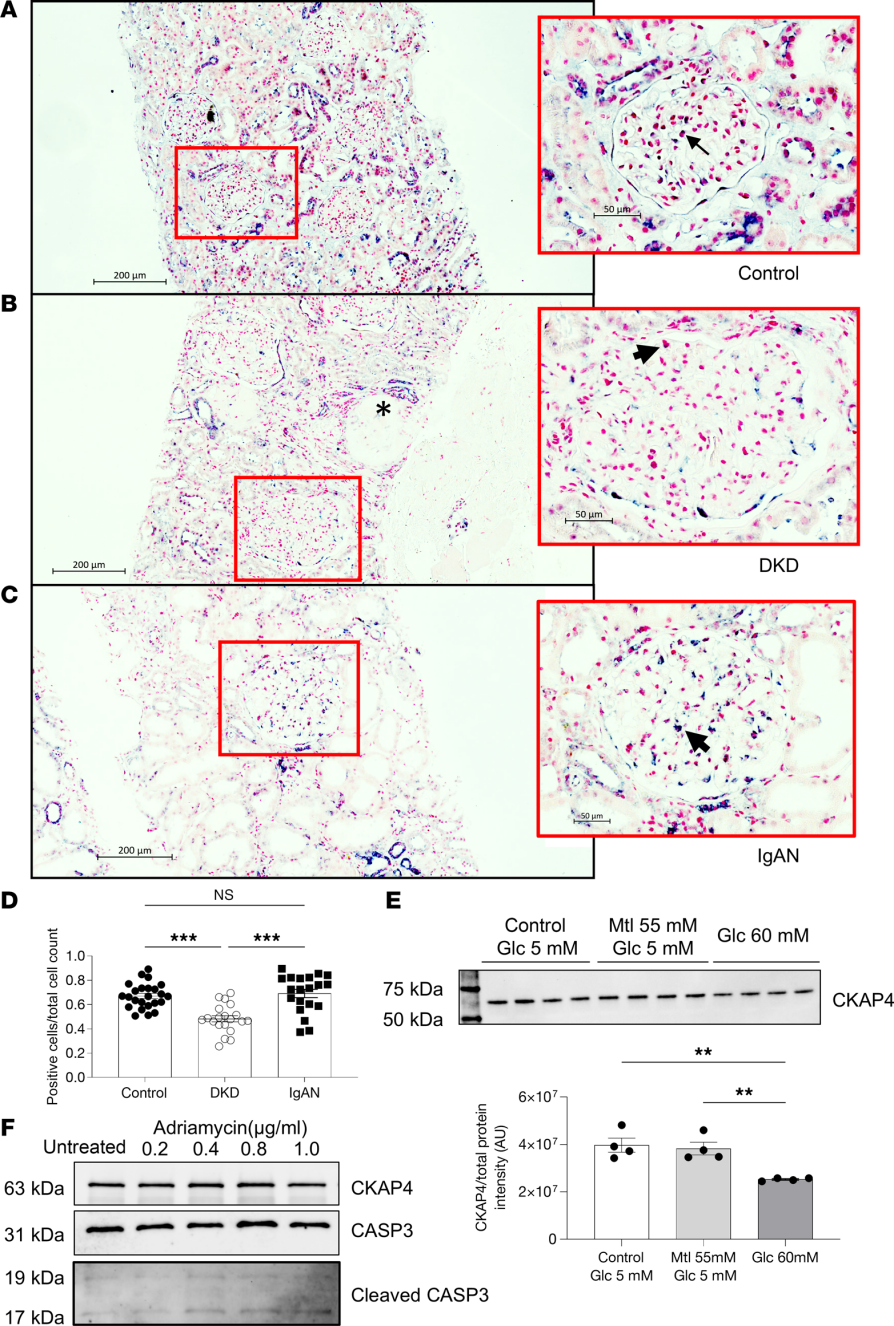
CKAP4 is downregulated in glomeruli in patients with DKD. CKAP4 mRNA was detected using in situ hybridization in control (**A**), DKD patient biopsies (**B**), and IgAN patient biopsies (**C**). A purple/dark blue staining in the nuclear region characterizes cells positive for CKAP4 expression. Negative cells are characterized by a pink/red nuclear staining. The ratio of positive cells to total glomerular cells was used to quantify the extent of CKAP4 gene expression reduction in DKD (**D**). Completely differentiated human podocytes (HPODs) treated for 2 weeks with 60 mM glucose showed a 20%–30% reduction of CKAP4 (**E**). Treatment of HPODs with adriamycin for 24 hours does not cause a decrease in CKAP4 at protein level, although cleaved CASP3 level is increased in treated cells, indicating apoptosis (**F**). Unedited/uncropped total protein blots used for normalization calculation are provided as supplemental materials. Error bars represent average ± SEM. **P < 0.01, ***P < 0.001. **D**: 1-way ANOVA with Tukey’s multiple comparisons test, *n* = 25 (controls), 20 (DKD), 22 (IgAN) glomeruli. We scored 5 biopsies from 5 patients per group (4 for IgAN), and 5 (for controls, IgAN) or 4 (for DKD) glomeruli per biopsy. **E**: *n* = 4 replicates, 1-way ANOVA with multiple comparisons. The asterisk in **B** indicates a sclerotic glomerulus. The arrows in the respective zoomed-in sections of **A**–**C** indicate cells showing positive staining for CKAP4. CASP3, caspase-3; CKAP4, cytoskeleton associated protein 4; DKD, diabetic kidney disease; Glc, glucose; IgAN, immunoglobulin A nephropathy, Mtl, mannitol.

CKAP4 expression was investigated in previously published CKD glomerular transcriptomics datasets. CKAP4 expression was significantly lower in patients with DKD compared with healthy individuals. There was no significant difference in other CKD etiologies present in the datasets (Table 1) (23, 24). To validate this, we performed CKAP4 mRNA in situ hybridization on biopsies from 5 patients with DKD, 4 patients with immunoglobulin A nephropathy (IgAN), and 5 controls. The most pronounced expression of CKAP4 mRNA was detected in podocytes. Glomerular cells were scored for presence (purple/dark blue color) or absence (only pink/red nuclear staining) of CKAP4 mRNA (DKD: 20 glomeruli, IgAN: 22 glomeruli, control: 25 glomeruli). Sclerotic glomeruli showed absent or low CKAP4 expression and were excluded. The ratio between positive cells and total cells was used for statistical analysis. While the IgAN biopsies showed CKAP4 levels similar to the controls, glomeruli from DKD presented a significantly (*P* < 0.001) lower ratio of CKAP4-positive cells (Figure 1, A–D). This was in concordance with the transcriptomics data reported in Table 1. To establish a direct connection between DKD, CKAP4, and podocytes, we treated human podocytes (HPODs) with 60 mM glucose for 2 weeks. Hyperglycemia caused a reduction of CKAP4 of around 20%–30% when compared with its osmotic control and untreated cells (Figure 1E).

Moreover, HPODs were treated with different concentrations of adriamycin (ADR) for 24 hours. ADR is typically used as an injury model for focal segmental glomerulosclerosis. HPOD apoptosis was confirmed by measuring cleaved versus total CASP3. The level of cleaved CASP3 increased proportionally to the ADR concentration used for treatment, while total CASP3 level was stable. CKAP4 was not regulated by the treatment with adriamycin (Figure 1F).

Taken together, these results demonstrate that patients with DKD have a decreased gene expression of CKAP4 and establish a direct connection between hyperglycemia and reduction of CKAP4 expression.

### CKAP4 is expressed in glomerular cells in vitro and in vivo.

**Figure 2:**
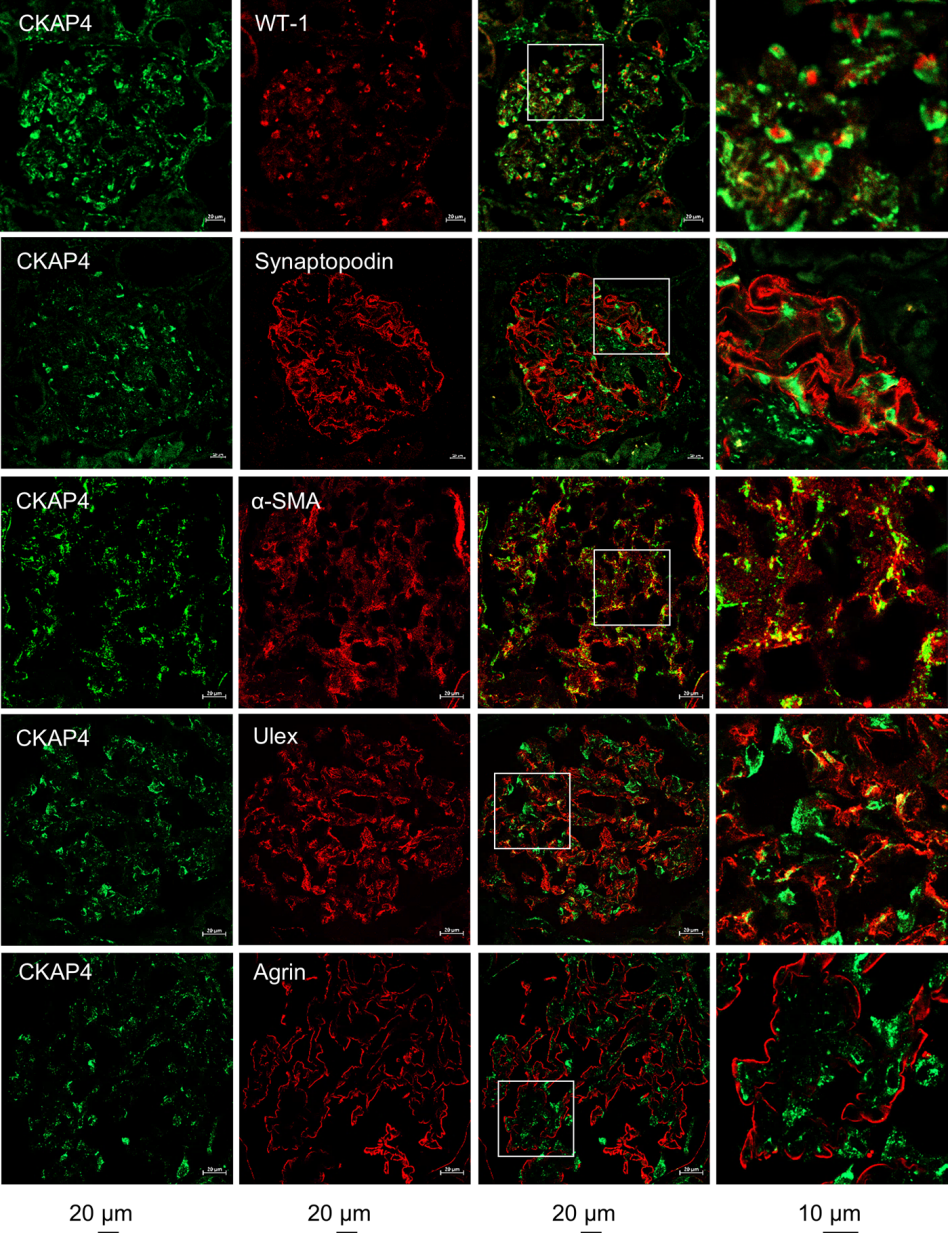
CKAP4 is expressed in all glomerular cell types in human glomeruli. Immunofluorescence staining of human kidney tissue for CKAP4 (green) and various markers for glomerular cells (red) to illustrate CKAP4 localization in the glomerulus. The first column shows CKAP4 staining. The second column shows, respectively: WT-1, marker for podocyte nuclei; synaptopodin, marker for podocyte foot processes; α-SMA, marker for mesangial cells; *Ulex Europaeus* Agglutinin I, marker for endothelial cell; agrin, marker for basement membrane. The third column shows colocalization; the fourth column contains zoomed area (white squares in the third column). Representative scale bars are placed below each column. All zoomed-in areas in the last column had an area of 65 μm^2^. CKAP4, cytoskeleton associated protein 4; WT-1, Wilms tumor 1; α-SMA, smooth muscle actin.

Expression of CKAP4 in human kidney biopsies and specifically in the glomerulus was determined by immunofluorescence (Figure 2). Costaining of CKAP4 with WT-1 showed expression of CKAP4 in the cell body of podocytes, while costaining with anti-synaptopodin (podocyte foot processes marker) showed no colocalization. Partial colocalization of CKAP4 with α-SMA (mesangial cell marker) and with the endothelial cell–specific lectin *Ulex Europaeus* Agglutinin I was observed. There was no colocalization with the glomerular basement membrane proteoglycan agrin.

**Figure 3:**
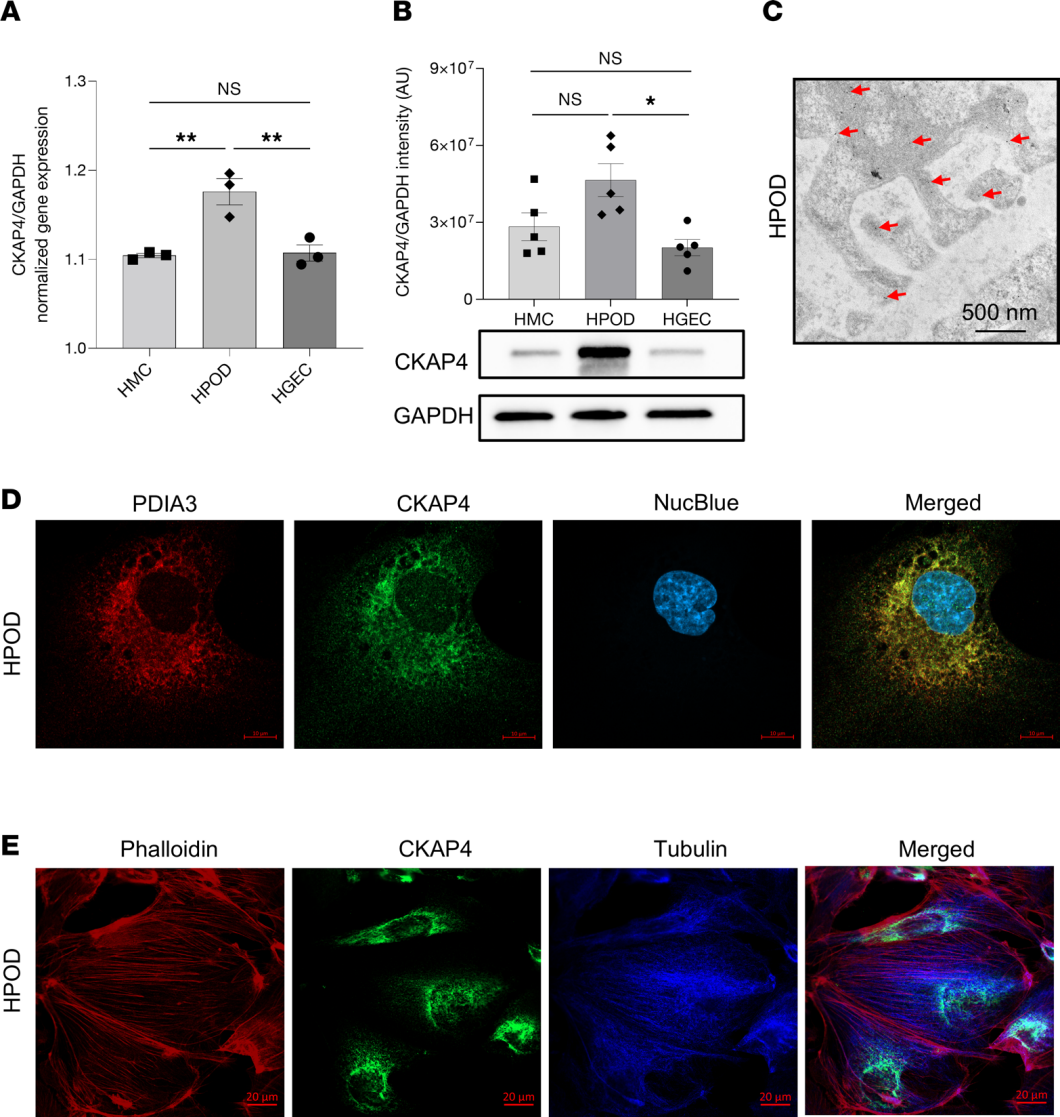
CKAP4 expression and localization in glomerular cells in vitro. CKAP4 gene expression was quantified with qPCR (**A**) and protein expression by Western blot (**B**) in cultured HGECs, HMCs, and HPODs, normalized against the respective GAPDH gene/protein levels. Immunogold electron micrograph of podocyte feet and major processes; red arrows point at gold particles (**C**). Immunofluorescence of PDIA3 (ER marker, red), CKAP4 (green), and DAPI (blue) showing expression of CKAP4 in the ER of HPODs. Scale bar, 10 μm. (**D**). Immunofluorescence of phalloidin (actin cytoskeleton, red), CKAP4 (green), and tubulin (blue) showing the localization of CKAP4 in relation to cytoskeleton components (**E**). **A**: 1-way ANOVA with multiple comparisons, *n* = 3 per cell type. **B**: Kruskal-Wallis plus Dunn’s post hoc test, *n* = 5 per cell type. One representative blot is shown. Error bars in both panels represent average ± SEM. *P < 0.05, **P < 0.01. AU, arbitrary units; CKAP4, cytoskeleton associated protein 4; HMCs, human mesangial cells; HPODs, human podocytes; HGECs, human glomerular endothelial cells; PDIA3, protein disulfide-isomerase A3.

Next, the expression of CKAP4 in vitro was examined in the glomerular cell types. Western blot and quantitative PCR (qPCR) analysis of CKAP4 expression in human mesangial cells (HMCs), human glomerular endothelial cells (HGECs), and HPODs showed its presence in all 3 cell types, with the highest expression at gene (HPODs vs. HGECs, *P* < 0.01; HPODs vs. HMCs, *P* < 0.01) and protein levels (HPODs vs. HGECs, *P* < 0.05) in podocytes (Figure 3, A and B).

Immunogold labeling of CKAP4 in human kidney sections also verified CKAP4 presence in podocytes (Figure 3C). Immunofluorescence of cultured HPODs showed immunofluorescence colocalization of CKAP4 with ER marker PDIA3 (Figure 3D), in agreement with previous findings in other tissues (14, 16). The respective positions and overlaps between CKAP4 and HPOD cytoskeleton components (actin and tubulin) are shown in Figure 3E.

### Knockdown of the CKAP4 homolog in zebrafish induces proteinuria.

**Figure 4:**
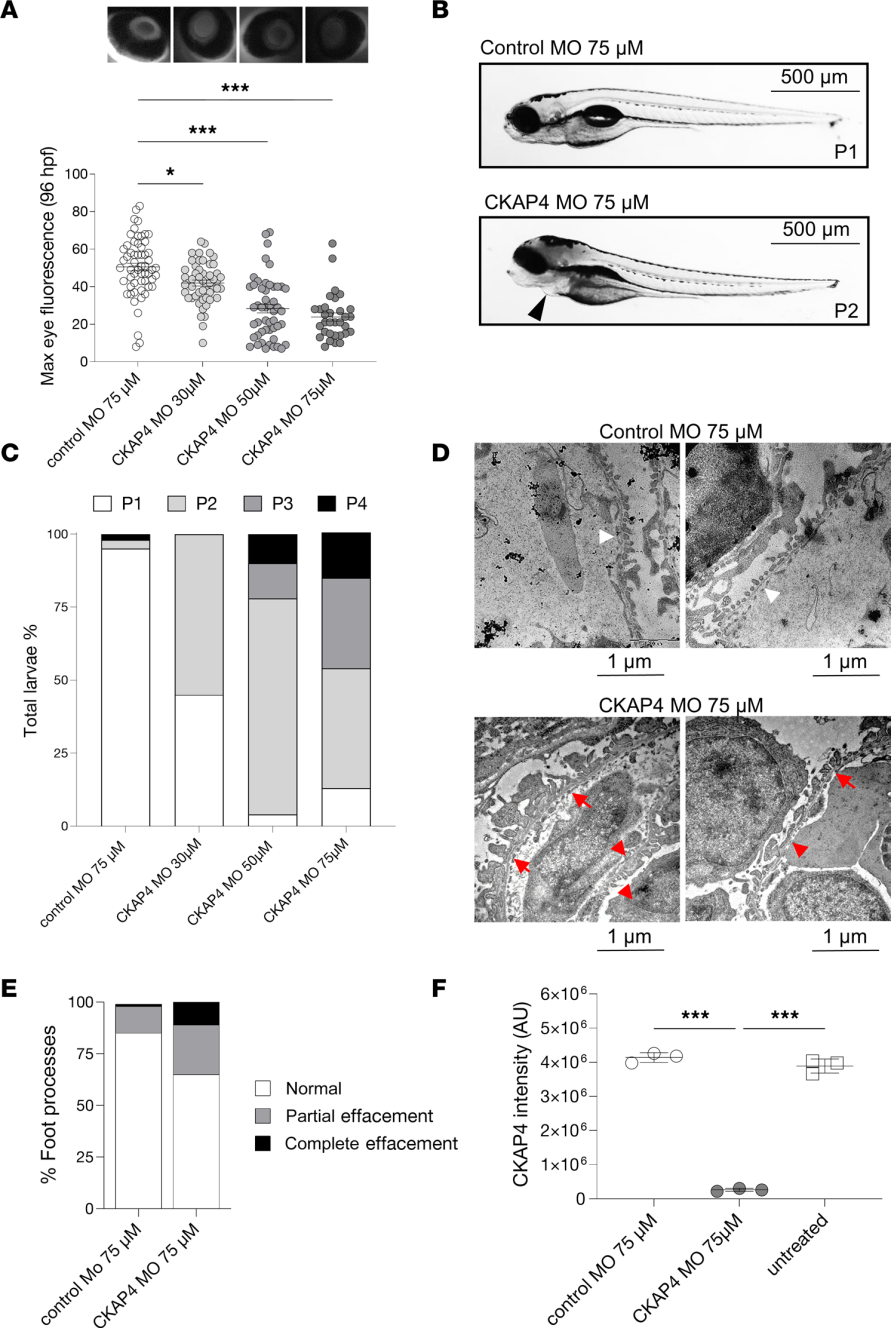
CKAP4 zebrafish homolog knockdown causes proteinuria and podocyte FPE. CKAP4 was knocked down in zebrafish using MO in different concentrations, 30, 50, and 75 μM MO. Proteinuria was measured by reduction of eye fluorescence (**A**). Example of normal phenotype (P1, no edema) and fish with mild edema (P2) in CKAP4 MO; the arrow points at pericardial edema (**B**). Assessment of fish phenotypes in the different groups; from P1 to P4 (severe edema) was conducted with reference to Hanke et al. (25, 27) and Ursu et al. (26) (**C**). Representative electron microscopy pictures of the filtration barrier of zebrafish glomeruli from control MO and CKAP4 MO (75 μM). Control MO shows normal podocyte foot processes (this pattern is indicated with short white arrows) while the CKAP4 MO shows podocyte FPE (partial effacement patterns are indicated with red arrows, complete effacement with short red arrows) (**D**). Quantification of podocyte FPE percentages in control MO and CKAP4 MO shows increased effacement in CKAP4 MO–treated zebrafish (**E**). Validation of CKAP4 MO knockdown of CKAP4 as obtained via proteomics (**F**). **A**: a minimum of 32 larvae per group was used. Error bars: average ± SEM. *P < 0.05, ***P < 0.001, Mann-Whitney test. **E**: *n* = 336 (control MO), *n* = 319 (CKAP4 MO) foot processes were counted, from *n* = 14 (control MO) and *n* = 8 (CKAP4 MO) independent images per group. **F**: 3 independent experiments were performed; each treatment in each replicate is derived from lysate of a minimum of 8 pooled embryos. Error bars in both panels represent average ± SEM. ***P < 0.001, 1-way ANOVA with Tukey’s multiple-comparison test. CKAP4, cytoskeleton associated protein 4; MO, morpholino.

To assess whether reduced CKAP4 expression affects the filtration barrier function, the zebrafish homolog of CKAP4 was knocked down in a zebrafish model using a morpholino (MO) blocking mRNA translation. CKAP4 MO induced proteinuria in a dose-dependent manner (30 μM: *P* < 0.05, 50 and 75 μM: *P* < 0.001, compared with control MO) (Figure 4A). CKAP4 MO zebrafish developed edema. Edematous phenotypes were assessed and quantified as described previously (from P1, no edema, to P4, severe edema) (Figure 4, B and C) (25–27).

Glomerular morphology was investigated using transmission electron microscopy (TEM). The CKAP4 MO group presented with podocyte FPE, whereas zebrafish injected with control MO showed normal podocyte morphology (Figure 4D). Quantification of foot processes in control MO– and CKAP4 MO–treated zebrafish was performed as previously described (28) and showed an increase of partially effaced (from 13% to 24%) and completely effaced (from 1% to 11%) foot processes in the CKAP4 MO group (Figure 4E). CKAP4 MO knockdown (KD) was confirmed with mass spectrometry (Figure 4F and Supplemental Table 1; supplemental material available online with this article; https://doi.org/10.1172/jci.insight.181298DS1). Downregulated CKAP4 (–90%) was detected in both the CKAP4 MO versus control MO and CKAP4 MO versus untreated comparisons (in both cases *P* < 0.001).

### Knockdown of CKAP4 in podocytes alters the morphology of actin filaments, microtubules, and the ER.

Since CKAP4 is known to anchor the microtubular cytoskeleton component to the ER, loss of actin cytoskeleton (stress fiber structures) is associated with podocyte FPE, and both structural components are connected at the major foot process level in podocytes, we hypothesized that a decreased expression of CKAP4 would affect both microtubules and actin cytoskeleton. To verify this, we examined the morphology of ER, microtubules (tubulin), and actin microfilaments (stress fibers) in CKAP4 KD HPODs.

Following lentiviral knockdown, CKAP4 gene expression was reduced by over 90% (Supplemental Figure 1A), while the protein level was only halved, likely due to a slow protein turnover time, as observed earlier (15).

**Figure 5:**
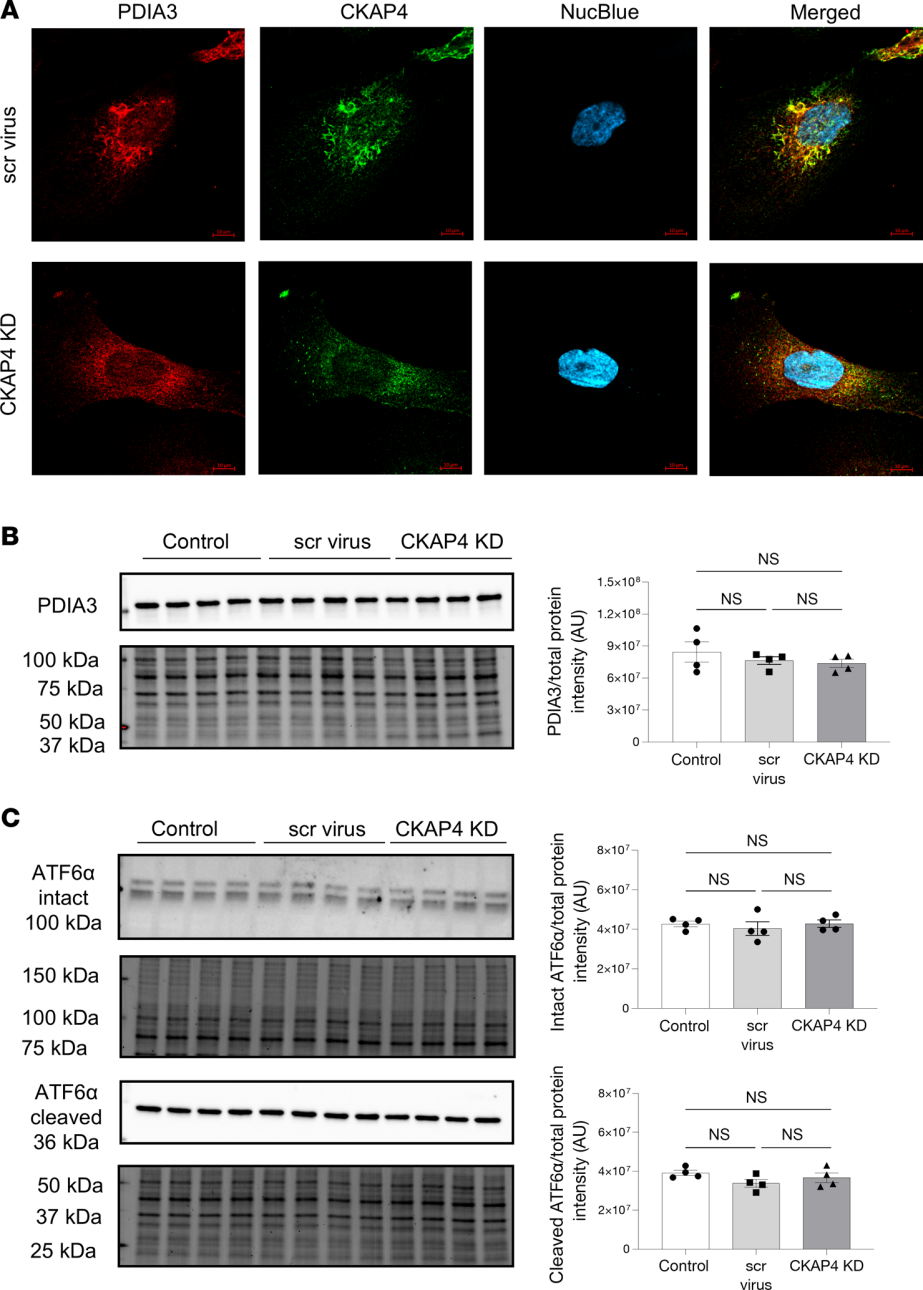
CKAP4 KD in vitro influences the shape of the ER in HPODs. Immunofluorescence staining of the ER marker PDIA3 in scr control and CKAP4 KD HPODs. Scale bars: 10 μm. (**A**). Protein expression of PDIA3 was quantified in untreated control, scr control, and CKAP4 KD HPODs using Western blot (**B**). Protein expression of ER stress marker intact and cleaved ATF6α in untreated control, scr control, and CKAP4 KD HPODs was quantified by Western blot (**C**). The total protein blots used for normalization are shown below each Western blot, and the quantification graphs are shown on the left side of the panel. **B**: *n* = 4 per group. Tukey’s post hoc after 1-way ANOVA. Error bars represent average ± SEM. AU, arbitrary units; CKAP4, cytoskeleton associated protein 4; PDIA3, protein disulfide**-**isomerase A3; ER, endoplasmic reticulum; KD, knockdown; HPODs, human podocytes; scr, scrambled; ATF6α, activating transcription factor 6α.

We examined the expression of ER marker PDIA3 using immunofluorescence and Western blot to assess ER stress levels, since the reduction of CKAP4 limits the number of anchoring points between ER and the cytoskeleton. PDIA3 distribution and hence the ER morphology was modified by CKAP4 KD, with the ER structures appearing to be irregularly dispersed in CKAP4 KD cells when compared with untreated cells (Figure 3D) and scrambled virus–treated (scr) cells (Figure 5A). However, PDIA3 protein level was not quantitatively affected by CKAP4 KD (Figure 5B). ATF6α was used to assess ER stress (Figure 5C) (29, 30). No changes in total (100 kDa) or cleaved ATFα (36 kDa) were seen, indicating that CKAP4 KD does not cause ER stress.

**Figure 6:**
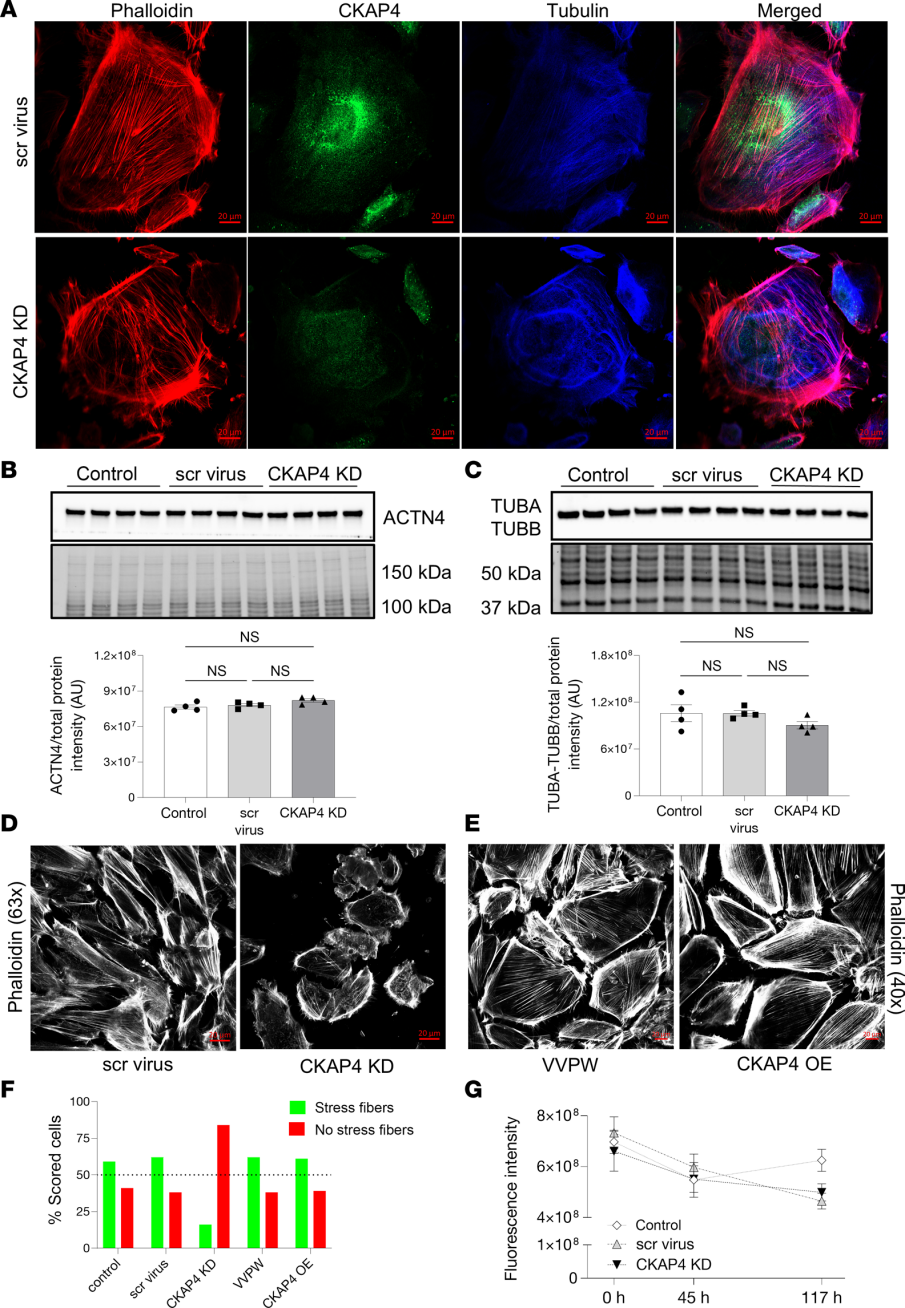
CKAP4 KD in HPODs in vitro alters the cytoskeleton. Immunofluorescence staining of HPODs with phalloidin (red, actin fibers), tubulin (blue, microtubules), and CKAP4 (green) in scr control and CKAP4 KD. HPODs showed loss of actin stress fibers and rearranged microtubules (**A**). ACTN4 (**B**) and TUBA-TUBB (**C**) protein expressions were investigated by Western blot. Total protein blots and normalized protein expression graphs are also presented. Representative pictures of phalloidin staining of scr control and CKAP4 KD HPODs (**D**) and VVPW control and CKAP4 OE (**E**). Percentage of cells scored for the presence or absence of stress fibers in both CKAP4 KD and -OE experiments (**F**). HPOD viability in untreated, scr control, and CKAP4 KD cells from transfection time to 117 hours (**G**). **F**: a minimum of 64 cells per group were scored, and percentages are reported in the graph. **G**: *n* = 8 per time point/group, Friedman’s test was used, NS. AU, arbitrary units; CKAP4, cytoskeleton associated protein 4; KD, knockdown; OE, overexpression; HPODs, human podocytes; scr, scrambled; ACTN4, α**–**actinin-4; TUBA, tubulin-α; TUBB, tubulin-β.

Anti-tubulin and phalloidin stainings were used to visualize microtubules and actin microfilaments. CKAP4 KD HPODs showed loss of microtubules traversing the cell and appearance of cortical (toward the cell periphery) and concentric structures (inside of the cell). Actin fibers were also rearranged in a cortical manner, and the stress fibers were reduced (Figure 6A). The protein levels of α–actinin 4 (ACTN4) and α- and β-tubulins (TUBA/B) showed no difference between the KD and scr cells (Figure 6, B and C), implying that the change was morphological but not quantitative.

Apart from cortical actin and reduced number of stress fibers, CKAP4 KD podocytes showed a 33% reduction in cell area in respect to scr cells (Figure 6D), while HPODs overexpressing CKAP4 presented an overall normal phenotype (Figure 6E). Cells positive for stress fibers were counted as “1,” otherwise as “0,” according to the method used by Buvall et al. (31). A minimum of 64 podocytes were evaluated at multiple random positions in each cell culture dish for each condition. The loss of stress fibers in CKAP4 KD cells was clear (Figure 6F) when compared with both untreated and scr control. Conversely, although CKAP4 overexpression (OE) was substantially increased (Supplemental Figure 1B), it did not cause stress fiber loss when compared with OE control (Figure 6F). Finally, CKAP4 KD did not affect HPODs’ viability when compared with scr and untreated cells (Figure 6G). Although a limited HPOD detachment was observed, there was no sign of apoptosis in CKAP4 KD HPODs when compared to untreated and scr controls (data not shown).

### Knockdown of CKAP4 affects cytoskeleton-related pathways.

Mass spectrometry–based (LC-MS/MS) proteomic analysis of CKAP4 KD and OE in HPODs was conducted to identify signaling pathways affected by altered CKAP4 expression. The principal component analysis (PCA) plots clearly show that only the CKAP4 KD cells could be separated from scr control and untreated cells (Supplemental Figure 2).

Ingenuity Pathway Analysis (IPA; QIAGEN) of the comparison between CKAP4 KD and scr cells revealed that 8 of the top 20 regulated pathways were related to cytoskeleton regulation (Table 2 and Supplemental Table 2).

**Figure 7:**
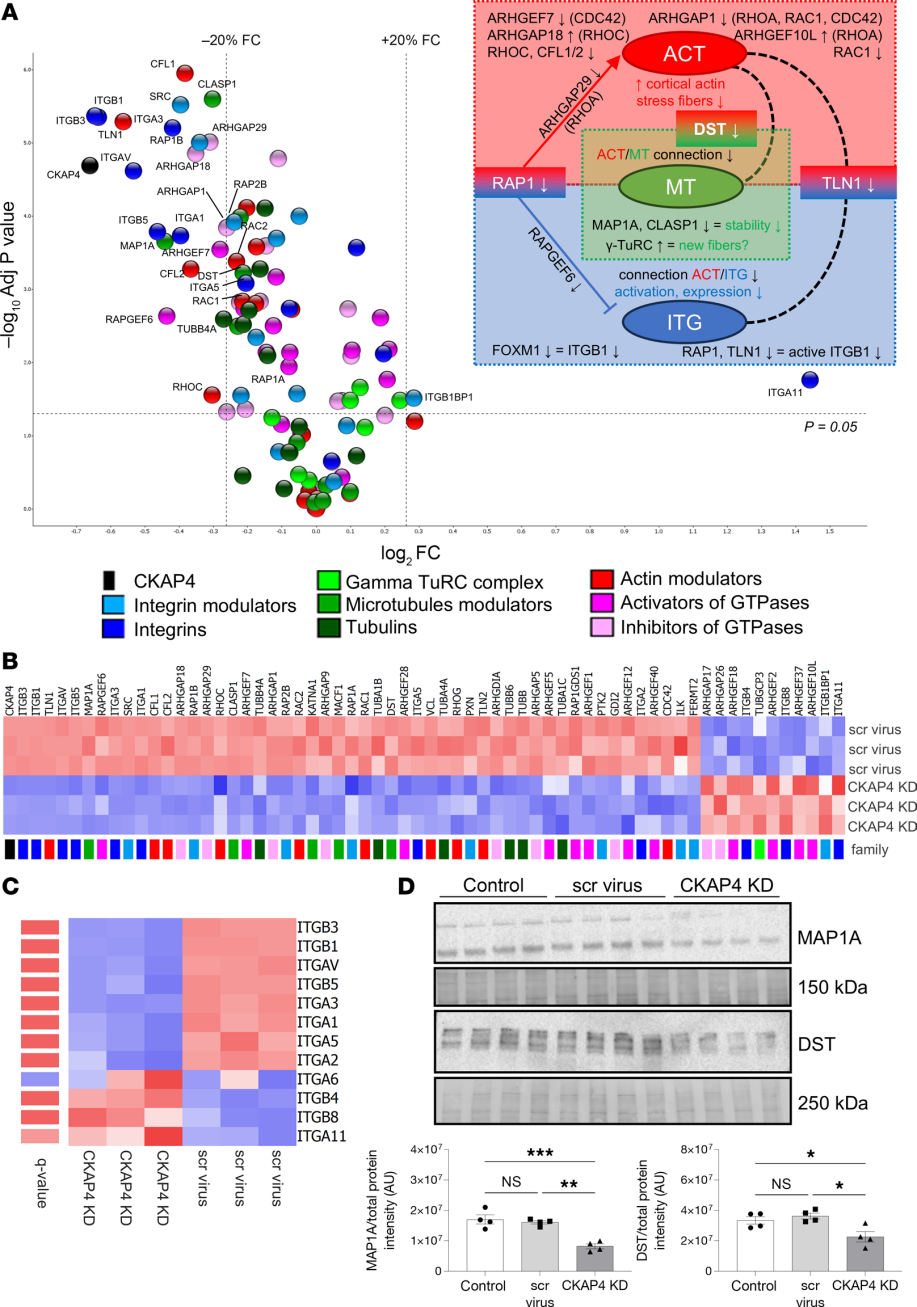
CKAP4 KD influences protein families related to cytoskeleton dynamics. Volcano plot of selected proteins from the proteomic analysis of CKAP4 KD versus scr-treated HPODs. Dotted lines represent the *P*-adjusted threshold of 0.05 (*y* axis) and fold-change thresholds of ±20% (*x* axis). Protein variations within these lines were considered not significant and/or not regulated (**A**). The diagram on the upper right side illustrates the dysregulation of ACT (actins), MT (microtubules), and ITG (integrins). Key proteins have modulatory or structural functions in between these groups: RAP1 (ACT and ITG), DST (dystonin; ATC, and MT), and TLN1 (talin 1; ITG and ACT). Heatmap of selected proteins from the proteomics analysis. The color pattern legend identifies protein families in **A** and **B**. The *P*-adjusted filter was set at 0.05, and proteins were sorted by increasing fold-change. The color scale ranging from red to blue indicates degrees of upregulation (red) and downregulation (blue) or no regulation (white) (**B**). Heatmap of all the integrins detected in CKAP4 KD, scr-treated, and untreated cells (**C**). Protein lists are given in Table 3. Integrins are ranked by increasing fold-change (KD vs. scrambled). The *q* value scale ranges from blue (NS) to red (<0.05). No *q* value threshold was imposed. Confirmation of the expression of microtubule modulators MAP1A and DST in CKAP4 KD HPODs with Western blot (**D**). Total protein blots and normalized protein expression graphs are also presented. **D**: *n* = 4 per group, Tukey’s post hoc after 1-way ANOVA. *P < 0.05, **P < 0.01, ***P < 0.001. Error bars represent average ± SEM. AU, arbitrary units; CKAP4, cytoskeleton associated protein 4; KD, knockdown; scr, scrambled; ITGA6, integrin α6; MAP1A, microtubule associated protein 1A; DST, dystonin.

The proteomic analysis identified an ongoing complex cytoskeletal remodeling in CKAP4 KD HPODs. The diagram in Figure 7A illustrates the dysregulation of actin (ACT), microtubules (MT), and integrin (ITG) dynamics happening in CKAP4 KD cells. The lists of proteins used for the proteomics analysis in Figure 7, A and B, are reported in Table 3.

While the actin cytoskeleton and the tubulins were not quantitatively regulated (Figure 7, A and B), the integrin pool was severely affected, with 8 of the 12 identified integrins significantly downregulated (Figure 7C). Integrin-related proteins were affected as well: talin (TLN1), RAP1A/B, and its modulator, RAPGEF6 (GEF, guanine nucleotide exchange factor), were all downregulated. This means that integrin activation is blocked (RAP1) and actin/integrin connection is hindered in CKAP4 KD HPODs (TLN1).

For the actin cytoskeleton, we found downregulation of ARHGEF7, ARHGAP18 (GAP, GTPase activating protein), ARHGAP29, and ARHGAP1, and upregulation of ARHGEF10L. These are all modulators of the main 3 GTPases responsible for actin dynamics (RHOA, CDC42, RAC1) in podocytes. Perturbation of GTPase activity is known to modify the shape of the actin cytoskeleton and produce a dysfunctional phenotype (32, 33). Furthermore, CFL (cofilin) 1 and 2 (that facilitate the movement of actin filaments by depolymerizing F-actin), and RHOC (a GTPase that regulates actin depolymerization), were downregulated as well, while profilin-1 (PFN1, involved in F-actin polymerization) was upregulated.

Concerning the microtubules, only TUBA4A and TUBBA4 were downregulated, but generally the pool of tubulins was stable after CKAP4 KD (see TUBA/B in Figure 6C). Three members of the γ–tubulin ring complex (γ-TuRC, which prompts de novo synthesis of microtubules) (34) showed a trend of upregulation (+15%–20% for TUBGCP3, TUBGCP5, and TUBGCP6). Interestingly, we found downregulation of microtubule-related and regulatory proteins cytoplasmic linker associated protein 1 (CLASP1), dystonin (DST), and microtubule associated protein 1A (MAP1A) in CKAP4 KD cells.

MAP1A and DST were used for a validation of the proteomics data, as the former is known to stabilize the structure of microtubules, while the latter is a cross-linker between actin fibers and microtubules. The expression of both proteins was significantly reduced in CKAP4 KD cells, as confirmed by Western blot (Figure 7D).

Thus, loss of stability and interaction with the microtubular and actin cytoskeleton, as well as loss of anchoring points (integrins), could be behind the shift in phenotype of CKAP4 KD HPODs shown in Figure 6A.

To further evaluate the connection between CKAP4 and DKD, the proteomics data were validated against glomerular transcriptomics data from patients with DKD (Supplemental Table 3) (23, 24, 35). The regulation of actin and microtubule cytoskeleton, integrins, and integrin modulators occurring in DKD glomeruli was in line with our findings in CKAP4 KD HPODs.

We conclude that CKAP4 KD negatively affects the cytoskeleton regulation in podocytes, affecting integrins and their signaling quantitatively and forcing the reshaping of actin fibers and microtubule filaments via a complex dysregulation of their modulators.

### CKAP4 KD causes a depletion of integrins.

**Figure 8:**
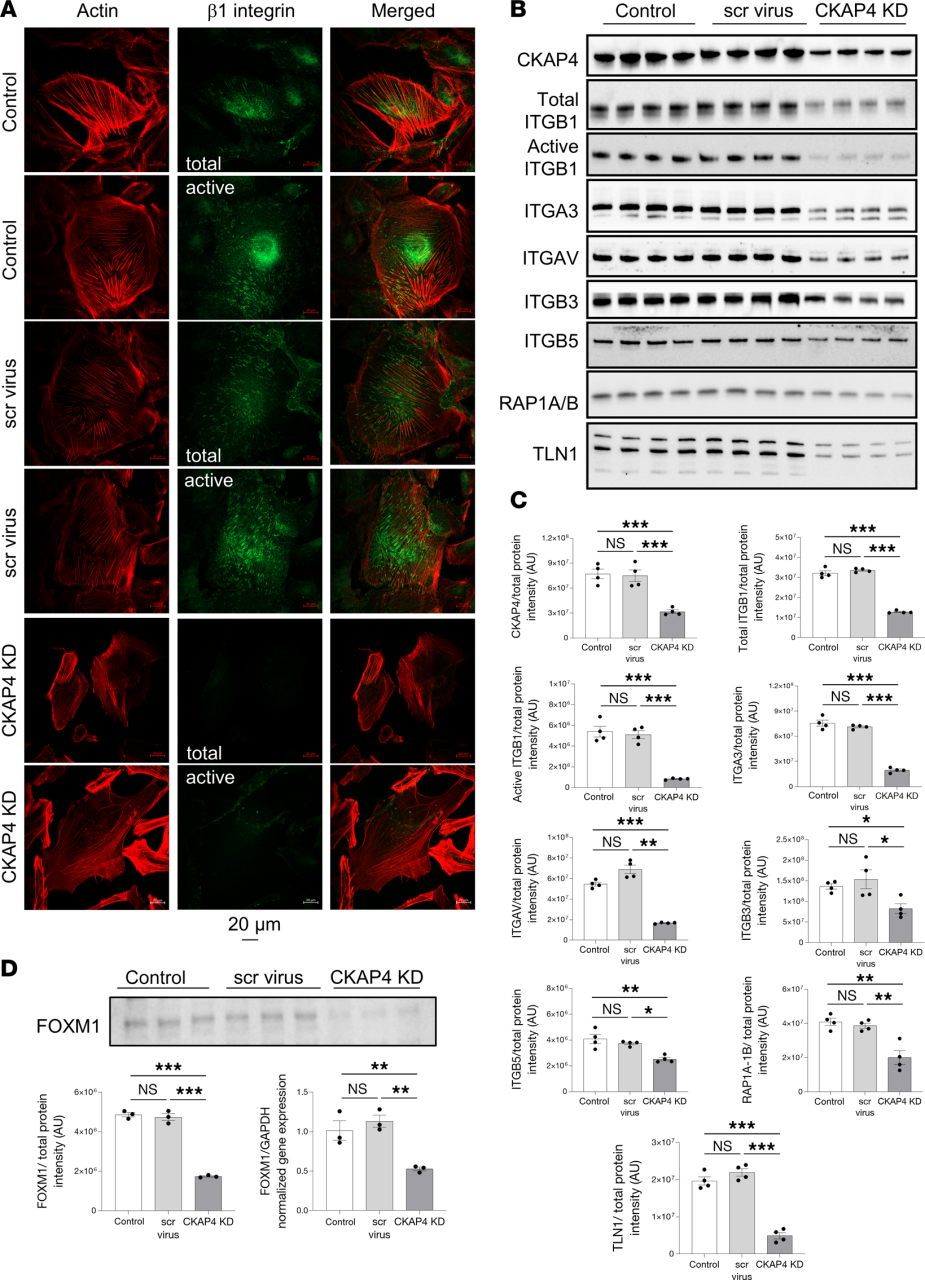
CKAP4 KD causes downregulation of integrins and influences their modulation. Immunofluorescence images of HPODs (untreated, scr-treated control, and CKAP4 KD cells) with phalloidin (actin cytoskeleton, red) and total and active β1 integrins (green) (**A**). Western blots of CKAP4, total and active ITGB1, ITGAV, ITGB3, ITGB5, RAP1A/B, and TLN1 are shown (**B**) along with the respective normalized protein expression graphs (**C**). Western blots and relative normalized protein expression graph for FOXM1, together with FOXM1 gene expression (**D**). Unedited/uncropped total protein blots used for normalization calculation are provided as supplemental materials. **C**: *n* = 4 per group, Tukey’s post hoc after 1-way ANOVA. *P < 0.05, **P < 0.01, ***P < 0.001. Error bars represent average ± SEM. **D**: *n* = 3 per group (both gene and protein expression), Tukey’s post hoc after 1-way ANOVA. **P < 0.01, ***P < 0.001. Error bars represent average ± SEM. AU, arbitrary units; CKAP4, cytoskeleton associated protein 4; FOXM1, forkhead protein M1; KD, knockdown; HPODs, human podocytes; scr, scrambled; ITGB1, integrin β1; ITGAV, integrin αV; ITGB3, integrin β3; ITGB5, integrin β5; RAP1A/B, Ras-related protein Rap-1A/B; TLN1, talin 1.

Since the pool of integrins was quantitatively affected in the CKAP4 KD, we decided to validate the decrease using both immunofluorescence imaging and Western blot and investigated the mechanism behind their reduction. Immunofluorescence showed a decrease of total and active β1 integrins in CKAP4 KD cells, alongside the previously shown actin cytoskeleton dysregulation (Figure 8A). Western blot analysis verified downregulation of integrins ITGB1 (total and active; CKAP4 KD vs. scr, KD vs. untreated control comparisons: *P* < 0.001), ITGA3 (both: *P* < 0.001), ITGAV (KD vs. scr: *P* < 0.01, KD vs. untreated: *P* < 0.001), ITGB3 (both: *P* < 0.05), ITGB5 (KD vs. scr: *P* < 0.05, KD vs. untreated: *P* < 0.01), and RAP1A/1B (KD vs. scr: *P* < 0.01, KD vs. untreated: *P* < 0.001), an activator of integrin clustering (Figure 8, B and C). TLN1, an integrin activator involved in the actin/integrin connection, was also significantly downregulated in CKAP4 KD HPODs with respect to untreated and scr cells (both, *P* < 0.001).

The transcription factor FOXM1 was downregulated at both gene (*P* > 0.001) and protein levels (*P* > 0.01) in CKAP4 KD cells compared with untreated and scr controls (Figure 8D). FOXM1 has been reported as a transcription factor for ITGB1 (36) and CKAP4 as a regulator of FOXM1 expression and activation (37–39). In conclusion, CKAP4 KD can cause a decrease in the expression of at least ITBG1 by limiting the expression of FOXM1; thus, CKAP4 KD can mechanistically affect the integrin pool.

All considered, the results strongly suggest that CKAP4 is a regulator of integrin dynamics in podocytes.

## Discussion

The podocyte cytoskeleton is vital for the maintenance of podocyte structure and function. Several glomerular diseases, such as DKD, present with podocyte FPE leading to proteinuria and subsequent loss of renal function. In this study we have investigated the cytoskeleton-related function of CKAP4 in podocytes both under physiological conditions and in disease. CKAP4 mRNA was downregulated in glomeruli from patients with DKD in 2 datasets (23, 24). This downregulation was validated in renal biopsies from patients with DKD and was not present in glomeruli from patients with IgAN. Moreover, HPODs exposed to hyperglycemia showed a significant decrease (Figure 1E) of CKAP4 expression at protein level, and a treatment with ADR, which is used as an injury model for focal segmental glomerulosclerosis, did not cause a reduction of CKAP4 in cultured HPODs.

CKAP4 was expressed by all 3 glomerular cell types, with podocytes displaying the highest expression. CKAP4 was present in the podocyte cell body, specifically colocalized with ER marker PDIA3. This is consistent with early findings about CKAP4 and its role as a linker between ER and microtubules (14).

To gain insight into the role of CKAP4 in glomerular function, the zebrafish CKAP4 homolog was knocked down. Zebrafish is a valuable model for CKD because of the simplicity of its kidney anatomy, the possibility to obtain KD via MOs, and its translatability (40–43). CKAP4 KD in zebrafish induced podocyte FPE and proteinuria, pointing at an important role for CKAP4 in podocyte function.

Next, CKAP4 was knocked down in HPODs in vitro, causing cytoskeleton rearrangement and loss of integrins. Furthermore, CKAP4 KD affected the ER shape, causing the loss of regular ER patterns, but did not cause an increment in ER stress. Proteomics analysis of CKAP4 KD HPODs verified that integrin signaling, actin, and microtubule dynamics were the main regulated pathways. The proteomics analysis was extensively validated in silico against 2 glomerular DKD transcriptomic datasets, with a clear overlap of pathways regulating cytoskeleton dynamics, as well as in vitro with immunofluorescence and Western blot.

In podocytes, the actin cytoskeleton connects to the microtubular network in the primary foot processes, linking them to the cell body. Microtubules are at one end linked to actin and at the other end anchored to the ER via CKAP4. Podocyte microtubules are arranged with a double orientation, allowing transport and elongation toward and away from the foot processes (5, 44). For instance, microtubule-based transport of Wilms tumor 1 interacting protein (WT1P) from foot processes to nuclei in podocytes has been shown in LPS-treated mice and cultured podocytes. WT1P translocation caused actin cytoskeleton rearrangement, which was transmitted directly from the periphery to the nucleus (10). CKAP4 KD in HPODs in vitro led to substantial changes in the microtubular orientation and loss of actin stress fibers. ER morphology was also affected, with loss of regular phenotype in favor of more dispersed patterns. Loss of stress fibers is a clear sign of podocyte damage. A reduction of the cellular area was also present in the CKAP4 KD HPODs, but the viability of the cells remained unchanged. The proteomics data from the CKAP4 KD HPODs revealed that 8 out of the 20 most regulated pathways were related to cytoskeleton dynamics, further supporting the connection between cytoskeleton modifications and CKAP4. CKAP4 OE did not influence the phenotype of podocytes.

To explore the processes leading to the actin cytoskeleton rearrangement, we investigated ACTN4 protein level. ACTN4 is responsible for bundling and cross-linking actin filaments (4, 45). Increased levels of ACTN4 have been reported in podocytes with FPE, and there was an association between aberrant ACTN4 forms, proteinuria, and effacement (4). No variation in ACTN4 protein expression was found; thus, the variation of morphology of the actin cytoskeleton is likely to depend on its modulators (cofilins, profilins, and small GTPase regulators), as suggested by the proteomics pathway analysis. Moreover, CKAP4 KD showed reduction of Rac-1, RhoC, and many GAPs and GEFs that regulate RhoA, Rac-1, Cdc42, and Rap-1 signaling. This might underlie the destabilization of focal adhesions, stress fibers, loss of lamellipodia structures, and alterations of the integrin clustering.

We did not observe any variation in the protein level of (total) TUBA/B. Conversely, components of the γ-TuRC, responsible for microtubular de novo generation (34), showed a trend of upregulation in the proteomics analysis. An increase of γ-TuRC would normally presuppose a concomitant global increase of all tubulins (46). However, we did not observe any increase in our experiments. A potential explanation is that podocytes react to microtubular delocalization by prompting the generation of new microtubules, hence the increase in γ-TuRC.

DST, a protein that acts as spacer and cross-linker between microtubules and actin cytoskeleton (47), was reduced in CKAP4 KD podocytes. This is consistent with findings in the glomeruli of patients with DKD (23, 24, 35). MAP1A, MAP1B, MAPT (Tau), and MAP1S are also able to link microtubules and actin. MAPT knockout causes glomerular damage and shifts podocytes toward a motile phenotype in mice, with microtubule loss by depolymerization (48, 49). Only MAP1A was identified in our proteomics dataset. MAP1A was downregulated in CKAP4 KD cells, in concordance with what was found in the DKD validation datasets.

The integrin signaling pathway was highly regulated in CKAP4 KD cells, as the integrin pool was found to be severely depleted in CKAP4 KD podocytes. Integrins in podocytes create a dynamic link to the extracellular matrix and provide attachment to the basement membrane. For instance, α_3_β_1_ integrins bind to laminin while α_1_β_1_ and α_2_β_1_ bind to collagen IV (50). In the foot processes, integrins connect directly to the actin cytoskeleton and influence podocyte structure and phenotype. In a mouse model, integrin β1 podocyte-specific KD caused progressive podocyte loss and end-stage renal failure (51). Decreased expression of α_3_β_1_ integrins has been reported in podocytes switching to a motile phenotype in vitro (52). Similar observations have been made in rat models of DKD and in diabetic patients with or without DKD (53–55). Our data reinforce these earlier findings: β1 integrin was downregulated in CKAP4 KD, and podocytes shifted toward a motile phenotype, as indicated by the loss of stress fibers. CKAP4 KD interferes with integrin activation (via TLN1 downregulation) and clustering (downregulation of RAP1 and its modulators ARHGAP29 and RAPGEF6). Moreover, TLN1 links the integrins to the actin cytoskeleton, so its reduction destabilizes the connection between these 2 cytoskeletal components. Loss of integrins might result in podocyte FPE and detachment from the glomerular basement membrane, typical hallmarks of podocyte injury in CKD (51, 56, 57).

A possible explanation for the loss of integrins in CKAP4 KD is through downregulation of transcription factor FOXM1. Expression and activation of FOXM1 appear to be regulated by CKAP4, which in turn is a transcriptional factor for ITGB1 (36–39). In CKAP4 KD cells, FOXM1 was significantly downregulated (Figure 8D) at gene and protein levels, and thus it could not be activated to stimulate integrin transcription. Interestingly, FOXM1 is downregulated in glomeruli from DKD compared with glomeruli from normal healthy kidneys (37).

To conclude, CKAP4 anchors the microtubules to the ER, stabilizing the connection between microtubules and actin. Thus, CKAP4 ensures stability of primary and secondary foot processes, and consequently it appears necessary for maintaining a functional podocyte cytoskeleton. We have shown that CKAP4 KD caused dysregulation of podocyte cytoskeleton and loss of integrins in vitro and podocyte FPE and proteinuria in vivo. CKAP4 was found to be downregulated in glomeruli derived from DKD but not in other CKDs investigated in silico and by kidney biopsy analysis. Taking everything into account, CKAP4 has the potential to be a pharmacological target for stabilizing the cytoskeleton of podocytes specifically in DKD.

## Methods

### Sex as a biological variable.

Sex was not considered as a biological variable in the zebrafish experiments. Though primordial germ cells exist in 24 hours post fertilization (hpf) embryos, no evidence of expression of genes driving differentiation of gonads or sex determination is present before day 8 post fertilization (58, 59). All experiments were completed at 120 hpf (5 days). Normal assessments for the adult (evaluation of the genital papilla; differences in color, shape, behavior; sex-linked SNP analysis; gonad dissection) could not be performed in larvae (59–61). Since normal sex ratios for zebrafish have medians around 0.5 (62, 63) we assumed a similar distribution in our population.

### In situ hybridization.

For in situ hybridization, paraffin-embedded human kidney biopsies were used. We analyzed 5 sections each from 5 patients with DKD, 4 patients with IgAN, and 5 controls (biopsies from healthy transplantation donors). Patients’ demographics for the DKD group were as follows: age 55 (47–58) (median range); sex: 4 males, 1 female; CKD stage: I (*n* = 1), III (*n* = 3), V (*n* = 1). Regarding the IgAN group we had age 73 (63–74); sex: 4 males, 1 female; CKD stage: II (*n* = 5), III (*n* = 1). Healthy control biopsies were taken from anonymous transplantation donors.

The miRCURY LNA miRNA ISH kit 8 (FFPE) was used (QIAGEN), and mRNA was stained using the hsa-CKAP4 3′ DIG probe. After deparaffinization, the sections were incubated with proteinase K (MilliporeSigma) for 10 minutes at 37°C. After 2 washes with PBS, hybridization mixture was added, and the sections were incubated for 1 hour at 55°C. The sections were washed 3 times with SSC buffer and incubated for 30 minutes with blocking solution. The sections were incubated with anti–DIG-AP (anti–digoxigenin [DIG] alkaline phosphatase [AP] conjugate) (Roche, 11093274910) for 1 hour at room temperature, washed 3 times with PBS-Tween, and incubated with freshly prepared AP reaction mixture for 2 hours at room temperature. The reaction was stopped using Potassium-Tris buffer with Triton followed by washes, and then the sections were counterstained with Nuclear Fast Red solution (Merck). After dehydration, the sections were mounted using mounting medium Prolong Antifade (Life Technologies). Images were acquired using an Axioscan 7 slide scanner (ZEISS).

Glomerular cells were scored for the presence or absence of CKAP4 mRNA. In total we scored 20 glomeruli for DKD, 22 glomeruli for IgAN, and 25 glomeruli for the control group. At least 1,400 cells were scored per group (60 cells per glomerulus on average). Sclerotic and presclerotic glomeruli (around 35% of the glomeruli in DKD and IgAN groups) were omitted from the analysis. The ratio between positive cells and total cells was used for statistical analysis.

### Immunofluorescence of kidney biopsies.

Biopsies were obtained from the healthy part of kidneys of patients undergoing nephrectomy due to tumors. Frozen human kidney sections derived from those biopsies were used for immunofluorescence. A list of antibodies is given as Supplemental Data 1. Imaging was done using an Axio Imager.Z2 LSM800 confocal microscope (ZEISS).

### Western blotting.

Cell lysis buffer was 50 mM Tris, 150 mM NaCl, and 1% Triton X-100, pH 7.5. Lysates were mixed and centrifuged (16,200*g*, 10 minutes, 4°C). The resulting supernatants were used after addition of 25% Laemmli sample buffer (Bio-Rad), addition of 10% dithiothreitol reducing agent (Invitrogen), and heating (95°C, 5 minutes). Lysates were run on stain-free TGX gels 4%–15% gradient polyacrylamide gels. Proteins were transferred to PVDF membranes using the TransBlot Turbo transfer system (Bio-Rad). Membranes were blocked in TBS with 0.1% Tween 20 (TBS-T) and 5% milk powder blotting grade blocker (Carl Roth) for 1 hour before incubation with primary antibody overnight at 4°C. Membranes were washed in TBS-T (3 times for 5 minutes) and incubated with secondary antibodies (1 hour at room temperature). After incubation, the membranes were washed again with TBS-T (3 times for 5 minutes) and developed with ECL substrate (Bio-Rad) for 5 minutes for fluorescent detection. The bands were visualized using the ChemiDoc Touch and ChemiDoc MP Imaging systems (Bio-Rad). A list of antibodies is given as Supplemental Data 1.

Stain-free total-lane or housekeeping (GAPDH) normalizations were used. Unedited blots and total lane blots used for normalization calculation are provided in the supplemental full unedited blots.

### Cell culture.

HPODs (University of Bristol) (64, 65) and primary HMCs (Cell Systems) were cultured as described previously (65, 66). Primary HGECs (Cell Systems) were cultured on attachment factor–coated (Thermo Fisher Scientific) plates in Complete Classic Medium (Cell Systems) supplemented with 10% FBS, 1% Culture boost, and 1% penicillin/streptomycin. HEK293T cells (American Type Culture Collection) were used for lentivirus production.

For the high-glucose experiments, HPODs were maintained in 1 nM insulin (Tocris Bioscience) and normal 5 mM Glc after thermoshifting the cells from 33°C to 37°C. After 2 weeks of differentiation, treatments were applied for 2 weeks, with medium changes 3 times per week. Glc was used at a 60 mM concentration. The osmotic control was normal Glc with Mtl up to 60 mM. The experiment was modeled by revisiting previously published methods (66–68). The high Glc concentration and long exposure were used to achieve a model of long-term diabetes and to overcome the issue of the slow turnover of CKAP4 that was also detected with the lentiviral knockdown.

For the ADR (doxorubicin hydrochloride, Merck D1515) experiment, HPODs were differentiated for 2 weeks before treatment. ADR was prepared in DMSO (2 mg/mL), then diluted in RPMI 1640 medium (200 μg/mL) and administered for 24 hours (69–71).

### TaqMan qPCR.

TaqMan qPCR (Thermo Fisher Scientific) was used for gene expression analysis of CKAP4 in HMCs, HGECs, and HPODs as well as for FOXM1 expression in HPODs. RNA was purified using the RNeasy Mini kit (QIAGEN), converted to cDNA using the High-Capacity RNA-to-cDNA kit (Thermo Fisher Scientific), and analyzed using the Quantstudio 7 Flex PCR system (Applied Biosystems). The endogenous control was GAPDH. All probes were from Thermo Fisher Scientific.

### Immunogold electron microscopy.

Experiments were conducted following the method of Lindström et al. (72). Biopsy tissue sections (circa 70 nm) were fixed in 0.1 M PBS, 1% paraformaldehyde, and 0.5% glutaraldehyde and processed for K11M low-temperature embedding. Anti-CKAP4 mouse primary antibody was ENZ-ABS669-0100 (Enzo Life Sciences). Section analysis was made using a Tecnai 10 microscope (FEI) at 100 kV acceleration voltage; images were captured using a Veleta camera (Olympus Soft Imaging Solutions). CKAP4 density in different areas of the podocytes was calculated by manual counting, and the areas of podocyte bodies and foot processes were measured using ImageJ (NIH). We used 15 micrographs from healthy patients’ glomeruli for the quantification.

### Zebrafish animal model: proteinuria and glomerular filtration barrier integrity tests.

*Tg(-3.5fabp10a:gc-EGFP)z*ebrafish (*Danio rerio*) were mated with zebrafish that were homozygous or heterozygous for either AB (AB fish, see https://zfin.org/ZDB-GENO-960809-7#summary for description) or the nacre (*nac^w2^*) background (nacre fish). Zebrafish were grown and mated at 28.5°C, and eggs were collected within 30 minutes of spawning, with embryos maintained and handled in standard embryo-raising media (27, 73).

The strain of zebrafish used simplifies the assessment of proteinuria (25). A fluorescent vitamin D–binding protein is expressed by the *Tg(-3.5fabp10a:gc-EGFP)* zebrafish, easy to monitor in the retinal vessel plexus. This systemic fluorescence increases over time. If the glomerular filtration barrier is damaged, the fish loses plasma proteins, resulting in fluorescence decrease. Proteinuria was measured by reduction of eye fluorescence using an Axiovert 200 microscope (ZEISS). Maximum fluorescence intensity was analyzed in gray scale using ImageJ by using the outer circle measurement of the eye (25, 27).

TEM was used to examine the morphology of the glomerular filtration barrier (120 hpf). Larvae were fixed in solution D overnight at 4°C, washed 3 times in 0.1 M cacodylate buffer (pH 7.4), and postfixed in 1% OsO_4_ for 1 hour. Tissues were subsequently washed, dehydrated, and embedded in EPON and hardened at 60°C for more than 16 hours. Sections were then prepared for TEM by staining with uranyl acetate (2%) for 30 minutes and lead citrate for 15 minutes. We cut 90 nm sections of the glomerular region with a microtome and transferred them to copper grids. Podocyte FPE percentages were calculated according to the method described by Müller-Deile et al. (28).

### Zebrafish animal model: exclusion criteria and power calculations.

The following exclusion criteria were used: absence of “red sac” after MO injection at 0 hpf (MO is injected with Phenol Red; hence, a successful injection is identified by a “red sac” slowly diffusing to the yolk, as per ref. 74); dead embryo at 48 hpf; no flow at 48 hpf.

The ClinCalc tool at https://clincalc.com/stats/samplesize.aspx was used for a priori sample size calculation. The average measurements of max eye fluorescence from the MO control group at 96 hpf was used. As it was impossible to predict the extent of the KD, a small effect size was predicted (15% reduction), with α = 0.05 and power = 80%. The number of animals per groups needed was at least 25. The reduction detected after the experiment was performed was more than 25% (30 μM ckap4 MO vs. control MO).

### Zebrafish animal model, MO ckap4 KD, and confirmation with proteomics.

ATG-blocking MOs were used at 30, 50, and 75 μM concentrations. The zebrafish functional homolog to human CKAP4 (75) was knocked down using the anti-*ckap4* MO (5′ AGAGAGATGGCTTGAACTCCC 3′). An MO targeting a human intron mutation causing beta-thalassemia was used as negative control (5′ CCTCTTACCTCAGTTACAATTTATA 3′). Both MOs were from Gene Tools (Philomath). The MOs were injected using a Nanoject II injection drive (Drummond Scientific) into 1- to 4-cell zebrafish embryos (27). A minimum of 32 larvae per group was injected with MOs.

*Ckap4* KD was confirmed at protein level using proteomics, since there are no commercially available antibodies for zebrafish *ckap4*. Three pools of untreated, 75 μM control MO–treated, and 75 μM CKAP4 MO–treated larvae (*n* > 8) were collected at 96–120 hpf. The pooled larvae were washed in PBS and then submitted to lysis using 2.5 mm ceramic beads in SDS gel loading buffer. A total of 10 μg of each respective protein lysate was run on 10% NuPAGE Tris Glycine gel and digested with trypsin as previously described (76). A nano LC-MS/MS (Dionex Ultimate 3000 RLSCnano) interfaced with an Eclipse Orbitrap (both from Thermo Fisher Scientific) was used for the mass spectrometry analysis using a data-independent acquisition workflow: Resolution was 12,000, automatic gain control (AGC) set at 3 million, ion time as auto, and MS scan range 400–1,200. The ions in C-trap were sequentially isolated with 8 *m/z* windows, with AGC at 400,000 and ion time set to auto. The ions were fragmented using a relative collision energy of 30. MS/MS scans were recorded with a resolution of 30,000. Raw data were analyzed using the recommended setting in the DIA-NN 1.8.1 software (https://github.com/vdemichev/DiaNN), in library-free mode (search against a sequence database, *Danio rerio* UniProt proteome library) and with an FDR of 1% (77). The results were analyzed using Qlucore omics explorer (version 3.9, Qlucore). A protein was considered significantly regulated when fold-change was ±20%, with an FDR-adjusted *P* value (*q* value) of *q* < 0.05.

### shRNA silencing and overexpression of CKAP4 in vitro in HPODs.

KD and OE of CKAP4 were accomplished using lentiviral transfection. For KD, shRNA targeting CKAP4 mRNA (target sequence: GCAGGATTTGAAAGCCTTAAA), inserted in the pLKO.1 cloning vector, was used (Sigma-Aldrich) (78). The pLKO.1 vector with nonsilencing (scr) shRNA was used as a virus control. For OE, the CKAP4 gene was amplified with PCR using the pCMV6-AC-CKAP4-GFP vector (Origene). The obtained cDNA was ligated into a VVPW-EGFP vector, placing the CKAP4 gene at the C-terminus of EGFP to generate a fusion protein with EGFP at the N-terminus (cytosolic) of CKAP4. VVPW (79, 80) is a lentiviral expression vector (Virus Vector) containing the protein kinase G promoter region and the cis-acting posttranscriptional regulatory element of the woodchuck hepatitis virus WPRE (PKG, WPRE promoters). The VVPW-EGFP vector was a gift from Anna Greka at Brigham and Women’s Hospital and Harvard Medical School, Boston, Massachusetts, USA.

The VVPW-EGFP (simply reported as VVPW in the figures) vector without insert was used as control. All vectors were amplified in *E*. *coli* and purified using the HiSpeed Plasmid Maxi kit (QIAGEN). Lentivirus were produced in HEK293T cells by addition of envelope (CMV), packaging (VSVG), and transfer plasmid (pLKO.1 or VVPW with or without insert) to cell culture medium (DMEM 4.5 g/L Glc, Lonza) supplemented with 10% FBS. FuGene 6 (Promega) was used as transfection reagent. The virus-containing medium was collected 72 hours posttransfection and stored at –80°C.

HPODs were allowed to differentiate for 7 days at 37°C before transduction. HPODs were exposed for 16 hours to a batch-dependent concentration of virus-containing medium (8%–33%) using 4 μg/mL hexadimethrine bromide. HPODs were kept in culture for 7 days before the experiments.

### Immunofluorescence staining and evaluation of stress fiber integrity after CKAP4 KD and OE.

HPODs were fixed (4% paraformaldehyde, 4% sucrose in PBS, 10 minutes), permeabilized (0.3% Triton-x in PBS, 10 minutes, 4°C), and then blocked (PBS, 2% FBS, 2% BSA, 0.2% fish gelatin). The antibodies used were: Alexa Fluor 594 anti-phalloidin (actin microfilaments/stress fibers, A12381, Invitrogen), Alexa Fluor 647 anti-tubulin (microtubules, Abcam, ab195884), AMAb90988 for PDIA3 (Atlas antibodies), and HPA000792 for CKAP4 (Atlas antibodies). Secondary antibodies coupled with Alexa Fluor 488 (A11034), 594 (A11012), or 647 (A21244) were obtained from Invitrogen. Either ProLong Diamond Antifade with or without DAPI (Life Technologies) or ProLong glass Anti-fade with NucBlue (Thermo Fisher Scientific) were used for mounting.

For the actin microfilament morphology, HPODs were scored according to the method used by Buvall et al. (31). HPODs were counted as “1” when the actin fibers (stress fibers) were spanning the whole surface of the cell, otherwise as “0.” For each condition at least 60 podocytes were evaluated at multiple random positions in each culture dish using an Axio Imager.Z2 LSM800 confocal microscope (ZEISS), at 40× or 63× original magnification.

### Podocyte viability after CKAP4 KD.

HPOD cell viability after CKAP4 KD was investigated using Alamar blue (Invitrogen). Cells were incubated with Alamar blue solution at 37°C, and fluorescence was measured using SpectraMax i3 plate reader (Molecular Devices).

### Mass spectrometry of protein expression in CKAP4 KD podocytes.

An Orbitrap Fusion Tribrid mass spectrometer interfaced to an Easy-nLC1000 (Thermo Fisher Scientific) was used for the proteomics analysis. Proteome Discoverer version 1.4 (Thermo Fisher Scientific) was used for identification of the detected proteins. Database searches were performed by Mascot search engine (Matrix Science Ltd) using the SwissProt *Homo sapiens* protein database. The results were analyzed using Qlucore omics explorer versions 3.8 and 3.9.

Statistical analysis of LC-MS/MS data followed the protocol from Liu et al. (81). Sample expression ratios were calculated based on average quantity of all the untreated samples. Data were evaluated using density plot and histograms to ensure the distribution properties. Group clustering was checked using PCA and hierarchical clustering. Multiple Student’s 2-tailed *t* tests were performed with Benjamini-Hochberg FDR correction (set at 5%). Since a technical variance of 10% could be observed using isobaric mass tagging reagent, ±20% cutoff on the unlogged fold-change was used. Comparisons with other omics datasets (23, 24, 35) were performed using Qlucore omics explorer (version 3.8 and 3.9). Significantly regulated proteins were analyzed with IPA 2.3 (QIAGEN).

### Statistics.

Normality was checked with Shapiro-Wilk test and variance equality with Levene’s test in GraphPad Prism (versions 8–10, GraphPad Software), SPSS (version 23, IBM), or Qlucore omics explorer. Statistical differences were investigated with 2-tailed Student’s *t* test or Mann-Whitney tests or using 1-way ANOVA and Kruskal-Wallis tests when comparing 3 or more groups. Error bars represent SEM or SD. Refer to each respective figure legend for more details.

### Study approval.

This study was performed in accordance with the NIH *Guide for the Care and Use of Laboratory Animals* (National Academies Press, 2011). Use of human material was approved by the regional ethical board of Gothenburg (413-09 and 110-98). Written informed consent was collected before the collection of biopsies. Zebrafish experiments were conducted at Mount Desert Island Biological Laboratory (MDIBL) and approved by the local IACUC (17-03). Animal Research: Reporting of In Vivo Experiments (ARRIVE) reporting guidelines were used (82).

### Data availability.

All data generated or analyzed are included in this article and its supplement. A Supporting Data Values spreadsheet is provided, containing data values for every figure and supplemental figure. For a color-blind–friendly version of the figures in this manuscript, we suggest the use of the free cvd emulator online tool at http://hclwizard.org:3000/cvdemulator/

HPOD CKAP4 KD proteomics data have been deposited to PRIDE repository (http://www.ebi.ac.uk/pride, with dataset identifier PXD046643) and the zebrafish proteomics to massIVE (https://massive.ucsd.edu/ProteoSAFe/static/massive.jsp, with dataset identifier MSV000093243). Both repositories are affiliated to the proteomeXchange consortium (https://www.proteomexchange.org). See Supplemental Data 2.

Validation datasets used in this study are available at http://karokidney.org/rna-seq-dn (23), https://www.ncbi.nlm.nih.gov/geo/ (GSE30122) (24), and https://www.ncbi.nlm.nih.gov/geo/ (GSE47185) (35). The latter two are also available at https://www.nephroseq.org

## Author contributions

KE and JN conceptualized the study. RB and PL curated the proteomics dataset. Validation of proteomics data was performed by RB. EL, KE, and JN acquired fundings. Formal data analysis was performed by RB, EL, PL, AC, and KE. Investigation was performed by RB, EL, AJ, PL, AC, RT, JMD, and KE. RT performed experiments at Gothenburg University. Project administration duties were fulfilled by KE and JN. Lab resources were offered by JMD, MS, KE, and JN. Supervision was provided by JMD, MS, KE, and JN. Data visualization was performed by RB, EL, PL, and KE. The original draft was prepared by EL. All the authors participated in writing, reviewing, and editing the manuscript. CRediT classification (https://credit.niso.org/) was used to assess the contributions.

RB and EL share first authorship based upon their equally important contributions to the project. First authorship order was based on the amount of work contributed to the study. EL initiated the study and performed experiments. RB expanded the study and performed revision experiments and data analysis.

## Supplementary Material

Supplemental data

Supplemental data set 1

Supplemental data set 2

Unedited blot and gel images

Supporting data values

## Figures and Tables

**Table 3:**
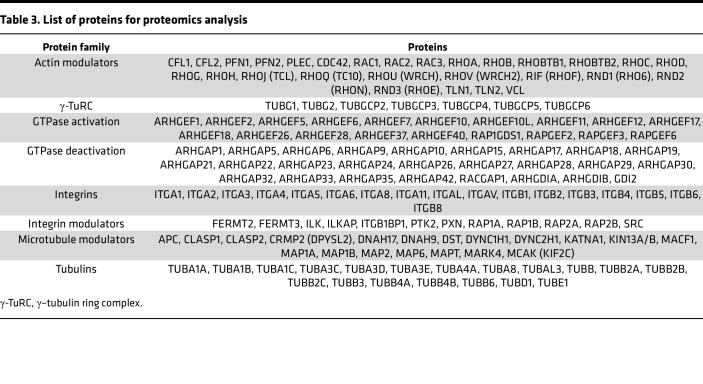
List of proteins for proteomics analysis

**Table 1:**
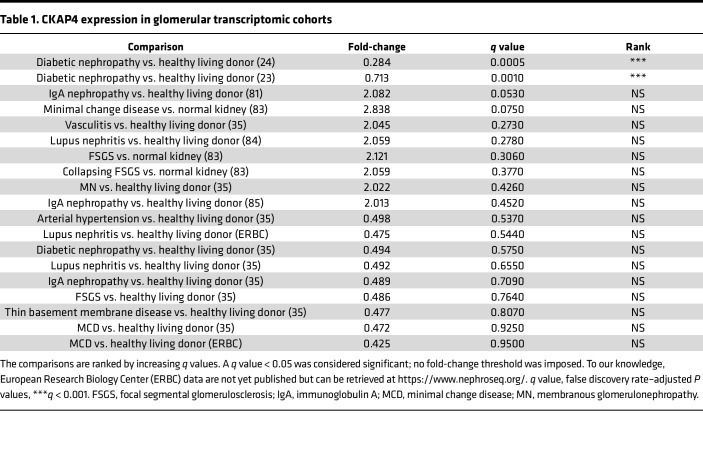
CKAP4 expression in glomerular transcriptomic cohorts

**Table 2:**
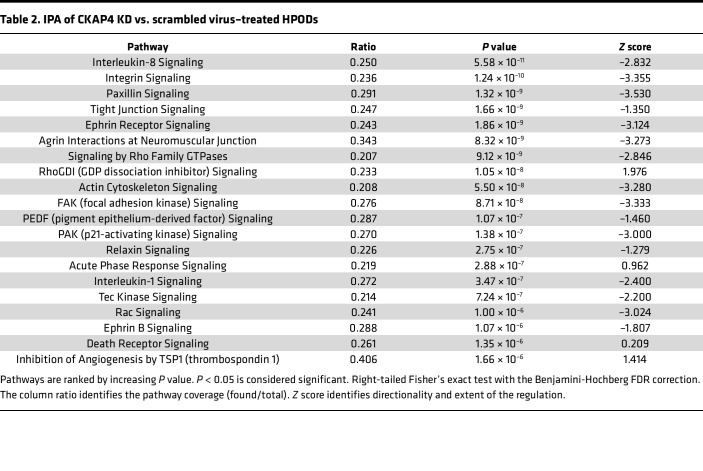
IPA of CKAP4 KD vs. scrambled virus–treated HPODs

## Version 1 06/10/2025

In-Press Preview

## Version 2 07/22/2025

Electronic publication
