# Supplementary material for: Cytoskeleton-associated protein 4 affects podocyte cytoskeleton dynamics in diabetic kidney disease
Source: JCI Insight. 2025 Jun 10;10(14):e181298. doi: 10.1172/jci.insight.181298 (PMC12288980; doi:10.1172/jci.insight.181298)
Supplement: Supplemental data [file jciinsight-10-181298-s178.pdf]

SUPPLEMENTAL MATERIALS

Cytoskeleton-associated protein 4 (CKAP4) affects podocyte cytoskeleton dynamics in diabetic kidney disease.

Roberto Boi,[1] Emelie Lassén,[1] Alva Johansson,[1] Peidi Liu,[1] Aditi Chaudhari,[1] Ramesh Tati,[2] Janina Müller-Deile,[3] Mario Schiffer, [3,4] Kerstin Ebefors,[1] and Jenny Nyström.[1]

Conflict of interest statement

RT, EL, AC are employed at AstraZeneca. EL and AC were not employed at AstraZeneca at the time the experiments were performed. RT experiments were performed at Gothenburg University. Except for this, the authors have declared that no conflict of interest exists.

Address correspondence to: Jenny Nyström, Institute of Neuroscience and Physiology, Department of Physiology, Sahlgrenska Academy, University of Gothenburg, Box 432, 40530, Gothenburg, Sweden. jenny.nystrom@gu.se

Authorship note: RB and EL share first authorship of the manuscript.

[1] Institute of Neuroscience and Physiology, Sahlgrenska Academy, Gothenburg University, Sweden
[2] Bioscience Renal, Research and Early Development, Cardiovascular, Renal and Metabolism, BioPharmaceuticals R&D, AstraZeneca, Gothenburg, Sweden.
[3] Department of Nephrology, Friedrich-Alexander-Universität Erlangen-Nürnberg, Erlangen, Germany
[4] Mount Desert Island Biological Laboratory, Salisbury Cove, Maine, USA.

Supplemental Table of contents

Supplementary Figures

1. Confirmation of knockdown and overexpression of CKAP4

2. PCA plot and cluster dendrogram plot of CKAP4 KD proteomics dataset

Supplementary Tables

1. CKAP4 proof of knockdown in zebrafish (intensities of all tryptic fragments)

2. Full Ingenuity Pathway Analysis of the proteomic dataset

3. Validation of proteomic data with transcriptomic dataset

Supplemental Figure 1. Confirmation of knockdown and overexpression of CKAP4.

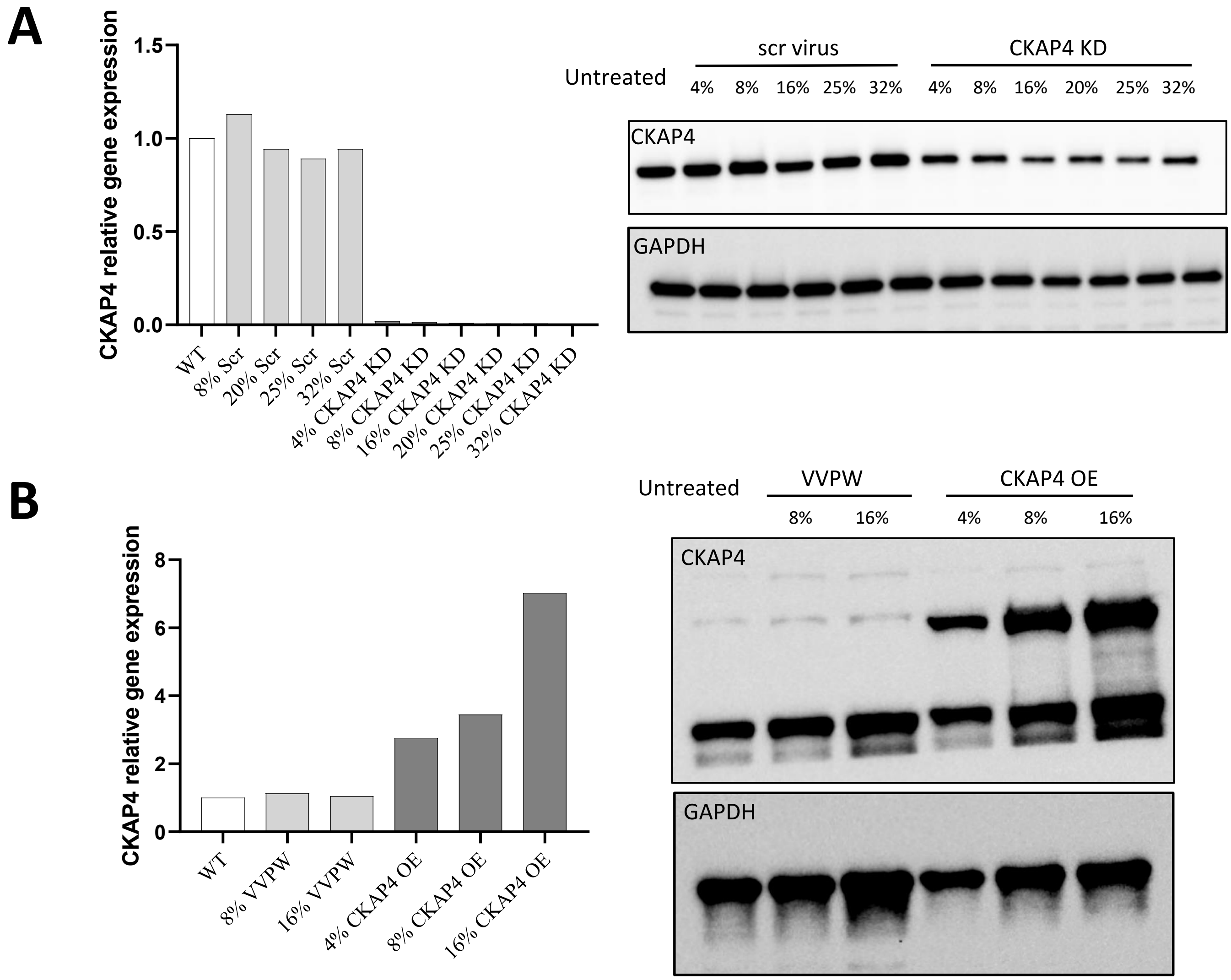

Knockdown of CKAP4 in HPODs with different percentages of virus was confirmed at mRNA (left panel) and protein level (right panel) (A). Overexpression of CKAP4 in HPODs with different percentages of overexpression construct was confirmed at mRNA (left panel) and protein level (right panel) (B). Normalization was obtained using GAPDH as housekeeping protein. CKAP4, cytoskeleton associated protein 4; GAPDH, glyceraldehyde 3-phosphate dehydrogenase; HPODs, human podocytes; KD, knockdown; OE, overexpression.

Supplemental Figure 2. PCA plot and cluster dendrogram plot of CKAP4 KD proteomics dataset

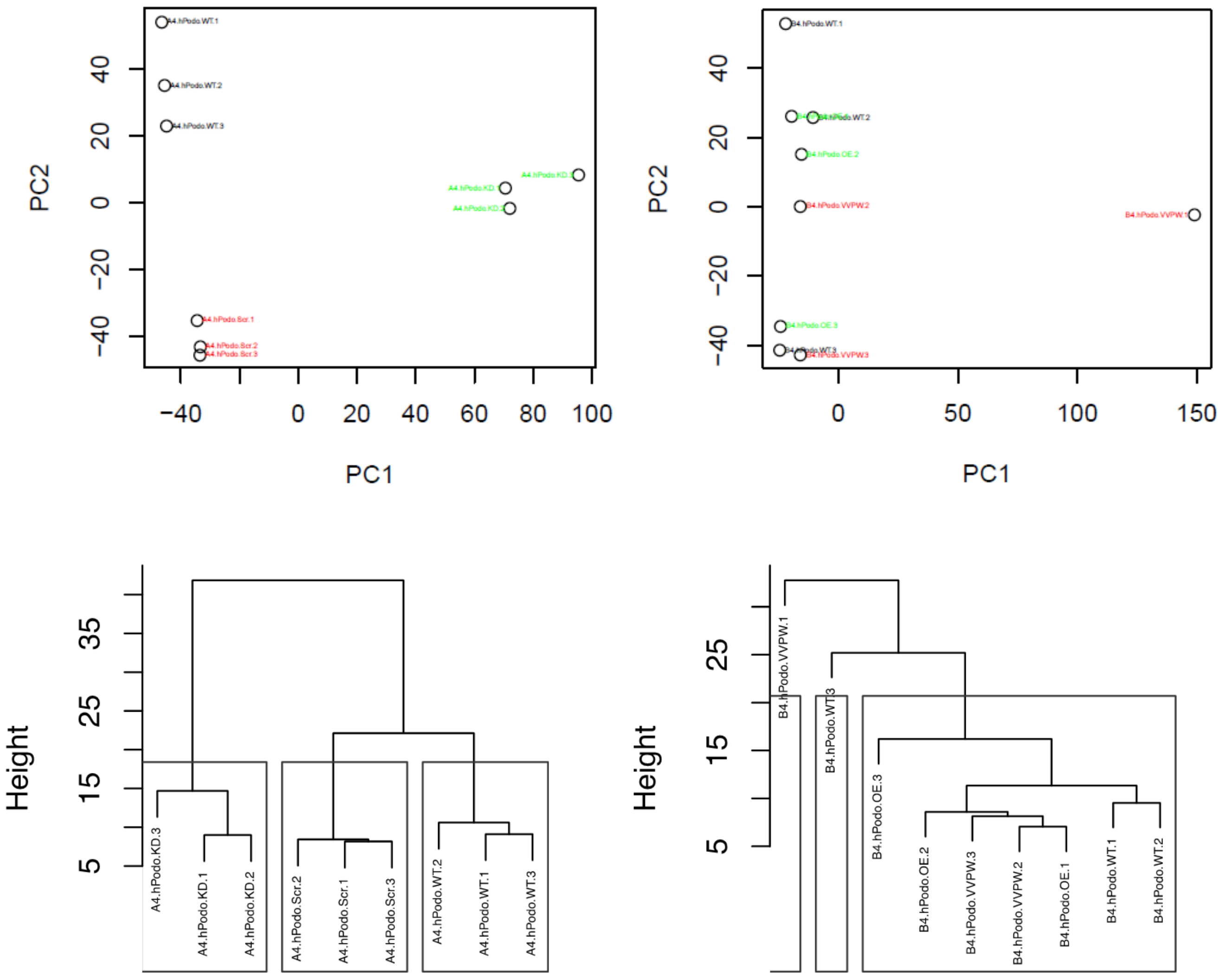

Principal component analysis (PCA, upper panels) and cluster dendrograms (lower panels) of the CKAP4 knockdown (KD) and CKAP4 overexpression (OE) cells, their relative scrambled virus or VVPW vector controls and wild type cells. PCA and dendrograms show separation between the treatment groups in the KD cells (left panels) experiment but no clustering in the OE cells (right panels). CKAP4, cytoskeleton associated protein 4; PC, principal component.

Supplemental Table 1. CKAP4 proof of knockdown in zebrafish (intensities of all tryptic fragments)

Fragment	MO control 1	MO control 2	MO control 3	MO CKAP4 KD 1	MO CKAP4 KD 2	MO CKAP4 KD 3	wild type 1	wild type 2	wild type 3
ASLEGLEDAVALTR	3.65E+06	3.77E+06	3.42E+06				3.07E+06	2.60E+05	3.76E+06
C(UniMod:4)SGAAQELEEALEQLR	8.80E+05	7.70E+05	1.03E+06		3.54E+05		9.28E+05	8.65E+05	7.64E+05
EIALLEER	4.02E+06	3.69E+06	4.06E+06	2.04E+05		1.57E+05	3.56E+06	3.95E+06	3.84E+06
EQISAVSAELQTR	2.25E+06	2.38E+06	2.17E+06	1.78E+05		1.32E+05	2.08E+06	2.52E+06	2.25E+06
EQQSFKEDLQELR	1.12E+06	8.41E+05	7.09E+05				7.64E+05	1.03E+06	8.55E+05
EVQSVSEEIK	2.22E+06	1.92E+06	2.06E+06	1.56E+05	1.26E+05	1.13E+05	1.99E+06	2.05E+06	2.01E+06
KTVPELSLR	3.86E+05	4.11E+05	4.26E+05				3.17E+05	3.71E+05	4.33E+05
LEDTLQGLQK	1.96E+05	1.94E+05	1.46E+05				1.11E+05	1.64E+05	1.67E+05
LQAAVEEIR	3.34E+06	3.14E+06	3.48E+06				2.70E+06	3.48E+06	2.96E+06
LQEQTDAR	2.91E+05	1.88E+05	2.16E+05					1.84E+05	2.15E+05
QDQLSDALHSLDTR	1.47E+06	1.52E+06	1.55E+06			7.51E+04	1.34E+06	1.52E+06	1.52E+06
RLEDTLQGLQK	1.08E+06	1.31E+06	1.27E+06	1.28E+05			1.02E+06	1.16E+06	1.21E+06
SADLQELR	2.75E+06	2.65E+06	3.04E+06				2.19E+06	2.67E+06	2.75E+06
SNSASSGGPAPEESGK	2.59E+05	2.50E+05	2.47E+05		9.41E+03		1.82E+05	3.08E+05	2.37E+05
SRIEELQR	1.16E+05	1.03E+05	1.27E+05				1.13E+05	9.63E+04	1.10E+05
SSAQTSADQAQSFILK	1.73E+06	1.75E+06	1.83E+06				1.47E+06	1.67E+06	1.53E+06
SVQQGELSTR	6.84E+05	3.88E+05	4.95E+05	5.07E+04			9.07E+05	4.59E+05	5.95E+05
TLIQSTVGSLR	3.85E+06	3.62E+06	3.87E+06				3.32E+06	4.08E+06	4.05E+06
TLIQSTVGSLRK	6.90E+05	5.91E+05	5.43E+05	1.49E+04	3.37E+04	3.04E+04	5.45E+05	4.57E+05	5.88E+05
TSSGHEQALGR	6.24E+04	5.77E+04	6.29E+04				5.16E+04	6.89E+04	5.69E+04
TVPELSLR	3.88E+06	3.72E+06	3.80E+06	2.96E+05	2.61E+05	2.08E+05	3.82E+06	3.41E+06	3.76E+06
**SUM (intact CKAP4 estimate levels)**	3.49E+07	3.33E+07	3.46E+07	1.03E+06	7.84E+05	7.15E+05	3.05E+07	3.08E+07	3.37E+07

CKAP4, cytoskeleton associated protein 4; KD, knockdown; MO, morpholino. UniMod:4 refers to Iodoacetamide modification.

Supplemental Table 2. IPA analysis of the proteomic dataset

Ingenuity Canonical Pathways	-log(p-value)	Ratio	z-score	Proteins
Molecular Mechanisms of Cancer	11.500	0.204	NaN	RAP2B,CDKN2A,PRKACB,RAC2,TGFBR1,SMAD3,TAB2,CDK9,CCND1,TGFBR2,RB1,PAK1,ITGA3,BBC3,CCND3,TGFB1,MAP3K7,MRAS,IRS2,NFKBIB,ATM,E2F4,CCNE2,CASP3,FGFR1,RAC1,CDK6,ITGA5,MAPK12,RAP1A,RASGRF2,CCND2,PRKCD,IRS1,GNAO1,PRKACA,FZD6,CFLAR,CDK2,RAP1B,RELA,ARHGEF7,NFKBIE,GNA11,CTNNA1,ABL1,PSEN2,SMAD5,WNT7A,CDK17,PRKAR1B,TGFB2,BID,AKT3,ARHGEF2,PIK3R2,BMP1,ITGB1,SRC,PAK4,PIK3C2A,GRB2,RHOC,GNA12,DVL1,PRKAR2A,ADCY6,GNAI1,GNAI2,GNAI3,PLCB4,RRAS2,MAPK14,NF1,PAK2,CDKN1B,CASP7,FZD7,CTNND1
Germ Cell-Sertoli Cell Junction Signaling	11.300	0.272	NaN	RAC2,MAP3K11,TGFBR1,CTNNA1,MAP3K4,IQGAP1,LIMK1,TGFBR2,PAK1,ITGA3,CFL2,SORBS1,TGFB1,TGFB2,MRAS,MAP3K7,TUBB4A,IRS2,PIK3R2,JUP,VCL,MAP3K2,ATM,ITGB1,SRC,PAK4,EPN1,PIK3C2A,CFL1,TJP1,RHOC,GRB2,FGFR1,ACTB,RAC1,TUBA4A,MAPK12,CDH2,RRAS2,MAPK14,IRS1,PAK2,A2M,ACTG1,NECTIN2,CTNND1
Role of Tissue Factor in Cancer	11.000	0.308	NaN	CTGF,GNA11,LIMK1,ITGB3,PAK1,ITGA3,ARRB1,CFL2,MRAS,ITGAV,AKT3,FGB,IRS2,PIK3R2,ITGB5,ATM,EGFR,ITGB1,SRC,CASP3,PIK3C2A,CFL1,GRB2,GNA12,FGFR1,RAC1,PLAUR,RPS6KA5,MAPK12,ARRB2,RRAS2,MAPK14,PDXP,IRS1,LYN,IL1B,CYR61
IL-8 Signaling	10.600	0.250	-2.832	RAC2,CCND1,LIMK1,ITGB3,CCND3,ITGAV,MRAS,IRS2,NFKBIB,GNG12,ITGB5,ATM,FGFR1,GNG2,RAC1,MAPK12,PLD1,GNG10,MYL9,ARRB2,CCND2,ARAF,PRKCD,IRS1,RELA,IQGAP1,PDGFC,EIF4EBP1,HMOX1,AKT3,PIK3R2,CHUK,GNG5,VASP,EGFR,SRC,VCAM1,PIK3C2A,RHOC,GRB2,GNA12,GNAI1,IKBKE,GNAI2,ROCK1,GNAI3,RRAS2,PAK2,MAP4K4
Axonal Guidance Signaling	9.920	0.186	NaN	DPYSL2,PRKACB,RAC2,PFN1,MYL6,EPHB2,NTN1,LIMK1,NCK2,PAK1,ITGA3,CFL2,MRAS,IRS2,CHMP1A,GNG12,ATM,PAPPA,KALRN,CFL1,ADAMTS1,FGFR1,GNG2,RAC1,ITGA5,L1CAM,DPYSL5,RAP1A,PDGFB,GNG10,MYL9,SRGAP3,ARPC1A,SDCBP,PRKCD,IRS1,GNAO1,PRKACA,FZD6,RAP1B,ERAP2,MMP7,ARHGEF7,GNA11,ABL1,ROBO1,PDGFC,NFAT5,WNT7A,PPP3CB,SDC2,NGFR,PRKAR1B,PLXNB1,AKT3,TUBB4A,SEMA3B,ERBB2,PIK3R2,GNG5,SHANK2,VASP,BMP1,ITGB1,PAK4,PIK3C2A,NRP2,GRB2,GNA12,PRKAR2A,TUBA4A,GNAI1,ROCK1,GNAI2,GNAI3,PLCB4,RRAS2,PAK2,EPHA5,SEMA3C,ADAM9,SEMA7A,FZD7
Integrin Signaling	9.790	0.236	-3.355	RAP2B,RAC2,PFN1,MAP3K11,PIKFYVE,TLN1,ITGB3,NCK2,TSPAN3,ITGA3,PAK1,ITGA11,ITGAV,MRAS,IRS2,ITGB5,ATM,CAPN5,FGFR1,RAC1,ITGA5,TTN,RAP1A,PDGFB,MYL9,ARPC1A,ARF5,IRS1,ITGA1,ACTG1,RAP1B,ARHGEF7,ABL1,ITGB8,PPP1R12A,AKT3,PIK3R2,VCL,VASP,ITGB1,SRC,PAK4,PIK3C2A,RHOC,GRB2,ACTB,ROCK1,RRAS2,PAK2,NEDD9
Paxillin Signaling	8.880	0.291	-3.530	ARHGEF7,TLN1,ITGB8,ITGB3,NCK2,ITGA3,PAK1,ITGA11,MRAS,ITGAV,IRS2,PIK3R2,VCL,ITGB5,ATM,ITGB1,SRC,PAK4,PIK3C2A,GRB2,ACTB,FGFR1,RAC1,ITGA5,MAPK12,GIT2,MAPK14,RRAS2,IRS1,PAK2,ITGA1,ACTG1
Tight Junction Signaling	8.780	0.247	-1.350	PRKACB,RELA,TGFBR1,MYL6,VAPA,PPP2R5B,CTNNA1,CPSF4,OCLN,TGFBR2,TGFB1,NGFR,CGN,TGFB2,PRKAR1B,AKT3,ARHGEF2,GOSR1,VCL,BET1L,NAPB,VASP,TNFRSF11B,YKT6,TJP1,ACTB,CRB3,RAC1,PRKAR2A,YBX3,CASK,MYL9,F11R,CLDN1,PPP2R3A,PRKACA,PATJ,GOSR2,ACTG1,PPP2R1B,NECTIN2
Ephrin Receptor Signaling	8.730	0.243	-3.124	RAP1B,RAC2,EPHB2,GNA11,ABL1,PDGFC,LIMK1,NCK2,PAK1,ITGA3,CFL2,SORBS1,SDC2,MRAS,AKT3,GNG5,GNG12,ITGB1,SRC,PAK4,CFL1,KALRN,GRB2,GNA12,GNG2,RAC1,GNAI1,ITGA5,RAP1A,PDGFB,GNG10,GNAI2,ROCK1,GNAI3,RRAS2,SDCBP,ARPC1A,PAK2,GNAO1,EPHA5,DOK1,MAP4K4
Agrin Interactions at Neuromuscular Junction	8.080	0.343	-3.273	ITGB1,SRC,RAC2,PAK4,ACTB,ARHGEF7,DVL1,RAC1,ITGA5,MAPK12,ITGB3,LAMC1,ITGA3,PAK1,RRAS2,PAK2,UTRN,MRAS,ITGA1,ERBB2,AGRN,ACTG1,EGFR
Signaling by Rho Family GTPases	8.040	0.207	-2.846	MAP3K11,MYL6,DIAPH3,PIKFYVE,PIP4K2B,LIMK1,ITGA3,PAK1,CFL2,MRAS,CDC42EP1,IRS2,GNG12,ATM,CFL1,FGFR1,GNG2,RAC1,ITGA5,MAPK12,PLD1,GNG10,MYL9,CDH2,ARPC1A,IRS1,GNAO1,ACTG1,RELA,PPP1R12C,ARHGEF7,WASF3,GNA11,IQGAP1,PPP1R12A,PIP5KL1,ARHGEF2,PIK3R2,GNG5,ITGB1,PAK4,PIK3C2A,RHOC,GRB2,ACTB,GNA12,GNAI1,CDH6,GNAI2,ROCK1,GNAI3,PAK2
RhoGDI Signaling	7.980	0.233	1.976	PPP1R12C,MYL6,ARHGEF7,GNA11,PIKFYVE,PIP4K2B,LIMK1,PAK1,ITGA3,CFL2,PPP1R12A,MRAS,ARHGAP12,PIP5KL1,ARHGEF2,GNG5,GNG12,ITGB1,SRC,PAK4,CFL1,WASF2,RHOC,GNA12,ACTB,GNG2,RAC1,GNAI1,CDH6,ITGA5,GNG10,MYL9,GNAI2,ROCK1,GNAI3,CDH2,ARPC1A,GNAO1,PAK2,ARHGAP1,ACTG1

Ingenuity Canonical Pathways	-log(p-value)	Ratio	z-score	Proteins
Actin Cytoskeleton Signaling	7.260	0.208	-3.280	RAC2,FN1,PFN1,MYL6,DIAPH3,ARHGEF7,PIKFYVE,PIP4K2B,TRIO,TLN1,IQGAP1,PDGFC,LIMK1,PAK1,ITGA3,CYFIP2,CFL2,PPP1R12A,INS,MRAS,IRS2,PIK3R2,VCL,TMSB10/TMSB4X,GNG12,ATM,ITGB1,PAK4,PIK3C2A,CFL1,WASF2,GRB2,GNA12,FGFR1,ACTB,RAC1,ITGA5,TTN,PDGFB,MYL9,ROCK1,RRAS2,ARPC1A,IRS1,PAK2,ACTG1
Sertoli Cell-Sertoli Cell Junction Signaling	7.220	0.225	NaN	PRKACB,MAP3K11,CTNNA1,MAP3K4,OCLN,MPP6,ITGA3,SORBS1,CGN,MRAS,PRKAR1B,MAP3K7,AKT3,TUBB4A,JUP,VCL,GUCY1B1,MAP3K2,SPTBN2,ITGB1,SRC,DLG1,EPN1,TJP1,ACTB,RAC1,TUBA4A,PRKAR2A,ITGA5,YBX3,MAPK12,F11R,MAPK14,RRAS2,CLDN1,PRKACA,A2M,ACTG1,NECTIN2
FAK Signaling	7.060	0.276	-3.333	CAPN5,ITGB1,SRC,PAK4,PIK3C2A,GRB2,FGFR1,ARHGEF7,ACTB,RAC1,ITGA5,TLN1,GIT2,PAK1,ITGA3,RRAS2,IRS1,PAK2,MRAS,AKT3,IRS2,PIK3R2,VCL,TNS1,ACTG1,ATM,EGFR
Breast Cancer Regulation by Stathmin1	6.990	0.211	NaN	PRKACB,CAMK1D,ARHGEF7,PPP2R5B,LIMK1,PAK1,PPP1R12A,PPP1R10,RB1CC1,MRAS,PRKAR1B,ARHGEF2,TUBB4A,IRS2,PIK3R2,GNG5,GNG12,ATM,PPP1R14C,E2F4,CCNE2,PIK3C2A,GRB2,FGFR1,GNG2,RAC1,TUBA4A,ADCY6,GNAI1,PRKAR2A,GNG10,GNAI2,ROCK1,GNAI3,PLCB4,RRAS2,PPP2R3A,PRKCD,IRS1,PRKACA,CDKN1B,PPP2R1B,CDK2
PEDF Signaling	6.970	0.287	-1.460	RELA,WASF2,PIK3C2A,GRB2,FGFR1,NFKBIE,RAC1,SERPINF1,IKBKE,MAPK12,ROCK1,HNF1B,SOD2,RRAS2,MAPK14,IRS1,MRAS,AKT3,IRS2,PIK3R2,CHUK,CFLAR,NFKBIB,CASP7,ATM
PAK Signaling	6.860	0.270	-3.000	ITGB1,PAK4,CASP3,MYL6,PIK3C2A,CFL1,GRB2,FGFR1,ARHGEF7,RAC1,ITGA5,MAPK12,PDGFC,PDGFB,LIMK1,NCK2,MYL9,PAK1,ITGA3,RRAS2,CFL2,IRS1,PAK2,MRAS,IRS2,PIK3R2,ATM
Relaxin Signaling	6.560	0.226	-1.279	RAP1B,PRKACB,RELA,NFKBIE,GNA11,GDE1,MRAS,PRKAR1B,AKT3,SMPDL3B,IRS2,PIK3R2,GNG5,PDE11A,NFKBIB,GNG12,GUCY1B1,ATM,SMPDL3A,PIK3C2A,GRB2,GNA12,FGFR1,GNG2,ADCY6,GNAI1,PRKAR2A,RAP1A,GNG10,PDE1C,GNAI2,GNAI3,IRS1,GNAO1,PRKACA
Acute Phase Response Signaling	6.540	0.219	0.962	IL6ST,RELA,ITIH3,FN1,NFKBIE,HMOX1,SOD2,ITIH2,NGFR,SAA1,MRAS,CFB,MAP3K7,AKT3,SERPINA1,FGB,PIK3R2,CHUK,SERPINE1,NFKBIB,TNFRSF11B,C3,GRB2,AHSG,SERPINF1,IKBKE,CEBPB,SAA2,MAPK12,HMOX2,SERPINF2,ALB,MAPK14,RRAS2,TF,IL1B,A2M
IL-1 Signaling	6.460	0.272	-2.400	PRKACB,RELA,GNA12,NFKBIE,GNG2,GNA11,TAB2,PRKAR2A,GNAI1,ADCY6,IKBKE,MAPK12,GNG10,GNAI2,GNAI3,MAPK14,GNAO1,PRKACA,MAP3K7,PRKAR1B,MRAS,CHUK,GNG5,NFKBIB,GNG12
Clathrin-mediated Endocytosis Signaling	6.430	0.204	NaN	PICALM,EPHB2,ITGB8,PDGFC,ITGB3,AP1G2,ARRB1,SNX9,PPP3CB,INS,AAK1,DAB2,SERPINA1,IRS2,PIK3R2,ITGB5,ATM,MYO6,ITGB1,SRC,EPN1,PIK3C2A,GRB2,ACTB,FGFR1,RAC1,RAB7A,ITGA5,PDGFB,APOL1,SH3GL1,ARRB2,ALB,LDLR,ARPC1A,TF,IRS1,TFRC,ACTG1,CLU,HIP1R,APOC3
Tec Kinase Signaling	6.140	0.214	-2.200	RELA,GNA11,TNFSF10,PAK1,ITGA3,MRAS,IRS2,PIK3R2,GNG5,TNFRSF10A,GNG12,ATM,ITGB1,SRC,PAK4,PIK3C2A,RHOC,GRB2,GNA12,FGFR1,ACTB,GNG2,TNFRSF10B,GNAI1,ITGA5,MAPK12,GNG10,GNAI2,GNAI3,PRKCD,IRS1,GNAO1,PAK2,LYN,STAT2,ACTG1
Rac Signaling	6.000	0.241	-3.024	RELA,MAP3K11,PIKFYVE,PIP4K2B,IQGAP1,LIMK1,ITGA3,PAK1,CYFIP2,CFL2,MRAS,PIP5KL1,IRS2,PIK3R2,ATM,ITGB1,PAK4,CFL1,PIK3C2A,GRB2,FGFR1,RAC1,ITGA5,PLD1,ARPC1A,RRAS2,IRS1,PAK2
Ephrin B Signaling	5.970	0.288	-1.807	RAC2,KALRN,CFL1,EPHB2,GNA12,GNG2,GNA11,RAC1,GNAI1,GNG10,LIMK1,ROCK1,NCK2,GNAI2,GNAI3,PAK1,CFL2,GNAO1,MRAS,GNG5,GNG12
Virus Entry via Endocytic Pathways	5.960	0.245	NaN	RAC2,HLA-B,ABL1,ITGB8,ITGB3,ITGA3,AP1G2,MRAS,IRS2,PIK3R2,ITGB5,ATM,ITGB1,SRC,PIK3C2A,GRB2,ACTB,FGFR1,RAC1,ITGA5,FOLR1,RRAS2,IRS1,PRKCD,TFRC,ITGA1,ACTG1
Death Receptor Signaling	5.870	0.261	0.209	RELA,CASP3,ACTB,NFKBIE,PARP10,TNFRSF10B,TNFSF10,IKBKE,PARP12,TBK1,LIMK1,ROCK1,TANK,TNKS,BID,DFFB,CFLAR,CHUK,NFKBIB,ACTG1,MAP4K4,TNFRSF10A,CASP7,PARP14
Inhibition of Angiogenesis by TSP1	5.780	0.406	1.414	HSPG2,TGFBR1,SDC1,CASP3,MAPK12,TGFBR2,CD47,MAPK14,TGFB1,SDC2,THBS1,AKT3,GUCY1B1

Ingenuity Canonical Pathways	-log(p-value)	Ratio	z-score	Proteins
NF-κB Activation by Viruses	5.770	0.264	-2.711	ITGB1,RELA,PIK3C2A,GRB2,NFKBIE,FGFR1,ITGA5,IKBKE,ITGB3,ITGA3,RRAS2,PRKCD,IRS1,ITGAV,MRAS,AKT3,ITGA1,IRS2,PIK3R2,CHUK,NFKBIB,ITGB5,ATM
CCR3 Signaling in Eosinophils	5.730	0.230	-2.183	LIMK1,PAK1,PPP1R12A,CFL2,MRAS,IRS2,PIK3R2,GNG5,GNG12,ATM,PAK4,CFL1,PIK3C2A,GRB2,FGFR1,GNG2,RAC1,GNAI1,MAPK12,GNG10,GNAI2,ROCK1,GNAI3,PLCB4,MAPK14,RRAS2,IRS1,PRKCD,PAK2
Pancreatic Adenocarcinoma Signaling	5.680	0.233	-2.065	CDKN2A,RELA,TGFBR1,SMAD3,ABL1,CCND1,PDGFC,TGFBR2,RB1,HMOX1,TGFB1,TGFB2,AKT3,IRS2,PIK3R2,ERBB2,EGFR,ATM,E2F4,PIK3C2A,GRB2,FGFR1,RAC1,MAPK12,PLD1,IRS1,CDKN1B,CDK2
NGF Signaling	5.680	0.233	-1.347	RAP1B,RELA,MAP3K11,TRIO,MAP3K4,NGFR,MAP3K7,MRAS,AKT3,IRS2,CHUK,PIK3R2,ATM,MAP3K2,PIK3C2A,GRB2,FGFR1,RAC1,IKBKE,RPS6KA5,MAPK12,RAP1A,SMPD2,ROCK1,RRAS2,IRS1,PRKCD,MAPK7
HER-2 Signaling in Breast Cancer	5.670	0.261	NaN	ITGB1,TSC1,CCNE2,PIK3C2A,GRB2,FGFR1,CDK6,PARD6B,ITGB8,CCND1,ITGB3,RRAS2,PRKCD,IRS1,MRAS,AKT3,IRS2,ERBB2,PIK3R2,CDKN1B,ITGB5,EGFR,ATM
Leukocyte Extravasation Signaling	5.590	0.194	-3.244	RAP1B,RAC2,MMP7,MYL6,ABL1,CTNNA1,MAP3K4,ITGB3,ITGA3,ARHGAP12,IRS2,PIK3R2,VCL,VASP,TIMP2,ATM,ITGB1,SRC,VCAM1,PIK3C2A,GRB2,FGFR1,ACTB,RAC1,GNAI1,ITGA5,MAPK12,RAP1A,GNAI2,ROCK1,F11R,GNAI3,MAPK14,CLDN1,PRKCD,IRS1,ITGA1,ARHGAP1,ACTG1,CTNND1
NF-κB Signaling	5.520	0.202	-0.845	PRKACB,AZI2,RELA,TRAF3,TGFBR1,NFKBIE,TAB2,TGFBR2,TANK,TNIP1,CARD10,NGFR,UBE2V1,INS,MRAS,MAP3K7,AKT3,IRS2,PIK3R2,CHUK,NFKBIB,TNFRSF11B,EGFR,ATM,PIK3C2A,GRB2,FGFR1,TBK1,IGF2R,TAB3,RRAS2,ARAF,IRS1,PRKACA,IL1B,MAP4K4
AMPK Signaling	5.510	0.191	0.169	PRKACB,CAB39,TSC1,RAB9B,PPP2R5B,CCND1,ELAVL1,EIF4EBP1,GYS1,CRTC2,INS,MAP3K7,MRAS,PRKAR1B,AKT3,IRS2,PIK3R2,EEF2K,AKT1S1,ATM,SRC,PFKFB3,RAB27A,CPT1A,PIK3C2A,GRB2,FGFR1,ACTB,PRKAR2A,RAB7A,MAPK12,PFKFB2,PFKM,MAPK14,AK5,PPP2R3A,IRS1,SIRT1,PRKACA,HMGCR,PPP2R1B
CXCR4 Signaling	5.500	0.207	-1.300	MYL6,GNA11,PAK1,MRAS,AKT3,IRS2,PIK3R2,GNG5,GNG12,ATM,SRC,PAK4,PIK3C2A,RHOC,GRB2,GNA12,FGFR1,GNG2,RAC1,ADCY6,GNAI1,MAPK12,GNG10,MYL9,GNAI2,ROCK1,GNAI3,PLCB4,RRAS2,PRKCD,IRS1,GNAO1,PAK2,LYN
Production of Nitric Oxide and Reactive Oxygen Species in Macrophages	5.500	0.197	-0.667	RAP1B,RELA,MAP3K11,NFKBIE,PPP2R5B,MAP3K4,PPP1R10,PPP1R12A,NGFR,MAP3K7,AKT3,SERPINA1,IRS2,PIK3R2,CHUK,NFKBIB,MAP3K2,TNFRSF11B,ATM,PPP1R14C,PIK3C2A,RHOC,GRB2,FGFR1,RAC1,IKBKE,MAPK12,RAP1A,APOL1,ALB,MAPK14,PPP2R3A,PRKCD,IRS1,PPP2R1B,CLU,SIRPA,APOC3
Regulation of Cellular Mechanics by Calpain Protease	5.420	0.309	-2.138	CAPN5,ITGB1,SRC,GRB2,CDK6,ITGA5,TLN1,CCND1,RB1,ITGA3,RRAS2,MRAS,CAST,VCL,CDKN1B,CDK2,EGFR
Small Cell Lung Cancer Signaling	5.380	0.259	-1.414	RELA,CCNE2,TRAF3,PIK3C2A,GRB2,FGFR1,NFKBIE,CDK6,ABL1,IKBKE,CCND1,RB1,IRS1,BID,AKT3,IRS2,PIK3R2,CDKN1B,CHUK,NFKBIB,CDK2,ATM
Chronic Myeloid Leukemia Signaling	5.310	0.238	NaN	CDKN2A,RELA,E2F4,TGFBR1,PIK3C2A,GRB2,FGFR1,SMAD3,ABL1,CDK6,IKBKE,CCND1,TGFBR2,RB1,RRAS2,TGFB1,IRS1,TGFB2,MRAS,AKT3,IRS2,PIK3R2,CDKN1B,CHUK,ATM
Macropinocytosis Signaling	5.170	0.259	-2.138	ITGB1,SRC,PIK3C2A,GRB2,FGFR1,RAC1,ITGA5,ITGB8,PDGFC,PDGFB,ITGB3,PAK1,RRAS2,PRKCD,IRS1,INS,MRAS,IRS2,PIK3R2,ITGB5,ATM
ERK/MAPK Signaling	5.170	0.191	-2.333	RAP1B,PRKACB,RAC2,YWHAH,PPP2R5B,TLN1,EIF4EBP1,KSR1,PAK1,ITGA3,PPP1R12A,PPP1R10,MRAS,PRKAR1B,IRS2,PIK3R2,ATM,ITGB1,PPP1R14C,SRC,PAK4,ATF1,PIK3C2A,GRB2,FGFR1,RAC1,PRKAR2A,ITGA5,RPS6KA5,RAP1A,RRAS2,ARAF,PPP2R3A,PRKCD,IRS1,PAK2,PRKACA,PPP2R1B
HGF Signaling	5.130	0.228	-2.353	RAP1B,CDKN2A,ITGB1,MAP3K11,PIK3C2A,GRB2,FGFR1,RAC1,ITGA5,MAP3K4,MAPK12,CCND1,RAP1A,PAK1,ITGA3,RRAS2,PRKCD,IRS1,MRAS,MAP3K7,AKT3,IRS2,PIK3R2,CDK2,MAP3K2,ATM
Semaphorin Signaling in Neurons	5.110	0.308	NaN	DPYSL2,ITGB1,PAK4,CFL1,DPYSL3,RHOC,RAC1,DPYSL5,LIMK1,ROCK1,PAK1,CFL2,PAK2,PLXNB1,ARHGAP1,SEMA7A
Caveolar-mediated Endocytosis Signaling	4.960	0.268	NaN	ITGB1,SRC,ACTB,HLA-B,ABL1,ITGA5,ITGB8,ITGB3,ALB,ITGA3,ITGA11,INS,ITGAV,ITGA1,CAVIN1,ACTG1,ITGB5,MAP3K2,EGFR

Ingenuity Canonical Pathways	-log(p-value)	Ratio	z-score	Proteins
B Cell Receptor Signaling	4.960	0.191	-3.000	RAP1B,RAP2B,RELA,RAC2,MAP3K11,NFKBIE,ABL1,INPPL1,MAP3K4,NFAT5,CFL2,PPP3CB,CARD10,MRAS,MAP3K7,AKT3,IRS2,PIK3R2,CHUK,NFKBIB,MAP3K2,ATM,CFL1,PIK3C2A,GRB2,FGFR1,RAC1,IKBKE,MAPK12,RAP1A,SYNJ2,MAPK14,RRAS2,IRS1,PAG1,LYN
Colorectal Cancer Metastasis Signaling	4.880	0.177	-0.949	IL6ST,PRKACB,RELA,MMP7,TGFBR1,SMAD3,CCND1,PDGFC,TGFBR2,APPL1,ARRB1,WNT7A,TGFB1,TGFB2,MRAS,PRKAR1B,AKT3,IRS2,PIK3R2,GNG5,GNG12,ATM,EGFR,SRC,E2F4,CASP3,PIK3C2A,RHOC,GRB2,FGFR1,GNG2,DVL1,RAC1,ADCY6,PRKAR2A,MAPK12,GNG10,RRAS2,IRS1,PRKACA,MSH6,FZD6,FZD7
PPARα/RXRα Activation	4.840	0.196	-0.180	PRKACB,RELA,TGFBR1,NFKBIE,SMAD3,GNA11,ADIPOR1,TGFBR2,GPD2,TGFB1,INS,TGFB2,MRAS,PRKAR1B,MAP3K7,CHUK,NFKBIB,ITGB5,GRB2,ACOX1,ADCY6,PRKAR2A,NR2C2,IKBKE,AIP,PLCB4,RRAS2,MAPK14,IRS1,PRKACA,IL1B,NCOR2,MAP4K4
PTEN Signaling	4.780	0.218	2.600	RAC2,RELA,TGFBR1,YWHAH,INPPL1,CCND1,TGFBR2,ITGA3,NGFR,MRAS,AKT3,CHUK,PIK3R2,EGFR,ITGB1,SHARPIN,CASP3,GRB2,FGFR1,RAC1,ITGA5,IKBKE,IGF2R,SYNJ2,RRAS2,CDKN1B
Regulation of Actin-based Motility by Rho	4.740	0.244	-1.789	ITGB1,RAC2,PAK4,PFN1,MYL6,CFL1,RHOC,ACTB,RAC1,PIKFYVE,ITGA5,PIP4K2B,LIMK1,ROCK1,MYL9,ITGA3,PAK1,ARPC1A,PPP1R12A,PAK2,PIP5KL1
LPS-stimulated MAPK Signaling	4.660	0.241	-1.964	RELA,PIK3C2A,ATF1,GRB2,NFKBIE,FGFR1,RAC1,IKBKE,MAPK12,PAK1,MAPK14,RRAS2,PRKCD,IRS1,MAP3K7,MRAS,IRS2,PIK3R2,CHUK,NFKBIB,ATM
IL-6 Signaling	4.640	0.211	-0.962	IL6ST,RELA,NFKBIE,NGFR,MRAS,MAP3K7,AKT3,IRS2,PIK3R2,CHUK,NFKBIB,ATM,TNFRSF11B,ABCB1,PIK3C2A,GRB2,FGFR1,IKBKE,CEBPB,MAPK12,COL1A1,MAPK14,RRAS2,IRS1,IL1B,A2M,MAP4K4
Renal Cell Carcinoma Signaling	4.620	0.247	-2.324	PAK4,PIK3C2A,GRB2,FGFR1,RAC1,RAP1A,PDGFB,ARNT,PAK1,RRAS2,CUL2,TGFB1,IRS1,PAK2,MRAS,AKT3,IRS2,PIK3R2,VHL,ATM
Ovarian Cancer Signaling	4.600	0.203	-0.655	PRKACB,CDKN2A,MMP7,ABL1,CCND1,PDGFC,RB1,WNT7A,ARRB1,MRAS,PRKAR1B,AKT3,IRS2,PIK3R2,EGFR,ATM,SRC,PIK3C2A,GRB2,FGFR1,PTGS1,DVL1,PRKAR2A,RRAS2,IRS1,PRKACA,FZD6,MSH6,FZD7
Epithelial Adherens Junction Signaling	4.600	0.203	NaN	RAP1B,TGFBR1,MYL6,CTNNA1,IQGAP1,TGFBR2,SORBS1,MRAS,TGFB2,AKT3,TUBB4A,VCL,JUP,EGFR,SRC,EPN1,ACTB,FGFR1,RAC1,TUBA4A,RAP1A,PTPRM,MYL9,CDH2,ARPC1A,RRAS2,ACTG1,CTNND1,NECTIN2
PI3K/AKT Signaling	4.510	0.211	-1.225	TSC1,RELA,YWHAH,NFKBIE,PPP2R5B,GDF15,INPPL1,CCND1,EIF4EBP1,ITGA3,GYS1,MRAS,AKT3,CHUK,PIK3R2,NFKBIB,ITGB1,RHEB,GRB2,ITGA5,IKBKE,SYNJ2,RRAS2,PPP2R3A,CDKN1B,PPP2R1B
GP6 Signaling Pathway	4.450	0.206	-2.502	RAP1B,COL8A1,TLN1,COL4A2,ITGB3,LAMC1,COL5A1,COL6A1,AKT3,IRS2,FGB,PIK3R2,ATM,LAMA5,COL4A1,PIK3C2A,GRB2,COL12A1,FGFR1,RAC1,COL1A1,IRS1,PRKCD,LYN,LAMA3,COL11A1,COL3A1
Angiopoietin Signaling	4.410	0.247	-0.832	RELA,PAK4,PIK3C2A,GRB2,FGFR1,NFKBIE,IKBKE,TNIP1,PAK1,RRAS2,IRS1,PAK2,MRAS,AKT3,IRS2,PIK3R2,CHUK,NFKBIB,ATM
Glioblastoma Multiforme Signaling	4.410	0.193	-1.890	CDKN2A,TSC1,NF2,CCND1,PDGFC,RB1,WNT7A,MRAS,AKT3,IRS2,PIK3R2,EGFR,ATM,SRC,E2F4,PIK3C2A,RHOC,GRB2,FGFR1,RAC1,CDK6,PDGFB,PLCB4,RRAS2,NF1,PRKCD,IRS1,FZD6,CDKN1B,CDK2,FZD7
p53 Signaling	4.380	0.216	-0.471	CDKN2A,PIK3C2A,GRB2,FGFR1,TNFRSF10B,CCND1,TP53BP2,SERPINE2,SCO2,RB1,MAPK14,BBC3,CCND2,THBS1,PPP1R13B,IRS1,SIRT1,AKT3,IRS2,PIK3R2,TNFRSF10A,CDK2,DRAM1,ATM
GNRH Signaling	4.350	0.191	-1.826	PRKACB,RELA,MAP3K11,GNA11,MAP3K4,CACNA1F,PAK1,PRKAR1B,MRAS,MAP3K7,GNG5,MAP3K2,EGFR,SRC,PAK4,GRB2,GNG2,RAC1,ADCY6,GNAI1,PRKAR2A,MAPK12,GNAI2,GNAI3,PLCB4,MAPK14,RRAS2,PRKCD,PAK2,PRKACA,MAPK7
Antiproliferative Role of TOB in T Cell Signaling	4.330	0.385	NaN	PABPC1,TGFBR2,RB1,CCNE2,TGFBR1,TGFB1,SMAD3,TGFB2,CDKN1B,CDK2
CD40 Signaling	4.330	0.244	0.000	RELA,TRAF3,PIK3C2A,ATF1,GRB2,FGFR1,NFKBIE,PTGS1,IKBKE,MAPK12,TANK,MAPK14,IRS1,MAP3K7,IRS2,PIK3R2,CHUK,NFKBIB,ATM
CDK5 Signaling	4.330	0.224	-1.789	ITGB1,PRKACB,PPP1R14C,LAMA5,PPP2R5B,PRKAR2A,ABL1,ADCY6,MAPK12,LAMC1,ITGA3,MAPK14,RRAS2,PPP1R10,PPP1R12A,PPP2R3A,NGFR,PRKACA,PRKAR1B,MRAS,MAPK7,PPP2R1B

Ingenuity Canonical Pathways	-log(p-value)	Ratio	z-score	Proteins
ILK Signaling	4.320	0.181	-2.263	RELA,FN1,MYL6,PPP2R5B,ITGB8,CCND1,PDGFC,ITGB3,NCK2,TGFB1I1,CFL2,PPP1R12A,AKT3,IRS2,PIK3R2,VCL,TMSB10/TMSB4X,ITGB5,ATM,ITGB1,FBLIM1,CASP3,CFL1,PIK3C2A,RHOC,GRB2,ACTB,FGFR1,RPS6KA5,MAPK12,MYL9,PPP2R3A,IRS1,ACTG1,PPP2R1B
Cardiac Hypertrophy Signaling	4.320	0.172	-2.333	PRKACB,MAP3K11,TGFBR1,MYL6,GNA11,MAP3K4,TGFBR2,PPP3CB,TGFB1,TGFB2,MRAS,PRKAR1B,MAP3K7,IRS2,PIK3R2,GNG5,GNG12,MAP3K2,ATM,PIK3C2A,RHOC,GRB2,GNA12,FGFR1,GNG2,ADCY6,GNAI1,PRKAR2A,MAPK12,GNG10,MYL9,GNAI2,ROCK1,GNAI3,PLCB4,MAPK14,RRAS2,IRS1,GNAO1,PRKACA
Thrombin Signaling	4.310	0.179	-0.577	RELA,MYL6,CAMK1D,GNA11,PPP1R12A,MRAS,AKT3,GATA6,ARHGEF2,IRS2,PIK3R2,GNG5,GNG12,ATM,EGFR,SRC,PIK3C2A,RHOC,GRB2,GNA12,FGFR1,GNG2,ADCY6,GNAI1,MAPK12,GNG10,MYL9,GNAI2,ROCK1,GNAI3,PLCB4,MAPK14,RRAS2,PRKCD,IRS1,GNAO1
RANK Signaling in Osteoclasts	4.190	0.220	-1.342	RELA,SRC,MAP3K11,PIK3C2A,GRB2,NFKBIE,FGFR1,TAB2,IKBKE,MAP3K4,MAPK12,MAPK14,PPP3CB,IRS1,MAP3K7,AKT3,IRS2,PIK3R2,CHUK,NFKBIB,MAP3K2,ATM
G Beta Gamma Signaling	4.180	0.211	-1.225	PRKACB,SRC,CAV2,GRB2,GNA12,GNG2,GNA11,PRKAR2A,GNAI1,GNG10,GNAI2,CACNA1F,GNAI3,PAK1,RRAS2,PRKCD,GNAO1,PRKACA,PRKAR1B,MRAS,AKT3,GNG5,GNG12,EGFR
Protein Kinase A Signaling	4.170	0.151	0.885	PRKACB,TGFBR1,MYL6,YWHAH,SMAD3,NTN1,TGFBR2,GYS1,TGFB1,GDE1,SMPDL3B,NFKBIB,PDE11A,GNG12,SMPDL3A,PPP1R14C,GNG2,ANAPC7,TTN,PTPRM,RAP1A,PDE1C,GNG10,MYL9,PRKCD,PRKACA,HIST1H1D,SIRPA,RAP1B,RELA,NFKBIE,AKAP2,NFAT5,PPP1R10,PPP1R12A,PPP3CB,PTPRJ,NGFR,H1FX,TGFB2,PRKAR1B,CHUK,GNG5,VASP,AKAP5,PTPRK,ATF1,PRKAR2A,ADCY6,GNAI1,HIST1H1B,GNAI2,ROCK1,GNAI3,PLCB4,HIST1H1E,CDC27,AKAP1
Aryl Hydrocarbon Receptor Signaling	4.160	0.199	-1.460	CDKN2A,RELA,NFIX,CCND1,GSTT1,ARNT,TGM2,RB1,ALDH2,CCND3,TGFB1,ALDH3A2,TGFB2,AHR,ATM,SRC,CCNE2,NQO1,CDK6,SLC35A2,AIP,CCND2,IL1B,NRIP1,NCOR2,CDKN1B,CDK2
fMLP Signaling in Neutrophils	4.130	0.205	-0.209	RELA,NFKBIE,NFAT5,PPP3CB,MRAS,IRS2,PIK3R2,NFKBIB,GNG5,GNG12,ATM,PIK3C2A,GRB2,FGFR1,GNG2,RAC1,GNAI1,GNG10,GNAI2,GNAI3,PLCB4,ARPC1A,RRAS2,IRS1,PRKCD
RhoA Signaling	4.130	0.205	-2.041	PFN1,MYL6,NRP2,CFL1,GNA12,ACTB,PIKFYVE,RAPGEF6,PIP4K2B,TTN,PLD1,LIMK1,MYL9,ROCK1,ARPC1A,CFL2,PPP1R12A,LPAR1,PLEKHG5,ARHGAP12,PIP5KL1,CDC42EP1,ARHGAP1,ACTG1,KTN1
Superpathway of Inositol Phosphate Compounds	4.110	0.170	0.480	PPP1R12C,MTMR9,NUDT12,PPFIA3,PPP2R5B,PIKFYVE,MDP1,PIP4K2B,NUDT14,NT5C,INPPL1,MTMR6,SET,IPMK,PTPRJ,PPP1R12A,PPP1R13B,PIP5KL1,IRS2,PIK3R2,ERBB2,RNGTT,EGFR,ATM,SRC,PIP4P1,PIK3C2A,GRB2,FGFR1,PTPRM,PLPP6,SYNJ2,TNS3,PLCB4,PDXP,PPP2R3A,IRS1,DUSP23,SIRPA
Actin Nucleation by ARP-WASP Complex	4.040	0.268	-1.941	ITGB1,PPP1R12C,RHOC,GRB2,GNA12,RAC1,ITGA5,NCK2,ROCK1,ITGA3,ARPC1A,RRAS2,PPP1R12A,MRAS,VASP
Role of NFAT in Cardiac Hypertrophy	3.990	0.171	-0.845	IL6ST,PRKACB,TGFBR1,CAMK1D,TGFBR2,CACNA1F,PPP3CB,TGFB1,TGFB2,MRAS,PRKAR1B,MAP3K7,AKT3,IRS2,PIK3R2,GNG5,GNG12,ATM,AKAP5,SRC,PIK3C2A,GRB2,FGFR1,GNG2,ADCY6,GNAI1,PRKAR2A,MAPK12,GNG10,GNAI2,GNAI3,PLCB4,MAPK14,RRAS2,PRKCD,IRS1,PRKACA
P2Y Purigenic Receptor Signaling Pathway	3.910	0.195	-1.633	PRKACB,RELA,ITGB3,MRAS,PRKAR1B,AKT3,IRS2,PIK3R2,GNG5,GNG12,ATM,PIK3C2A,GRB2,FGFR1,GNG2,ADCY6,GNAI1,PRKAR2A,GNG10,GNAI2,GNAI3,PLCB4,RRAS2,IRS1,PRKCD,PRKACA
3-phosphoinositide Biosynthesis	3.870	0.174	0.343	PPP1R12C,MTMR9,NUDT12,PPFIA3,PPP2R5B,MDP1,PIKFYVE,PIP4K2B,NUDT14,NT5C,MTMR6,SET,PTPRJ,PPP1R12A,PPP1R13B,PIP5KL1,IRS2,PIK3R2,ERBB2,RNGTT,EGFR,ATM,SRC,PIK3C2A,GRB2,FGFR1,PTPRM,PLPP6,TNS3,PDXP,PPP2R3A,IRS1,DUSP23,SIRPA
IL-17A Signaling in Airway Cells	3.810	0.231	-0.243	RELA,PIK3C2A,GRB2,NFKBIE,FGFR1,CCL20,IKBKE,MAPK12,MAPK14,TRAF3IP2,IRS1,MAP3K7,AKT3,IRS2,PIK3R2,CHUK,NFKBIB,ATM
IL-17A Signaling in Fibroblasts	3.780	0.314	NaN	RELA,TRAF3IP2,MAPK14,NFKBIE,LCN2,MAP3K7,IKBKE,CEBPB,CHUK,NFKBIB,MAPK12

Ingenuity Canonical Pathways	-log(p-value)	Ratio	z-score	Proteins
Role of PKR in Interferon Induction and Antiviral Response	3.740	0.293	NaN	RELA,TRAF3,MAPK14,CASP3,NFKBIE,TAB2,MAP3K7,BID,IKBKE,CHUK,NFKBIB,RNASEL
Ceramide Signaling	3.720	0.215	-0.243	RELA,PIK3C2A,CNKSR1,GRB2,FGFR1,PPP2R5B,SMPD2,KSR1,RRAS2,PPP2R3A,NGFR,IRS1,MRAS,AKT3,IRS2,PIK3R2,PPP2R1B,NSMAF,TNFRSF11B,ATM
Hepatic Fibrosis / Hepatic Stellate Cell Activation	3.700	0.175	NaN	RELA,FN1,CTGF,TGFBR1,MYL6,SMAD3,COL8A1,COL4A2,PDGFC,TGFBR2,COL5A1,COL6A1,TGFB1,NGFR,TGFB2,SERPINE1,TNFRSF11B,EGFR,TIMP2,VCAM1,COL4A1,COL12A1,FGFR1,IGFBP5,PDGFB,MYL9,COL1A1,IGFBP3,IL1B,COL11A1,A2M,COL3A1
Gap Junction Signaling	3.700	0.173	NaN	PRKACB,PPP3CB,MRAS,PRKAR1B,AKT3,TUBB4A,IRS2,PIK3R2,GUCY1B1,MAP3K2,ATM,EGFR,SRC,PIK3C2A,TJP1,GRB2,FGFR1,ACTB,TUBA4A,ADCY6,GNAI1,PRKAR2A,GJC1,GNAI2,GNAI3,PLCB4,RRAS2,LPAR1,PRKCD,IRS1,PRKACA,MAPK7,ACTG1
Ephrin A Signaling	3.680	0.250	NaN	ROCK1,PAK1,CFL1,CFL2,PIK3C2A,GRB2,NGFR,IRS1,FGFR1,RAC1,EPHA5,IRS2,PIK3R2,LIMK1,ATM
Antiproliferative Role of Somatostatin Receptor 2	3.680	0.233	-1.604	RAP1B,SRC,PIK3C2A,GRB2,FGFR1,GNG2,GNG10,RRAS2,IRS1,MRAS,IRS2,PIK3R2,CDKN1B,GNG5,GNG12,GUCY1B1,ATM
Interferon Signaling	3.660	0.306	2.530	RELA,IFIT3,IFIT1,MX1,IFI35,STAT2,IRF9,PSMB8,TAP1,IFITM1,ISG15
Role of p14/p19ARF in Tumor Suppression	3.630	0.286	0.302	CDKN2A,RB1,PIK3C2A,GRB2,NPM3,IRS1,FGFR1,RAC1,SENP3,IRS2,PIK3R2,ATM
Activation of IRF by Cytosolic Pattern Recognition Receptors	3.590	0.246	0.258	DHX58,RELA,TRAF3,NFKBIE,IKBKE,IRF9,TBK1,MAPK12,ISG15,TANK,STAT2,PIN1,CHUK,NFKBIB,IFIT2
Prostate Cancer Signaling	3.590	0.211	NaN	RELA,CCNE2,PIK3C2A,GRB2,FGFR1,NFKBIE,ABL1,CCND1,RB1,RRAS2,IRS1,MRAS,AKT3,IRS2,PIK3R2,CDKN1B,CHUK,NFKBIB,CDK2,ATM
Endothelin-1 Signaling	3.570	0.172	-1.061	GNA11,CASP4,HMOX1,MRAS,IRS2,PIK3R2,GUCY1B1,ATM,SRC,CASP3,PIK3C2A,GRB2,GNA12,FGFR1,PTGS1,ADCY6,GNAI1,MAPK12,PLD1,GNAI2,GNAI3,PLCB4,MAPK14,RRAS2,ARAF,PRKCD,IRS1,GNAO1,MAPK7,PAFAH1B1,CASP7,PAFAH1B3
Role of Osteoblasts, Osteoclasts and Chondrocytes in Rheumatoid Arthritis	3.500	0.162	NaN	RELA,NFKBIE,TAB2,SMAD5,ITGB3,ITGA3,WNT7A,NFAT5,PPP3CB,TGFB1,DKK3,NGFR,MAP3K7,AKT3,IRS2,PIK3R2,CHUK,NFKBIB,BMP1,TNFRSF11B,ATM,ITGB1,SRC,SPP1,PIK3C2A,GRB2,FGFR1,DVL1,ITGA5,IKBKE,MAPK12,COL1A1,MAPK14,IRS1,FZD6,IL1B,FZD7
PI3K Signaling in B Lymphocytes	3.480	0.190	-1.147	CD81,RELA,ATF3,C3,ATF1,NFKBIE,RAC1,ABL1,IKBKE,PLEKHA4,PLCB4,NFAT5,RRAS2,PPP3CB,CARD10,IRS1,LYN,MRAS,AKT3,IRS2,PLEKHA1,PIK3R2,CHUK,NFKBIB
Role of NFAT in Regulation of the Immune Response	3.440	0.171	-1.000	RELA,NFKBIE,HLA-B,GNA11,NFAT5,PPP3CB,MRAS,AKT3,IRS2,PIK3R2,CHUK,GNG5,NFKBIB,GNG12,ATM,AKAP5,PIK3C2A,GRB2,GNA12,FGFR1,GNG2,GNAI1,IKBKE,GNG10,GNAI2,GNAI3,PLCB4,RRAS2,IRS1,GNAO1,LYN
Gα12/13 Signaling	3.410	0.185	-1.877	RELA,SRC,MYL6,PIK3C2A,GRB2,FGFR1,GNA12,NFKBIE,CDH6,IKBKE,MAPK12,MYL9,ROCK1,CDH2,RRAS2,LPAR1,IRS1,MRAS,AKT3,IRS2,PIK3R2,CHUK,MAPK7,NFKBIB,ATM
Non-Small Cell Lung Cancer Signaling	3.390	0.221	-1.387	CDKN2A,PIK3C2A,GRB2,FGFR1,CDK6,ABL1,CCND1,RB1,RRAS2,IRS1,MRAS,AKT3,IRS2,ERBB2,PIK3R2,EGFR,ATM
Glioma Signaling	3.380	0.195	-1.000	CDKN2A,E2F4,PIK3C2A,CAMK1D,GRB2,FGFR1,CDK6,ABL1,CCND1,PDGFC,IGF2R,PDGFB,RB1,RRAS2,PRKCD,IRS1,MRAS,AKT3,IRS2,PIK3R2,EGFR,ATM
Reelin Signaling in Neurons	3.330	0.207	NaN	ITGB1,SRC,MAP3K11,PIK3C2A,GRB2,FGFR1,ITGA5,MAPK12,ITGB3,ITGA3,IRS1,LYN,ARHGEF2,IRS2,ITGA1,PIK3R2,PAFAH1B1,PAFAH1B3,ATM
PPAR Signaling	3.330	0.207	1.606	RELA,GRB2,NFKBIE,IKBKE,PDGFC,PDGFB,AIP,RRAS2,NGFR,INS,MAP3K7,MRAS,IL1B,NRIP1,NCOR2,CHUK,NFKBIB,MAP4K4,TNFRSF11B
Type II Diabetes Mellitus Signaling	3.330	0.178	-1.528	RELA,ACSL3,PIK3C2A,GRB2,FGFR1,NFKBIE,IKBKE,CEBPB,MAPK12,SMPD2,ADIPOR1,SLC27A4,CACNA1F,NGFR,PRKCD,IRS1,INS,MAP3K7,ACSL4,AKT3,IRS2,PIK3R2,CHUK,NFKBIB,NSMAF,TNFRSF11B,ATM
CD27 Signaling in Lymphocytes	3.260	0.250	-0.905	RELA,MAP3K11,CASP3,CD70,NFKBIE,MAP3K7,BID,IKBKE,MAP3K4,CHUK,NFKBIB,MAPK12,MAP3K2
HIPPO signaling	3.260	0.209	0.000	DLG1,PPP1R14C,YAP1,YWHAH,SMAD3,WWTR1,PPP2R5B,NF2,FRMD6,SMAD5,TP53BP2,PPP1R10,PPP1R12A,PPP2R3A,AJUBA,SCRIB,PATJ,PPP2R1B
Regulation of IL-2 Expression in Activated and Anergic T Lymphocytes	3.250	0.215	NaN	RELA,TGFBR1,GRB2,NFKBIE,SMAD3,RAC1,IKBKE,MAPK12,TGFBR2,NFAT5,RRAS2,PPP3CB,TGFB1,MRAS,TGFB2,CHUK,NFKBIB

Ingenuity Canonical Pathways	-log(p-value)	Ratio	z-score	Proteins
Role of Macrophages, Fibroblasts and Endothelial Cells in Rheumatoid Arthritis	3.240	0.149	NaN	IL6ST,RELA,TRAF3,FN1,NFKBIE,CCND1,PDGFC,CEBPG,TRAF3IP2,WNT7A,NFAT5,PPP3CB,DKK3,TGFB1,NGFR,MRAS,MAP3K7,AKT3,IRS2,PIK3R2,CHUK,NFKBIB,TNFRSF11B,ATM,SRC,VCAM1,PIK3C2A,GRB2,FGFR1,DVL1,DAAM1,RAC1,IKBKE,CEBPB,PDGFB,ROCK1,PLCB4,RRAS2,MAPK14,PRKCD,IRS1,GNAO1,FZD6,IL1B,FZD7
HMGB1 Signaling	3.220	0.183	-1.043	RELA,VCAM1,PIK3C2A,GRB2,RHOC,FGFR1,RAC1,MAPK12,HMGB1,MAPK14,RRAS2,TGFB1,NGFR,IRS1,MRAS,TGFB2,AKT3,IL1B,IRS2,PIK3R2,SERPINE1,TNFRSF11B,PLAT,ATM
p70S6K Signaling	3.220	0.183	-0.209	SRC,YWHAH,PIK3C2A,GRB2,FGFR1,PPP2R5B,GNAI1,PLD1,GNAI2,GNAI3,PLCB4,RRAS2,PPP2R3A,PRKCD,IRS1,LYN,MRAS,AKT3,IRS2,PIK3R2,EEF2K,PPP2R1B,EGFR,ATM
Cell Cycle: G1/S Checkpoint Regulation	3.200	0.227	0.832	CDKN2A,CCNE2,E2F4,SMAD3,CDK6,ABL1,CCND1,RB1,CCND2,CCND3,TGFB1,TGFB2,CDKN1B,CDK2,ATM
Cyclins and Cell Cycle Regulation	3.190	0.212	-0.258	CDKN2A,CCNE2,E2F4,PPP2R5B,CDK6,ABL1,CCND1,RB1,CCND2,CCND3,TGFB1,PPP2R3A,TGFB2,CDKN1B,PPP2R1B,CDK2,ATM
Cardiac β-adrenergic Signaling	3.160	0.179	-0.943	PRKACB,PPP2R5B,ATP2A2,AKAP2,PPP1R10,PPP1R12A,GDE1,PRKAR1B,MRAS,SMPDL3B,PDE1A,GNG5,GNG12,SMPDL3A,AKAP5,PPP1R14C,GNG2,ADCY6,PRKAR2A,PDE1C,GNG10,PPP2R3A,PRKACA,PPP2R1B,AKAP1
Coagulation System	3.120	0.286	1.265	F5,F13A1,PLAUR,SERPINA1,FGB,SERPINE1,TFPI,A2M,SERPINF2,PLAT
SAPK/JNK Signaling	3.110	0.194	-1.606	RAC2,MAP3K11,PIK3C2A,GRB2,FGFR1,GNA12,GNG2,RAC1,MAP3K4,MAPK12,RRAS2,IRS1,MAP3K7,MRAS,IRS2,PIK3R2,GNG5,MAP4K4,MAP3K2,ATM
TNFR1 Signaling	3.050	0.250	-1.155	TANK,RELA,PAK4,PAK1,CASP3,NFKBIE,PAK2,BID,IKBKE,CHUK,NFKBIB,CASP7
GADD45 Signaling	3.040	0.368	NaN	CCNE2,CCND2,CCND3,MAP3K4,CCND1,CDK2,ATM
Gαq Signaling	3.020	0.170	-1.460	RELA,NFKBIE,GNA11,HMOX1,GYS1,PPP3CB,MRAS,AKT3,IRS2,CHUK,PIK3R2,NFKBIB,GNG5,GNG12,ATM,PIK3C2A,GRB2,RHOC,FGFR1,GNG2,IKBKE,PLD1,GNG10,ROCK1,PLCB4,IRS1,PRKCD
Melanoma Signaling	3.010	0.236	-1.155	CDKN2A,RB1,RRAS2,PIK3C2A,GRB2,IRS1,FGFR1,MRAS,AKT3,IRS2,PIK3R2,CCND1,ATM
Mouse Embryonic Stem Cell Pluripotency	3.000	0.190	-0.447	IL6ST,PIK3C2A,GRB2,FGFR1,DVL1,SMAD5,MAPK12,LIFR,DVL2,MAPK14,RRAS2,IRS1,FZD6,MAP3K7,MRAS,AKT3,IRS2,PIK3R2,ATM,FZD7
UVA-Induced MAPK Signaling	3.000	0.190	-0.905	CASP3,PIK3C2A,GRB2,PARP10,FGFR1,RPS6KA5,PARP12,MAPK12,SMPD2,TNKS,PLCB4,MAPK14,RRAS2,IRS1,MRAS,IRS2,PIK3R2,PARP14,EGFR,ATM
Dopamine Receptor Signaling	2.990	0.211	-0.816	PRKACB,PPP1R14C,PPP2R5B,PRKAR2A,ADCY6,IL4I1,PPP1R12A,PPP1R10,PPP2R3A,PTS,PRKACA,NCS1,PRKAR1B,SPR,PPP2R1B,MAOA
Androgen Signaling	2.990	0.176	-1.508	PRKACB,SRC,RELA,SMAD3,GNA12,GNG2,GNA11,PRKAR2A,GNAI1,GTF2A1,CCND1,GNG10,GNAI2,CACNA1F,GNAI3,TGFB1I1,ERCC3,PRKCD,GNAO1,PRKACA,PRKAR1B,MRAS,GNG5,GNG12
IL-12 Signaling and Production in Macrophages	2.980	0.174	NaN	RELA,PIK3C2A,GRB2,FGFR1,RAB7A,IKBKE,CEBPB,MAPK12,APOL1,JMJD6,ALB,MAPK14,TGFB1,PRKCD,IRS1,TGFB2,AKT3,SERPINA1,IRS2,PIK3R2,CHUK,NFKBIB,CLU,ATM,APOC3
LXR/RXR Activation	2.960	0.182	3.130	RELA,C3,AHSG,SERPINF1,SAA2,A1BG,SERPINF2,APOL1,ALB,LDLR,TF,NGFR,SAA1,IL1B,SERPINA1,PLTP,NCOR2,GC,HMGCR,CLU,APOC3,TNFRSF11B
Renin-Angiotensin Signaling	2.960	0.182	-2.400	PRKACB,RELA,PAK4,PIK3C2A,GRB2,FGFR1,PRKAR2A,RAC1,ADCY6,MAPK12,PAK1,MAPK14,RRAS2,PRKCD,IRS1,PAK2,PRKACA,PRKAR1B,MRAS,IRS2,PIK3R2,ATM
IGF-1 Signaling	2.950	0.189	-1.604	PRKACB,CTGF,YWHAH,PIK3C2A,GRB2,FGFR1,PRKAR2A,IGFBP5,IGFBP7,RRAS2,IRS1,PRKACA,IGFBP3,PRKAR1B,MRAS,AKT3,IRS2,PIK3R2,CYR61,ATM
Insulin Receptor Signaling	2.940	0.175	0.426	PRKACB,TSC1,PPP1R14C,PIK3C2A,GRB2,FGFR1,PRKAR2A,INPPL1,VAMP2,EIF4EBP1,SYNJ2,GYS1,RRAS2,PPP1R10,PPP1R12A,IRS1,INS,PRKACA,PRKAR1B,MRAS,AKT3,IRS2,PIK3R2,ATM
Myc Mediated Apoptosis Signaling	2.920	0.214	NaN	CDKN2A,CASP3,PIK3C2A,YWHAH,GRB2,FGFR1,MAPK12,RRAS2,IRS1,MRAS,BID,AKT3,IRS2,PIK3R2,ATM
Glioma Invasiveness Signaling	2.920	0.214	-2.324	PIK3C2A,GRB2,RHOC,FGFR1,PLAUR,ITGB3,RRAS2,IRS1,ITGAV,MRAS,IRS2,PIK3R2,ITGB5,ATM,TIMP2
14-3-3-mediated Signaling	2.900	0.177	-1.147	TSC1,SRC,YWHAH,YAP1,PIK3C2A,GRB2,FGFR1,EDC3,TUBA4A,MAPK12,PLCB4,RRAS2,PRKCD,IRS1,MRAS,AKT3,TUBB4A,IRS2,PIK3R2,CDKN1B,SNCA,AKT1S1,ATM

Ingenuity Canonical Pathways	-log(p-value)	Ratio	z-score	Proteins
Opioid Signaling Pathway	2.890	0.152	-0.522	RAP1B,PRKACB,RAC2,CAMK1D,SLC12A5,CACNA1F,AP1G2,ARRB1,PPP3CB,RGS10,MRAS,PRKAR1B,AKT3,GNG5,SIGMAR1,SRC,RGS20,GNG2,RAC1,ADCY6,GNAI1,PRKAR2A,RPS6KA5,MAPK12,RAP1A,PDE1C,GNAI2,GNAI3,ARRB2,RRAS2,PRKCD,GNAO1,LYN,PRKACA,CDKN1B,MAPK7
Xenobiotic Metabolism Signaling	2.830	0.147	NaN	RELA,MAP3K11,CAMK1D,CHST7,PPP2R5B,MAP3K4,CES2,GSTT1,ARNT,HMOX1,ALDH2,ALDH3A2,MRAS,MAP3K7,IRS2,PIK3R2,AHR,MAP3K2,ATM,ABCB1,PIK3C2A,GRB2,FGFR1,NQO1,MAPK12,IL4I1,AIP,RRAS2,MAPK14,PPP2R3A,PRKCD,IRS1,IL1B,NCOR2,MAPK7,NRIP1,ABCC3,NDST1,PPP2R1B,MAOA
Sphingosine-1-phosphate Signaling	2.820	0.177	-1.279	CASP3,PIK3C2A,GRB2,RHOC,FGFR1,GNA12,RAC1,GNAI1,ADCY6,CASP4,PDGFC,SMPD2,PDGFB,GNAI2,GNAI3,PLCB4,IRS1,AKT3,IRS2,PIK3R2,CASP7,ATM
4-1BB Signaling in T Lymphocytes	2.800	0.281	-0.378	RELA,TNFRSF9,MAPK14,NFKBIE,TNFSF9,IKBKE,CHUK,NFKBIB,MAPK12
Estrogen-Dependent Breast Cancer Signaling	2.800	0.203	-1.604	HSD17B10,RELA,SRC,PIK3C2A,GRB2,FGFR1,CCND1,RRAS2,IRS1,MRAS,AKT3,HSD17B12,IRS2,PIK3R2,EGFR,ATM
Adrenomedullin signaling pathway	2.800	0.159	-1.890	ADM,PRKACB,RELA,GNA11,ARNT,MRAS,PRKAR1B,AKT3,IRS2,PIK3R2,GUCY1B1,ATM,C3,CASP3,PIK3C2A,GRB2,FGFR1,ADCY6,PRKAR2A,CEBPB,MAPK12,TTN,PLCB4,MAPK14,RRAS2,ARAF,IRS1,PRKACA,IL1B,MAPK7
Glucocorticoid Receptor Signaling	2.790	0.140	NaN	PRKACB,RELA,KRT34,TGFBR1,YWHAH,NFKBIE,SMAD3,GTF2A1,KRT36,TGFBR2,GTF2A2,HMGB1,NFAT5,PPP3CB,BAG1,TGFB1,TGFB2,MAP3K7,MRAS,AKT3,IRS2,PIK3R2,CHUK,SERPINE1,NFKBIB,ATM,VCAM1,CDKN1C,PIK3C2A,GRB2,FGFR1,ACTB,RAC1,IKBKE,KRT80,CEBPB,MAPK12,HSPA2,RRAS2,MAPK14,ERCC3,IRS1,PRKACA,IL1B,NRIP1,NCOR2,A2M
NRF2-mediated Oxidative Stress Response	2.760	0.158	-1.069	DNAJB4,DNAJB2,GSTT1,HMOX1,AKR1A1,SOD2,MRAS,MAP3K7,IRS2,PIK3R2,TXN,ATM,PIK3C2A,GRB2,ACTB,FGFR1,NQO1,SLC35A2,JUNB,MAFF,MAPK14,RRAS2,ERP29,PRKCD,IRS1,MAPK7,DNAJB6,ACTG1,ENC1,HACD3
Docosahexaenoic Acid (DHA) Signaling	2.730	0.231	NaN	CASP3,PIK3C2A,GRB2,IRS1,FGFR1,SERPINF1,BID,IL1B,AKT3,IRS2,PIK3R2,ATM
3-phosphoinositide Degradation	2.680	0.166	1.000	PPP1R12C,MTMR9,PPP2R5B,PPFIA3,NUDT12,MDP1,PIKFYVE,NUDT14,INPPL1,NT5C,MTMR6,SET,PPP1R12A,PTPRJ,PPP1R13B,RNGTT,PIP4P1,PTPRM,SYNJ2,PLPP6,TNS3,PPP2R3A,PDXP,DUSP23,SIRPA
Lymphotoxin β Receptor Signaling	2.650	0.209	-0.277	RELA,TRAF3,VCAM1,CASP3,PIK3C2A,GRB2,FGFR1,IKBKE,IRS1,AKT3,IRS2,CHUK,PIK3R2,ATM
ErbB Signaling	2.630	0.186	-1.213	PAK4,PIK3C2A,GRB2,FGFR1,MAPK12,NCK2,PAK1,MAPK14,RRAS2,PRKCD,IRS1,PAK2,MRAS,IRS2,ERBB2,PIK3R2,EGFR,ATM
TWEAK Signaling	2.600	0.265	1.000	RELA,TRAF3,CASP3,NFKBIE,BID,IKBKE,CHUK,NFKBIB,CASP7
D-myo-inositol (1,4,5,6)-Tetrakisphosphate Biosynthesis	2.590	0.168	1.043	PPP1R12C,MTMR9,NUDT12,PPFIA3,PPP2R5B,MDP1,PIKFYVE,NUDT14,NT5C,PTPRM,MTMR6,PLPP6,SET,TNS3,IPMK,PPP1R12A,PTPRJ,PPP2R3A,PDXP,PPP1R13B,DUSP23,RNGTT,SIRPA
D-myo-inositol (3,4,5,6)-tetrakisphosphate Biosynthesis	2.590	0.168	1.043	PPP1R12C,MTMR9,NUDT12,PPFIA3,PPP2R5B,MDP1,PIKFYVE,NUDT14,NT5C,PTPRM,MTMR6,PLPP6,SET,TNS3,IPMK,PPP1R12A,PTPRJ,PPP2R3A,PDXP,PPP1R13B,DUSP23,RNGTT,SIRPA
Granzyme A Signaling	2.560	0.353	NaN	HIST1H1B,SET,HIST1H1E,H1FX,HIST1H1D,HMGB2
γ-linolenate Biosynthesis II (Animals)	2.560	0.353	0.000	ACSL3,FADS2,ACSL4,CYB5A,CYB5R3,SLC27A4
D-myo-inositol-5-phosphate Metabolism	2.520	0.161	1.000	PPP1R12C,MTMR9,PPP2R5B,PPFIA3,NUDT12,MDP1,PIKFYVE,PIP4K2B,NUDT14,NT5C,MTMR6,SET,PPP1R12A,PTPRJ,PPP1R13B,RNGTT,PIP4P1,PTPRM,PLPP6,PLCB4,TNS3,PPP2R3A,PDXP,DUSP23,SIRPA
Pyrimidine Deoxyribonucleotides De Novo Biosynthesis I	2.500	0.304	1.890	TYMS,AK5,CMPK2,RRM2,APOBEC3G,NME7,RRM1
VEGF Signaling	2.480	0.180	-2.000	SRC,PIK3C2A,GRB2,ACTB,FGFR1,PDGFC,ELAVL1,ARNT,ROCK1,RRAS2,IRS1,MRAS,AKT3,IRS2,PIK3R2,VCL,ACTG1,ATM
Human Embryonic Stem Cell Pluripotency	2.470	0.164	NaN	TGFBR1,PIK3C2A,GRB2,SMAD3,FGFR1,DVL1,SMAD5,PDGFC,INHBA,PDGFB,TGFBR2,WNT7A,TGFB1,IRS1,FZD6,MRAS,TGFB2,AKT3,IRS2,PIK3R2,BMP1,ATM,FZD7
α-Adrenergic Signaling	2.460	0.188	0.000	PRKACB,GNG2,PRKAR2A,GNAI1,ADCY6,GNG10,GNAI2,GNAI3,RRAS2,GYS1,PRKCD,PRKACA,PRKAR1B,MRAS,GNG5,GNG12
IL-17 Signaling	2.460	0.188	NaN	RELA,PIK3C2A,GRB2,FGFR1,CEBPB,MAPK12,MAPK14,TRAF3IP2,RRAS2,IRS1,MAP3K7,MRAS,AKT3,IRS2,PIK3R2,ATM

Ingenuity Canonical Pathways	-log(p-value)	Ratio	z-score	Proteins
FXR/RXR Activation	2.420	0.168	NaN	SLC4A2,C3,SDC1,AHSG,SERPINF1,SAA2,MAPK12,A1BG,SERPINF2,APOL1,ALB,TF,SAA1,INS,AKT3,IL1B,SERPINA1,PLTP,GC,CLU,APOC3
Cholesterol Biosynthesis I	2.390	0.385	1.342	SQLE,NSDHL,MSMO1,LSS,LBR
Cholesterol Biosynthesis II (via 24,25-dihydrolanosterol)	2.390	0.385	1.342	SQLE,NSDHL,MSMO1,LSS,LBR
Cholesterol Biosynthesis III (via Desmosterol)	2.390	0.385	1.342	SQLE,NSDHL,MSMO1,LSS,LBR
Endometrial Cancer Signaling	2.390	0.203	-1.155	RRAS2,PIK3C2A,GRB2,IRS1,FGFR1,MRAS,CTNNA1,AKT3,IRS2,ERBB2,PIK3R2,CCND1,ATM
Tumoricidal Function of Hepatic Natural Killer Cells	2.380	0.292	0.447	M6PR,ENDOG,SERPINB9,CASP3,BID,DFFB,CASP7
CDP-diacylglycerol Biosynthesis I	2.380	0.292	1.890	AGPAT4,LPCAT2,LPCAT1,AGPAT2,GPAT3,AGPAT3,CDS2
G Protein Signaling Mediated by Tubby	2.290	0.258	NaN	PLCB4,INS,GNG2,MRAS,ABL1,GNG5,GNG12,GNG10
UVB-Induced MAPK Signaling	2.270	0.197	-0.277	PIK3C2A,GRB2,FGFR1,RPS6KA5,MAPK12,EIF4EBP1,MAPK14,IRS1,PRKCD,IRS2,PIK3R2,EGFR,ATM
Erythropoietin Signaling	2.270	0.185	NaN	RELA,SRC,RRAS2,PIK3C2A,GRB2,PRKCD,NFKBIE,IRS1,FGFR1,MRAS,AKT3,IRS2,PIK3R2,NFKBIB,ATM
STAT3 Pathway	2.250	0.175	-1.213	SRC,TGFBR1,MAP3K11,FGFR1,RAC1,MAPK12,IGF2R,PDGFB,TGFBR2,MAPK14,RRAS2,TGFB1,NGFR,MRAS,TGFB2,IL1B,EGFR
PKCθ Signaling in T Lymphocytes	2.250	0.156	-2.294	RELA,RAC2,MAP3K11,PIK3C2A,GRB2,FGFR1,NFKBIE,HLA-B,RAC1,IKBKE,MAP3K4,CACNA1F,NFAT5,RRAS2,PPP3CB,IRS1,MAP3K7,MRAS,IRS2,PIK3R2,CHUK,NFKBIB,MAP3K2,ATM
BMP signaling pathway	2.240	0.189	-1.941	PRKACB,RELA,MAGED1,GRB2,PRKAR2A,SMAD5,MAPK12,MAPK14,RRAS2,PRKACA,MAP3K7,MRAS,PRKAR1B,BMP1
Induction of Apoptosis by HIV1	2.190	0.200	1.732	RELA,BBC3,CASP3,NFKBIE,NGFR,BID,DFFB,IKBKE,CHUK,NFKBIB,MAPK12,TNFRSF11B
CNTF Signaling	2.190	0.200	-0.577	IL6ST,LIFR,RRAS2,PIK3C2A,GRB2,IRS1,FGFR1,MRAS,IRS2,RPS6KA5,PIK3R2,ATM
PCP pathway	2.190	0.200	0.302	ROCK1,DVL2,WNT7A,PFN1,VANGL1,DVL1,DAAM1,RAC1,FZD6,JUNB,MAPK12,FZD7
Phosphatidylglycerol Biosynthesis II (Non-plastidic)	2.170	0.269	1.890	AGPAT4,LPCAT2,LPCAT1,AGPAT2,GPAT3,AGPAT3,CDS2
April Mediated Signaling	2.170	0.231	0.333	RELA,TRAF3,NFAT5,MAPK14,NFKBIE,IKBKE,CHUK,NFKBIB,MAPK12
FLT3 Signaling in Hematopoietic Progenitor Cells	2.170	0.181	-0.775	PIK3C2A,GRB2,FGFR1,RPS6KA5,MAPK12,EIF4EBP1,MAPK14,RRAS2,IRS1,MRAS,AKT3,IRS2,STAT2,PIK3R2,ATM
Chemokine Signaling	2.160	0.191	-0.277	SRC,CFL1,CAMK1D,GNAI1,MAPK12,LIMK1,GNAI2,GNAI3,PLCB4,MAPK14,RRAS2,PPP1R12A,MRAS
Phospholipase C Signaling	2.160	0.140	-1.732	RAP1B,PEBP1,RELA,MYL6,ARHGEF7,TGM2,HMOX1,ITGA3,NFAT5,AHNAK,PPP1R12A,PPP3CB,MRAS,ARHGEF2,GNG5,GNG12,ITGB1,SRC,GRB2,RHOC,GNG2,RAC1,ADCY6,ITGA5,RAP1A,PLD1,GNG10,MYL9,MARCKS,PLCB4,RRAS2,PRKCD,LYN
IL-15 Signaling	2.130	0.184	NaN	RELA,VCAM1,RRAS2,MAPK14,PIK3C2A,GRB2,IRS1,FGFR1,MRAS,AKT3,IRS2,PIK3R2,MAPK12,ATM
Role of PI3K/AKT Signaling in the Pathogenesis of Influenza	2.130	0.184	0.000	GNAI2,RELA,GNAI3,PIK3C2A,GRB2,NFKBIE,IRS1,FGFR1,GNAI1,AKT3,IRS2,PIK3R2,NFKBIB,ATM
HIF1α Signaling	2.120	0.164	NaN	MMP7,PIK3C2A,GRB2,FGFR1,MAPK12,PDGFC,ARNT,P4HTM,MAPK14,RRAS2,CUL2,IRS1,MRAS,AKT3,IRS2,MAPK7,PIK3R2,VHL,ATM
Role of IL-17A in Arthritis	2.100	0.188	NaN	RELA,MAPK14,PIK3C2A,GRB2,NFKBIE,IRS1,FGFR1,CCL20,IRS2,PIK3R2,NFKBIB,MAPK12,ATM
Cysteine Biosynthesis/Homocysteine Degradation	2.090	1.000	NaN	CBS/CBSL,CTH
Huntington's Disease Signaling	2.080	0.138	0.000	GNA11,CASP4,TGM2,AKT3,TCERG1,IRS2,PIK3R2,GOSR1,GNG5,BET1L,GNG12,NAPB,EGFR,ATM,CAPN5,ATP5PF,CASP3,YKT6,PIK3C2A,GRB2,RCOR3,GLS,FGFR1,GNG2,STX1A,HSPA2,GNG10,PLCB4,IRS1,PRKCD,NCOR2,GOSR2,SNCA,CASP7
Leptin Signaling in Obesity	2.070	0.176	-0.333	PRKACB,PIK3C2A,GRB2,FGFR1,PRKAR2A,ADCY6,PLCB4,IRS1,INS,PRKACA,PRKAR1B,AKT3,IRS2,PIK3R2,ATM
Fcγ Receptor-mediated Phagocytosis in Macrophages and Monocytes	2.070	0.172	-1.500	SRC,RAC2,ACTB,RAC1,TLN1,PLD1,NCK2,HMOX1,PAK1,ARPC1A,PRKCD,LYN,AKT3,PIK3R2,ACTG1,VASP
iCOS-iCOSL Signaling in T Helper Cells	2.040	0.161	-0.775	RELA,PIK3C2A,GRB2,FGFR1,NFKBIE,HLA-B,RAC1,IKBKE,PLEKHA4,NFAT5,PPP3CB,IRS1,AKT3,IRS2,PLEKHA1,CHUK,PIK3R2,NFKBIB,ATM
B Cell Activating Factor Signaling	2.030	0.220	-0.378	RELA,TRAF3,NFAT5,MAPK14,NFKBIE,IKBKE,CHUK,NFKBIB,MAPK12

Ingenuity Canonical Pathways	-log(p-value)	Ratio	z-score	Proteins
Mechanisms of Viral Exit from Host Cells	2.030	0.220	NaN	SH3GL1,SNF8,CHMP2A,ACTB,PRKCD,VPS36,CHMP4C,ACTG1,VPS4A
IL-4 Signaling	2.030	0.174	NaN	PIK3C2A,GRB2,FGFR1,HLA-B,INPPL1,HMGA1,SYNJ2,NFAT5,RRAS2,IRS1,MRAS,AKT3,IRS2,PIK3R2,ATM
IL-7 Signaling Pathway	2.030	0.174	-1.291	MAPK14,CCND3,PIK3C2A,GRB2,IRS1,FGFR1,LYN,AKT3,IRS2,PIK3R2,CDKN1B,MAPK12,CCND1,CDK2,ATM
PXR/RXR Activation	2.020	0.190	NaN	PRKACB,RELA,ABCB1,CPT1A,ALDH3A2,INS,PRKAR2A,PRKACA,PRKAR1B,AKT3,CES2,ABCC3
CD28 Signaling in T Helper Cells	2.020	0.157	-0.728	RELA,PIK3C2A,GRB2,FGFR1,NFKBIE,HLA-B,RAC1,IKBKE,MAPK12,PAK1,ARPC1A,NFAT5,PPP3CB,IRS1,AKT3,IRS2,PIK3R2,CHUK,NFKBIB,ATM
Regulation of the Epithelial-Mesenchymal Transition Pathway	2.020	0.144	NaN	RELA,TGFBR1,PIK3C2A,GRB2,FGFR1,SMAD3,DVL1,PARD6B,PSEN2,TGFBR2,DVL2,CDH2,RRAS2,WNT7A,ARAF,TGFB1,IRS1,TGFB2,MRAS,FZD6,AKT3,IRS2,PIK3R2,HMGA2,ATM,EGFR,FZD7
TGF-β Signaling	1.980	0.172	-1.807	INHA,TGFBR1,GRB2,SMAD3,SMAD5,MAPK12,INHBA,TGFBR2,MAPK14,RRAS2,TGFB1,MAP3K7,MRAS,TGFB2,SERPINE1
Role of NANOG in Mammalian Embryonic Stem Cell Pluripotency	1.970	0.158	-0.577	IL6ST,PIK3C2A,GRB2,FGFR1,DVL1,SMAD5,LIFR,WNT7A,RRAS2,IRS1,FZD6,MRAS,AKT3,GATA6,IRS2,PIK3R2,BMP1,ATM,FZD7
Gαi Signaling	1.970	0.158	0.243	PRKACB,SRC,GRB2,GNG2,PRKAR2A,GNAI1,ADCY6,RAP1A,GNG10,GNAI2,GNAI3,RRAS2,LPAR1,RGS10,PRKACA,PRKAR1B,MRAS,GNG5,GNG12
Granzyme B Signaling	1.960	0.312	1.342	ENDOG,NUMA1,CASP3,BID,DFFB
Role of RIG1-like Receptors in Antiviral Innate Immunity	1.960	0.214	-1.134	DHX58,TANK,RELA,TRAF3,NFKBIE,IKBKE,TBK1,CHUK,NFKBIB
Regulation of eIF4 and p70S6K Signaling	1.960	0.149	-1.500	ITGB1,PABPC1,EIF4EBP2,PIK3C2A,GRB2,FGFR1,PPP2R5B,ITGA5,AGO2,MAPK12,EIF4EBP1,ITGA3,MAPK14,RRAS2,PPP2R3A,IRS1,AGO3,MRAS,AKT3,IRS2,PIK3R2,PPP2R1B,ATM
Cdc42 Signaling	1.950	0.155	-2.982	ITGB1,SRC,PAK4,MAP3K11,CFL1,MYL6,DIAPH3,HLA-B,ITGA5,IQGAP1,MAPK12,LIMK1,MYL9,ITGA3,PAK1,ARPC1A,MAPK14,PPP1R12A,CFL2,PAK2
Arginine Biosynthesis IV	1.930	0.500	NaN	OAT,GLUD1,ASL
Proline Biosynthesis II (from Arginine)	1.930	0.500	NaN	OAT,PYCR2,PYCR1
Arginine Degradation VI (Arginase 2 Pathway)	1.930	0.500	NaN	OAT,PYCR2,PYCR1
Zymosterol Biosynthesis	1.930	0.500	NaN	NSDHL,MSMO1,LBR
Phagosome Maturation	1.930	0.152	NaN	M6PR,ATP6V0C,YKT6,TCIRG1,HLA-B,TUBA4A,RAB7A,PIKFYVE,TAP1,ATP6AP1,DYNLRB1,CTSA,CTSL,CTSS,LAMP1,TUBB4A,VPS37A,GOSR1,GOSR2,BET1L,NAPB
Amyloid Processing	1.910	0.200	-1.000	PRKACB,CAPN5,MAPK14,PRKAR2A,PRKACA,PRKAR1B,AKT3,PSEN2,BACE2,MAPK12
Netrin Signaling	1.910	0.185	NaN	PRKACB,NCK2,CACNA1F,RAC2,NFAT5,PPP3CB,PRKAR2A,RAC1,PRKACA,PRKAR1B,NTN1,ENAH
TNFR2 Signaling	1.900	0.241	-1.134	TANK,RELA,NFKBIE,IKBKE,TBK1,CHUK,NFKBIB
GM-CSF Signaling	1.900	0.178	-1.155	PIK3C2A,GRB2,FGFR1,CCND1,RRAS2,PPP3CB,IRS1,LYN,MRAS,AKT3,IRS2,PIK3R2,ATM
UDP-N-acetyl-D-galactosamine Biosynthesis II	1.890	0.364	2.000	GPI,HK2,GNPDA2,UAP1
UVC-Induced MAPK Signaling	1.890	0.209	-0.333	SRC,RRAS2,MAPK14,ARAF,PRKCD,MRAS,MAPK12,SMPD2,EGFR
Apoptosis Signaling	1.890	0.169	0.535	CAPN5,ENDOG,RELA,CASP3,NFKBIE,IKBKE,ROCK1,RRAS2,MRAS,BID,DFFB,CHUK,NFKBIB,MAP4K4,CASP7
Remodeling of Epithelial Adherens Junctions	1.860	0.182	-0.447	SRC,ARPC1A,ACTB,TUBA4A,RAB7A,CTNNA1,TUBB4A,VCL,IQGAP1,ACTG1,MAPRE3,CTNND1
Melanocyte Development and Pigmentation Signaling	1.860	0.163	-2.000	PRKACB,SRC,PIK3C2A,GRB2,FGFR1,PRKAR2A,ADCY6,RPS6KA5,RRAS2,IRS1,PRKACA,PRKAR1B,MRAS,IRS2,PIK3R2,ATM
PDGF Signaling	1.850	0.167	-2.324	SYNJ2,SRC,RRAS2,PIK3C2A,GRB2,IRS1,FGFR1,ABL1,MRAS,INPPL1,IRS2,PIK3R2,PDGFC,PDGFB,ATM
Hepatic Cholestasis	1.840	0.146	NaN	PRKACB,RELA,ABCB1,SLC4A2,NFKBIE,PRKAR2A,ADCY6,IKBKE,MAP3K4,MAPK12,TGFB1,NGFR,PRKCD,INS,PRKACA,PRKAR1B,MAP3K7,TGFB2,IL1B,CHUK,ABCC3,NFKBIB,TNFRSF11B

Ingenuity Canonical Pathways	-log(p-value)	Ratio	z-score	Proteins
Triacylglycerol Biosynthesis	1.820	0.205	2.333	AGPAT4,PORCN,LPCAT2,LPCAT1,PLPP3,AGPAT2,GPAT3,PLPP2,AGPAT3
Cell Cycle Regulation by BTG Family Proteins	1.810	0.216	-0.447	RB1,E2F4,CCNE2,PPP2R3A,PPP2R5B,PPP2R1B,CCND1,CDK2
IL-3 Signaling	1.800	0.169	-1.069	PIK3C2A,GRB2,FGFR1,RAC1,PAK1,RRAS2,PPP3CB,IRS1,PRKCD,MRAS,AKT3,IRS2,PIK3R2,ATM
Telomerase Signaling	1.790	0.157	-0.258	PIK3C2A,GRB2,FGFR1,PPP2R5B,ABL1,RB1,RRAS2,PPP2R3A,IRS1,MRAS,AKT3,IRS2,PIK3R2,PPP2R1B,TERF1,EGFR,ATM
Nitric Oxide Signaling in the Cardiovascular System	1.790	0.157	-1.698	PRKACB,PIK3C2A,GRB2,FGFR1,PRKAR2A,ATP2A2,PDGFC,PDE1C,IRS1,PRKCD,PRKACA,PRKAR1B,AKT3,IRS2,PIK3R2,GUCY1B1,ATM
Acute Myeloid Leukemia Signaling	1.770	0.163	-2.138	RELA,PIK3C2A,GRB2,FGFR1,CCND1,EIF4EBP1,RRAS2,ARAF,IRS1,MRAS,AKT3,IRS2,JUP,PIK3R2,ATM
Wnt/β-catenin Signaling	1.770	0.142	0.426	SOX4,CDKN2A,SRC,MMP7,TGFBR1,DVL1,PPP2R5B,CCND1,TGFBR2,APPL1,DVL2,CDH2,WNT7A,TGFB1,DKK3,PPP2R3A,GNAO1,FZD6,MAP3K7,TGFB2,AKT3,PIN1,PPP2R1B,FZD7
ErbB2-ErbB3 Signaling	1.760	0.176	-2.111	RRAS2,PIK3C2A,GRB2,IRS1,FGFR1,MRAS,IRS2,ERBB2,PIK3R2,CDKN1B,CCND1,ATM
EGF Signaling	1.760	0.176	-0.577	SRC,MAPK14,PIK3C2A,GRB2,IRS1,FGFR1,AKT3,IRS2,PIK3R2,MAPK12,ATM,EGFR
GDNF Family Ligand-Receptor Interactions	1.760	0.171	-1.387	PIK3C2A,GRB2,FGFR1,RAC1,MAPK12,RRAS2,GFRA1,IRS1,MRAS,IRS2,DOK1,PIK3R2,ATM
Fc Epsilon RI Signaling	1.700	0.151	-0.943	RAC2,PIK3C2A,GRB2,FGFR1,RAC1,INPPL1,MAPK12,SYNJ2,MAPK14,RRAS2,PRKCD,IRS1,LYN,MRAS,AKT3,IRS2,PIK3R2,ATM
Neuregulin Signaling	1.680	0.163	-1.265	ITGB1,SRC,GRB2,ERBIN,ITGA5,ITGA3,RRAS2,PRKCD,MRAS,AKT3,CDKN1B,PIK3R2,ERBB2,EGFR
ErbB4 Signaling	1.670	0.171	-1.265	RRAS2,YAP1,PIK3C2A,GRB2,IRS1,PRKCD,FGFR1,MRAS,PSEN2,IRS2,PIK3R2,ATM
Natural Killer Cell Signaling	1.670	0.150	NaN	RAC2,PAK4,PIK3C2A,GRB2,FGFR1,RAC1,INPPL1,SYNJ2,PAK1,RRAS2,PRKCD,IRS1,PAK2,MRAS,AKT3,IRS2,PIK3R2,ATM
PRPP Biosynthesis I	1.640	0.667	NaN	PRPS2,PRPS1
Tetrahydrobiopterin Biosynthesis I	1.640	0.667	NaN	PTS,SPR
D-glucuronate Degradation I	1.640	0.667	NaN	AKR1A1,DCXR
Glycerol-3-phosphate Shuttle	1.640	0.667	NaN	GPD2,GPD1L
Tetrahydrobiopterin Biosynthesis II	1.640	0.667	NaN	PTS,SPR
N-acetylglucosamine Degradation I	1.640	0.667	NaN	AMDHD2,GNPDA2
CREB Signaling in Neurons	1.640	0.133	-1.342	PRKACB,GNA11,CACNA1F,MRAS,PRKAR1B,AKT3,IRS2,PIK3R2,GNG5,GNG12,ATM,PIK3C2A,GRB2,GNA12,FGFR1,GNG2,ADCY6,GNAI1,PRKAR2A,GNG10,GNAI2,GNAI3,PLCB4,RRAS2,IRS1,PRKCD,GNAO1,PRKACA
FcγRIIB Signaling in B Lymphocytes	1.630	0.165	-1.155	PIK3C2A,GRB2,FGFR1,MAPK12,CACNA1F,RRAS2,IRS1,LYN,MRAS,IRS2,DOK1,PIK3R2,ATM
G-Protein Coupled Receptor Signaling	1.630	0.127	NaN	PRKACB,RELA,NFKBIE,GNA11,GDE1,RGS10,MRAS,PRKAR1B,AKT3,SMPDL3B,IRS2,PIK3R2,CHUK,PDE11A,NFKBIB,ATM,SMPDL3A,SRC,PIK3C2A,GRB2,FGFR1,ADCY6,GNAI1,PRKAR2A,IKBKE,RAP1A,PDE1C,GNAI2,GNAI3,PLCB4,RRAS2,LPAR1,IRS1,GNAO1,PRKACA
Urate Biosynthesis/Inosine 5'-phosphate Degradation	1.620	0.308	1.000	XDH,IMPDH1,PNP,NT5C
Estrogen-mediated S-phase Entry	1.600	0.231	-0.816	RB1,E2F4,CCNE2,CDKN1B,CCND1,CDK2
ATM Signaling	1.580	0.155	0.277	ATF1,PPP2R5B,ABL1,MDC1,CBX5,MAPK12,KAT5,MAPK14,CBX1,PPP2R3A,H2AFX,BID,PPP2R1B,CDK2,ATM
Factors Promoting Cardiogenesis in Vertebrates	1.560	0.157	NaN	CCNE2,TGFBR1,DVL1,SMAD5,TGFBR2,MAPK14,TGFB1,PRKCD,FZD6,MAP3K7,TGFB2,CDK2,FZD7,BMP1
Tryptophan Degradation X (Mammalian, via Tryptamine)	1.540	0.250	1.342	ALDH2,AKR1A1,ALDH3A2,MAOA,IL4I1
Hypoxia Signaling in the Cardiovascular System	1.540	0.164	0.000	UBE2J1,UBE2M,NFKBIE,UBE2R2,NQO1,UBE2V1,HIF1AN,UBE2W,NFKBIB,VHL,ARNT,ATM
Dopamine-DARPP32 Feedback in cAMP Signaling	1.530	0.138	-0.894	PRKACB,PPP1R14C,ATF1,PPP2R5B,PRKAR2A,KCNJ16,GNAI1,ADCY6,ATP2A2,GNAI2,GNAI3,PLCB4,PPP1R10,PPP1R12A,PPP3CB,PPP2R3A,PRKCD,CAMKK1,PRKACA,PRKAR1B,PPP2R1B,GUCY1B1
Superpathway of Cholesterol Biosynthesis	1.520	0.222	0.816	SQLE,NSDHL,MSMO1,LSS,HMGCR,LBR

Ingenuity Canonical Pathways	-log(p-value)	Ratio	z-score	Proteins
mTOR Signaling	1.510	0.132	-1.000	TSC1,RHEB,PIK3C2A,RHOC,GRB2,FGFR1,PPP2R5B,RAC1,RPS6KA5,PDGFC,PLD1,EIF4EBP1,ATG13,HMOX1,RRAS2,PPP2R3A,PRKCD,IRS1,INS,MRAS,AKT3,IRS2,PIK3R2,PPP2R1B,AKT1S1,ATM
Phenylalanine Degradation IV (Mammalian, via Side Chain)	1.500	0.286	2.000	ALDH2,ALDH3A2,MAOA,IL4I1
Colanic Acid Building Blocks Biosynthesis	1.500	0.286	1.000	GMPPB,GPI,GMDS,GALT
ERK5 Signaling	1.490	0.167	-0.905	IL6ST,SRC,RRAS2,YWHAH,GNA12,MRAS,RPS6KA5,MAPK7,WNK1,MAP3K2,EGFR
T Cell Receptor Signaling	1.490	0.148	NaN	RELA,PIK3C2A,GRB2,FGFR1,RAC1,IKBKE,NFAT5,RRAS2,PPP3CB,PAG1,IRS1,MRAS,IRS2,CHUK,PIK3R2,ATM
Noradrenaline and Adrenaline Degradation	1.470	0.200	1.890	HSD17B10,ALDH2,AKR1A1,ALDH3A2,PECR,MAOA,IL4I1
JAK/Stat Signaling	1.470	0.157	-1.387	RELA,RRAS2,PIK3C2A,GRB2,IRS1,FGFR1,MRAS,AKT3,STAT2,IRS2,CEBPB,PIK3R2,ATM
Pyrimidine Ribonucleotides Interconversion	1.450	0.186	0.000	SMARCA1,AK5,CMPK2,CTPS1,CANT1,NME7,RECQL,ENTPD7
Retinoic acid Mediated Apoptosis Signaling	1.440	0.169	1.265	TNKS,CASP3,PARP10,TNFRSF10B,BID,TNFSF10,PARP12,CFLAR,TNFRSF10A,PARP14
Amyotrophic Lateral Sclerosis Signaling	1.430	0.145	-1.807	CAPN5,CASP3,PIK3C2A,GRB2,FGFR1,GPX1,RAC1,PDGFC,PAK1,IRS1,BID,AKT3,IRS2,PIK3R2,CASP7,ATM
GPCR-Mediated Nutrient Sensing in Enteroendocrine Cells	1.400	0.144	-1.000	PRKACB,GNG2,GNA11,PRKAR2A,GNAI1,ADCY6,GNG10,CACNA1F,GNAI2,GNAI3,PLCB4,PRKCD,PRKACA,PRKAR1B,GNG5,GNG12
Prostanoid Biosynthesis	1.390	0.333	NaN	PTGES,PTGS1,TBXAS1
iNOS Signaling	1.390	0.182	-1.134	RELA,MAPK14,NFKBIE,IKBKE,CHUK,HMGA1,NFKBIB,MAPK12
Arsenate Detoxification I (Glutaredoxin)	1.370	0.500	NaN	AS3MT,PNP
Heme Degradation	1.370	0.500	NaN	HMOX1,HMOX2
Phenylethylamine Degradation I	1.370	0.500	NaN	ALDH2,ALDH3A2
Proline Biosynthesis I	1.370	0.500	NaN	PYCR2,PYCR1
Melatonin Degradation II	1.370	0.500	NaN	MAOA,IL4I1
N-acetylglucosamine Degradation II	1.370	0.500	NaN	AMDHD2,GNPDA2
IL-10 Signaling	1.360	0.159	NaN	RELA,HMOX1,MAPK14,NFKBIE,MAP3K7,IL1B,IKBKE,CHUK,NFKBIB,MAPK12,MAP4K4
Dendritic Cell Maturation	1.360	0.130	-1.633	RELA,PIK3C2A,GRB2,NFKBIE,FGFR1,HLA-B,CD58,IKBKE,MAPK12,COL1A1,PLCB4,MAPK14,NGFR,IRS1,AKT3,IL1B,IRS2,STAT2,PIK3R2,CHUK,NFKBIB,TNFRSF11B,ATM,COL3A1
IL-9 Signaling	1.340	0.178	-1.414	RELA,PIK3C2A,GRB2,IRS1,FGFR1,IRS2,PIK3R2,ATM
Pyrimidine Ribonucleotides De Novo Biosynthesis	1.340	0.178	0.000	SMARCA1,AK5,CMPK2,CTPS1,CANT1,NME7,RECQL,ENTPD7
Role of Pattern Recognition Receptors in Recognition of Bacteria and Viruses	1.330	0.137	-0.277	PTX3,RELA,C3,PIK3C2A,GRB2,FGFR1,OAS3,MAPK12,RNASEL,TGFB1,IRS1,PRKCD,TGFB2,IL1B,IRS2,PIK3R2,RIPK2,ATM
Melatonin Signaling	1.320	0.157	-0.632	PRKACB,GNAI2,GNAI3,PLCB4,ARAF,PRKCD,GNAO1,PRKAR2A,PRKACA,PRKAR1B,GNAI1
Extrinsic Prothrombin Activation Pathway	1.310	0.250	NaN	F5,F13A1,FGB,TFPI
Parkinson's Signaling	1.310	0.250	NaN	MAPK14,CASP3,MAPK12,SNCA
Hereditary Breast Cancer Signaling	1.310	0.135	NaN	PIK3C2A,GRB2,ACTB,FGFR1,CDK6,RFC1,DDB2,CCND1,RB1,RRAS2,IRS1,H2AFX,XPC,MRAS,MSH6,AKT3,IRS2,PIK3R2,ATM
RAR Activation	1.310	0.128	NaN	PRKACB,SRC,RELA,SMAD3,ACTB,NR2F2,PRKAR2A,RAC1,ADCY6,SMAD5,MAPK12,TNIP1,MAPK14,TGFB1,ERCC3,PRKCD,PRKACA,IGFBP3,PRKAR1B,TGFB2,AKT3,NRIP1,PIK3R2,NCOR2
Osteoarthritis Pathway	1.310	0.126	-1.460	RELA,TGFBR1,FN1,SMAD3,CTNNA1,CASP4,SMAD5,SDC4,PDGFC,HMGB1,TGFBR2,ITGA3,TGFB1,CHUK,ITGB1,SPP1,CASP3,FGFR1,RAC1,ITGA5,CEBPB,SIRT1,FZD6,IL1B,CASP7,FZD7
Inhibition of Matrix Metalloproteases	1.300	0.184	0.000	HSPG2,MMP7,SDC1,SDC2,TFPI2,A2M,TIMP2
Antigen Presentation Pathway	1.300	0.184	NaN	PSMB9,HLA-B,PSMB8,CD74,TAPBP,TAP1,TAP2
autophagy	1.300	0.167	NaN	ATG13,STX17,CTSA,CTSL,CTSS,RB1CC1,LAMP1,MAP1LC3A,MAP1LC3B2
Oleate Biosynthesis II (Animals)	1.270	0.300	NaN	SCD5,FADS2,CYB5A
Systemic Lupus Erythematosus Signaling	1.270	0.124	NaN	CD2BP2,HNRNPA2B1,HLA-B,LSM14A,NFAT5,MRAS,AKT3,IRS2,LSM3,PRPF40B,SNRPD2,PIK3R2,ATM,LSM14B,LSM6,PIK3C2A,GRB2,SNRNP27,FGFR1,PPIH,RRAS2,LSM8,LSM12,SNRPC,IRS1,LYN,IL1B

Ingenuity Canonical Pathways	-log(p-value)	Ratio	z-score	Proteins
Type I Diabetes Mellitus Signaling	1.250	0.140	-0.277	RELA,CASP3,NFKBIE,HLA-B,IKBKE,MAPK12,MAPK14,NGFR,INS,MAP3K7,BID,IL1B,CHUK,NFKBIB,TNFRSF11B
IL-2 Signaling	1.230	0.156	-1.265	RRAS2,PIK3C2A,GRB2,IRS1,FGFR1,MRAS,AKT3,IRS2,PIK3R2,ATM
Mitochondrial L-carnitine Shuttle Pathway	1.220	0.235	1.000	ACSL3,CPT1A,ACSL4,SLC27A4
Ethanol Degradation II	1.200	0.188	0.816	HSD17B10,ALDH2,AKR1A1,ALDH3A2,ACSS2,PECR
Triacylglycerol Degradation	1.200	0.175	-0.378	DDHD2,PPME1,ABHD2,DAGLB,CES2,NDST1,ABHD16A
Thrombopoietin Signaling	1.190	0.154	-1.265	RRAS2,PIK3C2A,GRB2,IRS1,PRKCD,FGFR1,MRAS,IRS2,PIK3R2,ATM
eNOS Signaling	1.180	0.127	-1.528	PRKACB,CASP3,PIK3C2A,GRB2,FGFR1,PRKAR2A,ADCY6,PDGFC,HSPA2,NOSIP,LPAR1,STUB1,PRKCD,IRS1,PRKACA,PRKAR1B,AKT3,IRS2,PIK3R2,GUCY1B1,ATM
Serine Biosynthesis	1.170	0.400	NaN	PSAT1,PHGDH
Tetrapyrrole Biosynthesis II	1.170	0.400	NaN	UROS,HMBS
IL-17A Signaling in Gastric Cells	1.160	0.200	0.000	RELA,MAPK14,CCL20,MAPK12,EGFR
Cholecystokinin/Gastrin-mediated Signaling	1.160	0.139	-1.604	ROCK1,SRC,PLCB4,RRAS2,MAPK14,GRB2,RHOC,PRKCD,GNA12,MRAS,IL1B,MAPK7,MAPK12,EGFR
Neuroinflammation Signaling Pathway	1.150	0.116	-0.343	RELA,TRAF3,TGFBR1,PYCARD,HLA-B,PSEN2,TGFBR2,HMGB1,HMOX1,SOD2,NFAT5,PPP3CB,TGFB1,TGFB2,AKT3,IRS2,PIK3R2,CHUK,ATM,VCAM1,CASP3,PIK3C2A,GRB2,GLS,FGFR1,IKBKE,TBK1,MAPK12,MAPK14,IRS1,IL1B,BACE2,CFLAR,MAPK7,SNCA
Purine Nucleotides Degradation II (Aerobic)	1.140	0.222	1.000	XDH,IMPDH1,PNP,NT5C
Antioxidant Action of Vitamin C	1.080	0.135	1.155	RELA,HMOX1,SLC23A2,PLCB4,MAPK14,NFKBIE,IKBKE,TXN,CHUK,NFKBIB,MAPK12,PAFAH1B1,PLD1,PAFAH1B3
Putrescine Degradation III	1.070	0.211	2.000	ALDH2,ALDH3A2,MAOA,IL4I1
DNA damage-induced 14-3-3σ Signaling	1.070	0.211	NaN	CCNE2,AKT3,CDK2,ATM
Phagosome Formation	1.070	0.130	NaN	ITGB1,FN1,PIK3C2A,GRB2,RHOC,FGFR1,ITGA5,INPPL1,MARCKS,ITGA3,PLCB4,IRS1,PRKCD,IRS2,PIK3R2,ATM
Guanosine Nucleotides Degradation III	1.060	0.250	NaN	XDH,PNP,NT5C
Neuropathic Pain Signaling In Dorsal Horn Neurons	1.060	0.132	-1.291	PRKACB,SRC,PIK3C2A,CAMK1D,GRB2,FGFR1,PRKAR2A,PLCB4,IRS1,PRKCD,PRKACA,PRKAR1B,IRS2,PIK3R2,ATM
Xanthine and Xanthosine Salvage	1.050	1.000	NaN	PNP
L-cysteine Degradation II	1.050	1.000	NaN	CTH
Asparagine Biosynthesis I	1.050	1.000	NaN	ASNS
Phosphatidylethanolamine Biosynthesis III	1.050	1.000	NaN	PTDSS2
Lanosterol Biosynthesis	1.050	1.000	NaN	LSS
Bladder Cancer Signaling	1.050	0.138	-1.000	CDKN2A,RB1,MMP7,RRAS2,THBS1,MRAS,ABL1,RPS6KA5,ERBB2,PDGFC,CCND1,EGFR
NAD Biosynthesis from 2-amino-3-carboxymuconate Semialdehyde	1.020	0.333	NaN	NMNAT1,ABL1
Glycine Cleavage Complex	1.020	0.333	NaN	GCSH,GLDC
GDP-mannose Biosynthesis	1.020	0.333	NaN	GMPPB,GPI
VEGF Family Ligand-Receptor Interactions	1.020	0.136	-0.905	RRAS2,NRP2,PIK3C2A,GRB2,IRS1,PRKCD,FGFR1,MRAS,AKT3,IRS2,PIK3R2,ATM
Inflammasome pathway	1.000	0.200	1.000	PYCARD,IL1B,NEK7,PANX1
TR/RXR Activation	0.997	0.133	NaN	PIK3C2A,GRB2,FGFR1,SLC16A3,LDLR,SREBF2,IRS1,SLC16A2,AKT3,IRS2,NCOR2,PIK3R2,ATM
CTLA4 Signaling in Cytotoxic T Lymphocytes	0.997	0.133	NaN	AP1G2,PIK3C2A,GRB2,PPP2R3A,IRS1,FGFR1,HLA-B,PPP2R5B,AKT3,IRS2,PIK3R2,PPP2R1B,ATM
Fatty Acid Activation	0.978	0.231	NaN	ACSL3,ACSL4,SLC27A4
NAD biosynthesis II (from tryptophan)	0.978	0.231	NaN	KMO,NMNAT1,ABL1
Choline Biosynthesis III	0.978	0.231	NaN	HMOX1,PLD1,PCYT1A
Mitotic Roles of Polo-Like Kinase	0.958	0.143	-2.236	FZR1,SLK,PPP2R3A,TGFB1,PPP2R5B,ANAPC7,PPP2R1B,CDC27,STAG2
GPCR-Mediated Integration of Enteroendocrine Signaling Exemplified by an L Cell	0.957	0.139	-0.632	PRKACB,GNAI2,GNAI3,PLCB4,GNA11,PRKAR2A,PRKACA,PRKAR1B,ADCY6,GNAI1
Sonic Hedgehog Signaling	0.942	0.172	1.000	PRKACB,ARRB2,PRKAR2A,PRKACA,PRKAR1B
Synaptic Long Term Potentiation	0.941	0.126	-1.807	PRKACB,RAP1B,PPP1R14C,GNA11,PRKAR2A,RAP1A,PLCB4,RRAS2,PPP1R12A,PPP3CB,PPP1R10,PRKCD,PRKACA,PRKAR1B,MRAS

Ingenuity Canonical Pathways	-log(p-value)	Ratio	z-score	Proteins
FGF Signaling	0.938	0.132	-0.577	MAPK14,PIK3C2A,GRB2,IRS1,FGFR1,RAC1,AKT3,IRS2,RPS6KA5,PIK3R2,MAPK12,ATM
Prolactin Signaling	0.905	0.133	-1.265	RRAS2,PIK3C2A,GRB2,IRS1,PRKCD,FGFR1,MRAS,IRS2,CEBPB,PIK3R2,ATM
Superpathway of Serine and Glycine Biosynthesis I	0.901	0.286	NaN	PSAT1,PHGDH
Ceramide Biosynthesis	0.901	0.286	NaN	SPTLC2,KDSR
Fatty Acid β-oxidation I	0.894	0.167	1.342	HSD17B10,ACSL3,SCP2,ACSL4,SLC27A4
CCR5 Signaling in Macrophages	0.887	0.129	0.378	GNAI2,CACNA1F,GNAI3,MAPK14,PRKCD,GNG2,MRAS,GNAI1,MAPK12,GNG5,GNG12,GNG10
Cysteine Biosynthesis III (mammalia)	0.885	0.182	0.000	CBS/CBSL,PRMT3,AHCYL2,CTH
Role of CHK Proteins in Cell Cycle Checkpoint Control	0.863	0.140	-0.816	E2F4,PPP2R3A,PPP2R5B,MDC1,RFC1,PPP2R1B,CDK2,ATM
Growth Hormone Signaling	0.853	0.129	-0.302	PIK3C2A,GRB2,IRS1,PRKCD,FGFR1,IGFBP3,IRS2,RPS6KA5,PIK3R2,A2M,ATM
Cytotoxic T Lymphocyte-mediated Apoptosis of Target Cells	0.849	0.161	1.000	CASP3,HLA-B,BID,DFFB,CASP7
Salvage Pathways of Pyrimidine Ribonucleotides	0.839	0.126	-1.155	PAK1,AK5,CMPK2,ARAF,PRKCD,PAK2,CDK6,MAPK7,CDK2,APOBEC3G,NME7,LIMK1
Telomere Extension by Telomerase	0.833	0.200	NaN	TNKS,HNRNPA2B1,TERF1
Superpathway of Citrulline Metabolism	0.833	0.200	NaN	GLS,OAT,ASL
Adenosine Nucleotides Degradation II	0.833	0.200	NaN	XDH,PNP,NT5C
Atherosclerosis Signaling	0.833	0.121	NaN	RELA,VCAM1,PDGFC,PDGFB,APOL1,COL1A1,ALB,TGFB1,IL1B,SERPINA1,PAFAH1B1,CLU,PA FAH1B3,COL3A1,APOC3
VDR/RXR Activation	0.818	0.130	NaN	SERPINB1,SPP1,PRKCD,IGFBP3,TGFB2,IGFBP5,SEMA3B,CEBPB,CDKN1B,NCOR2
Neurotrophin/TRK Signaling	0.818	0.130	-1.265	RRAS2,PIK3C2A,GRB2,IRS1,NGFR,FGFR1,MRAS,IRS2,PIK3R2,ATM
Corticotropin Releasing Hormone Signaling	0.808	0.119	-1.000	RAP1B,PRKACB,PRKAR2A,GNAI1,ADCY6,MAPK12,RAP1A,CACNA1F,GNAI2,GNAI3,MAPK14, PRKCD,GNAO1,PRKACA,PRKAR1B,GUCY1B1
Superoxide Radicals Degradation	0.802	0.250	NaN	SOD2,NQO1
Mitochondrial Dysfunction	0.799	0.115	NaN	HSD17B10,ATP5PF,CPT1A,CASP3,UQCRH,XDH,COX5B,PSEN2,DHODH,MAPK12,CYB5R3,VPS9 D1,SOD2,GPD2,BACE2,CYB5A,SNCA,MAOA,COX15
Role of JAK1 and JAK3 in γc Cytokine Signaling	0.782	0.130	NaN	RRAS2,PIK3C2A,GRB2,IRS1,FGFR1,MRAS,IRS2,PIK3R2,ATM
Epoxysqualene Biosynthesis	0.765	0.500	NaN	SQLE
Guanine and Guanosine Salvage I	0.765	0.500	NaN	PNP
Glycine Degradation (Creatine Biosynthesis)	0.765	0.500	NaN	GAMT
Adenine and Adenosine Salvage I	0.765	0.500	NaN	PNP
GDP-L-fucose Biosynthesis I (from GDP-D-mannose)	0.765	0.500	NaN	GMDS
Glutamine Degradation I	0.765	0.500	NaN	GLS
Glutamate Biosynthesis II	0.765	0.500	NaN	GLUD1
Glutamate Degradation X	0.765	0.500	NaN	GLUD1
Aldosterone Signaling in Epithelial Cells	0.765	0.114	0.000	PIK3C2A,DNAJB4,GRB2,FGFR1,PIKFYVE,PIP4K2B,DNAJB2,HSPA2,HSPA12A,PLCB4,PRKCD,IR S1,HSPA13,PIP5KL1,IRS2,DNAJB6,PIK3R2,ATM,HSPA4L
tRNA Splicing	0.763	0.143	0.000	SMPDL3A,GDE1,TRPT1,SMPDL3B,PDE11A,PDE1C
Role of BRCA1 in DNA Damage Response	0.743	0.125	-1.342	RB1,E2F4,ABRAXAS1,ATF1,ACTB,MSH6,MDC1,RFC1,BABAM2,ATM
Stearate Biosynthesis I (Animals)	0.730	0.140	0.816	ACSL3,PORCN,ACSL4,TBXAS1,SLC27A4,ACOT8
DNA Methylation and Transcriptional Repression Signaling	0.728	0.147	NaN	SAP18,DNMT3A,MBD3,SAP130,DNMT1
Citrulline Biosynthesis	0.719	0.222	NaN	GLS,OAT
Heme Biosynthesis II	0.719	0.222	NaN	UROS,HMBS
GDP-glucose Biosynthesis	0.719	0.222	NaN	HK2,PGM5
Oxidative Ethanol Degradation III	0.715	0.176	NaN	ALDH2,ALDH3A2,ACSS2
MSP-RON Signaling Pathway	0.705	0.125	NaN	PIK3C2A,GRB2,IRS1,ACTB,FGFR1,IRS2,PIK3R2,ACTG1,ATM
Superpathway of Methionine Degradation	0.692	0.143	-0.447	CBS/CBSL,PRMT3,PCCB,AHCYL2,CTH
Protein Ubiquitination Pathway	0.686	0.106	NaN	FZR1,PSMB10,DNAJB4,USP11,HLA- B,USP54,UBE2W,PSMB8,DNAJB2,TAP1,STUB1,BAG1,UBE2V1,USP10,USP40,HSPA4L,PSMB9,U BE2M,UBE2R2,HSPA2,HSPA12A,UBE2J1,USP32,CUL2,HSPA13,DNAJB6,TAP2,VHL
Adipogenesis pathway	0.683	0.114	NaN	SAP18,TXNIP,FGFR1,SMAD3,NR2F2,SAP130,SMAD5,CEBPB,RB1,TGFB1,ERCC3,AGPAT2,SIRT 1,FZD6,FZD7

Ingenuity Canonical Pathways	-log(p-value)	Ratio	z-score	Proteins
Pyridoxal 5'-phosphate Salvage Pathway	0.667	0.125	-2.121	PAK1,ARAF,PRKCD,PAK2,CDK6,MAPK7,CDK2,LIMK1
D-myo-inositol (1,4,5)-Trisphosphate Biosynthesis	0.655	0.148	1.000	PLCB4,PIKFYVE,PIP5KL1,PIP4K2B
Glucose and Glucose-1-phosphate Degradation	0.647	0.200	NaN	HK2,PGM5
Serotonin Degradation	0.642	0.123	2.121	HSD17B10,ALDH2,AKR1A1,ALDH3A2,PECR,B3GAT3,MAOA,IL4I1
1D-myo-inositol Hexakisphosphate Biosynthesis II (Mammalian)	0.617	0.158	NaN	SYNJ2,IPMK,INPPL1
D-myo-inositol (1,3,4)-trisphosphate Biosynthesis	0.617	0.158	NaN	SYNJ2,IPMK,INPPL1
Granulocyte Adhesion and Diapedesis	0.611	0.108	NaN	ITGB1,VCAM1,MMP7,SDC1,GNAI1,ITGA5,CCL20,SDC4,ITGB3,GNAI2,GNAI3,ITGA3,SDC2,CLDN1,NGFR,IL1B,ITGA1,TNFRSF11B
1,25-dihydroxyvitamin D3 Biosynthesis	0.608	0.333	NaN	POR
4-hydroxyproline Degradation I	0.608	0.333	NaN	HOGA1
1D-myo-inositol Hexakisphosphate Biosynthesis V (from Ins(1,3,4)P3)	0.608	0.333	NaN	IPMK
Oxidized GTP and dGTP Detoxification	0.608	0.333	NaN	DDX6
Tyrosine Biosynthesis IV	0.608	0.333	NaN	PCBD2
4-aminobutyrate Degradation I	0.608	0.333	NaN	SUCLG2
Iron homeostasis signaling pathway	0.599	0.110	NaN	ATP6V0C,TCIRG1,GDF15,ATP6AP1,PDGFB,HMOX2,ARNT,HMOX1,TF,FXN,TFRC,STEAP3,EGFR,BMP1
Synaptic Long Term Depression	0.597	0.107	-0.471	GNA12,PPP2R5B,GNA11,GNAI1,GNAI2,CACNA1F,GNAI3,PLCB4,RRAS2,PPP2R3A,PRKCD,GNAO1,LYN,MRAS,PAFAH1B1,PPP2R1B,GUCY1B1,PAFAH1B3
Dolichyl-diphosphooligosaccharide Biosynthesis	0.585	0.182	NaN	DPAGT1,ALG1
Gαs Signaling	0.561	0.110	-1.265	PRKACB,SRC,GNG2,PRKAR2A,PRKACA,PRKAR1B,MRAS,ADCY6,GNG5,RAP1A,GNG12,GNG10
Cancer Drug Resistance By Drug Efflux	0.558	0.122	NaN	ABCB1,RRAS2,ARAF,MRAS,AKT3,PIK3R2
Dopamine Degradation	0.549	0.133	2.000	ALDH2,ALDH3A2,MAOA,IL4I1
Sperm Motility	0.548	0.108	-1.387	PRKACB,MAP3K11,PRKAR2A,PDE1C,PLCB4,PRKCD,MRAS,PRKAR1B,PRKACA,PAFAH1B1,GUCY1B1,PTK7,PAFAH1B3
NAD Salvage Pathway II	0.535	0.143	NaN	NUDT12,NMNAT1,NT5C
Ethanol Degradation IV	0.535	0.143	NaN	ALDH2,ALDH3A2,ACSS2
cAMP-mediated signaling	0.535	0.102	-0.447	SMPDL3A,PRKACB,AKAP5,SRC,CAMK1D,PRKAR2A,GNAI1,ADCY6,RAP1A,PDE1C,GNAI2,AKAP2,GNAI3,LPAR1,PPP3CB,GDE1,RGS10,GNAO1,PRKACA,PRKAR1B,SMPDL3B,PDE11A,AKAP1
Role of MAPK Signaling in the Pathogenesis of Influenza	0.533	0.114	NaN	RRAS2,MAPK14,CASP3,MRAS,AKT3,MAPK12,PAFAH1B1,PAFAH1B3
Cleavage and Polyadenylation of Pre-mRNA	0.530	0.167	NaN	CPSF4,WDR33
Intrinsic Prothrombin Activation Pathway	0.512	0.122	0.447	COL1A1,F5,F13A1,FGB,COL3A1
Uracil Degradation II (Reductive)	0.503	0.250	NaN	DPYSL2
2-ketoglutarate Dehydrogenase Complex	0.503	0.250	NaN	DLST
Spermine and Spermidine Degradation I	0.503	0.250	NaN	PAOX
Methylmalonyl Pathway	0.503	0.250	NaN	PCCB
Thymine Degradation	0.503	0.250	NaN	DPYSL2
Geranylgeranyldiphosphate Biosynthesis	0.503	0.250	NaN	FNTB
Arginine Degradation I (Arginase Pathway)	0.503	0.250	NaN	OAT
NAD Biosynthesis III	0.503	0.250	NaN	NMNAT1
Phenylalanine Degradation I (Aerobic)	0.503	0.250	NaN	PCBD2
Molybdenum Cofactor Biosynthesis	0.503	0.250	NaN	MOCS2
Acetate Conversion to Acetyl-CoA	0.503	0.250	NaN	ACSS2
Wnt/Ca+ pathway	0.491	0.113	-1.134	RELA,DVL2,PLCB4,NFAT5,DVL1,FZD6,FZD7
Serotonin Receptor Signaling	0.487	0.119	NaN	PTS,ADCY6,SPR,MAOA,IL4I1
Agranulocyte Adhesion and Diapedesis	0.483	0.102	NaN	ITGB1,VCAM1,MMP7,FN1,MYL6,ACTB,GNAI1,ITGA5,CCL20,SDC4,MYL9,GNAI2,GNAI3,ITGA3,CLDN1,IL1B,ITGA1,ACTG1
LPS/IL-1 Mediated Inhibition of RXR Function	0.480	0.100	0.000	ABCB1,ACSL3,CPT1A,CHST7,ACOX1,SLC35A2,CES2,SLC27A4,GSTT1,IL4I1,ALDH2,ALDH3A2,NGFR,MAP3K7,IL1B,ACSL4,PLTP,ABCC3,NDST1,MAOA,TNFRSF11B
MIF Regulation of Innate Immunity	0.463	0.116	-1.342	RELA,NFKBIE,CD74,NFKBIB,MAPK12
Cellular Effects of Sildenafil (Viagra)	0.462	0.103	NaN	PRKACB,MYL6,ACTB,PRKAR2A,ADCY6,PDE1C,MYL9,PLCB4,PPP1R12A,PRKAR1B,PRKACA,ACTG1,GUCY1B1

Ingenuity Canonical Pathways	-log(p-value)	Ratio	z-score	Proteins
Toll-like Receptor Signaling	0.441	0.107	0.000	RELA,MAPK14,TAB2,MAP3K7,IL1B,CHUK,MAPK12,MAP4K4
DNA Double-Strand Break Repair by Homologous Recombination	0.440	0.143	NaN	ABL1,ATM
Isoleucine Degradation I	0.440	0.143	NaN	HSD17B10,ACADSB
γ-glutamyl Cycle	0.440	0.143	NaN	GGT1,ANPEP
Oncostatin M Signaling	0.436	0.118	-1.000	IL6ST,RRAS2,GRB2,MRAS
Estrogen Receptor Signaling	0.436	0.102	NaN	SRC,GRB2,HNRNPD,RBFOX2,GTF2A1,DDX5,RRAS2,MED15,ERCC3,MED21,MRAS,NRIP1,NCOR2
IL-22 Signaling	0.434	0.125	NaN	MAPK14,AKT3,MAPK12
Superpathway of D-myo-inositol (1,4,5)-trisphosphate Metabolism	0.434	0.125	NaN	SYNJ2,IPMK,INPPL1
TCA Cycle II (Eukaryotic)	0.434	0.125	NaN	CS,SUCLG1,DLST
Tryptophan Degradation III (Eukaryotic)	0.434	0.125	NaN	HSD17B10,KMO,CDYL
Glycolysis I	0.434	0.125	NaN	GPI,ENO2,PFKM
Trehalose Degradation II (Trehalase)	0.425	0.200	NaN	HK2
Protein Citrullination	0.425	0.200	NaN	PADI2
Creatine-phosphate Biosynthesis	0.425	0.200	NaN	MAP4K4
Glycerol Degradation I	0.425	0.200	NaN	GPD2
NAD Salvage Pathway III	0.425	0.200	NaN	NMNAT1
Myo-inositol Biosynthesis	0.425	0.200	NaN	ISYNA1
2-oxobutanoate Degradation I	0.425	0.200	NaN	PCCB
Citrulline-Nitric Oxide Cycle	0.425	0.200	NaN	ASL
Galactose Degradation I (Leloir Pathway)	0.425	0.200	NaN	GALT
Lactose Degradation III	0.425	0.200	NaN	PSAP
dTMP De Novo Biosynthesis	0.425	0.200	NaN	TYMS
Tyrosine Degradation I	0.425	0.200	NaN	FAH
Glutamate Degradation III (via 4-aminobutyrate)	0.425	0.200	NaN	SUCLG2
Folate Polyglutamylation	0.425	0.200	NaN	FPGS
Role of Oct4 in Mammalian Embryonic Stem Cell Pluripotency	0.419	0.111	NaN	FAM208A,RB1,SPP1,NR2F2,IGF2BP1
Gustation Pathway	0.416	0.099	NaN	SMPDL3A,PRKACB,P2RX4,GNG2,PRKAR2A,ADCY6,PANX1,PDE1C,CACNA1F,GDE1,PRKACA,PRKAR1B,SMPDL3B,GNG5,PDE11A
MIF-mediated Glucocorticoid Regulation	0.411	0.114	-2.000	RELA,NFKBIE,CD74,NFKBIB
OX40 Signaling Pathway	0.407	0.107	1.342	RELA,TRAF3,NFKBIE,HLA-B,NFKBIB,MAPK12
Glutamate Receptor Signaling	0.407	0.107	NaN	HOMER2,GLS,GNG2,SLC38A1,HOMER3,GNG5
Role of JAK family kinases in IL-6-type Cytokine Signaling	0.405	0.120	NaN	IL6ST,MAPK14,MAPK12
Gluconeogenesis I	0.405	0.120	NaN	GPI,ENO2,ME2
p38 MAPK Signaling	0.403	0.100	0.577	TGFBR2,TGFBR1,MAPK14,ATF1,TGFB1,TAB2,TGFB2,MAP3K7,IL1B,RPS6KA5,EEF2K,MAPK12
Dermatan Sulfate Degradation (Metazoa)	0.402	0.133	NaN	TMEM2,IDS
Histamine Degradation	0.402	0.133	NaN	ALDH2,ALDH3A2
Vitamin-C Transport	0.402	0.133	NaN	SLC23A2,TXN
Sirtuin Signaling Pathway	0.393	0.095	1.043	RELA,SOD2,CRTC2,H1FX,XPC,ACSS2,MAP1LC3A,ATP5PF,PFKFB3,CPT1A,PPIF,NDRG1,GLS,NQO1,TUBA4A,GLUD1,MAPK12,AGTRAP,MAP1LC3B2,PFKM,ATG13,HIST1H1B,HIST1H1E,SIRT1,TOMM34,HIST1H1D,MAPK7
Mismatch Repair in Eukaryotes	0.368	0.125	NaN	MSH6,RFC1
Glutaryl-CoA Degradation	0.368	0.125	NaN	HSD17B10,CDYL
Superpathway of Geranylgeranyldiphosphate Biosynthesis I (via Mevalonate)	0.368	0.125	NaN	FNTB,HMGCR
T Helper Cell Differentiation	0.368	0.101	NaN	IL6ST,TGFBR2,TGFBR1,TGFB1,NGFR,HLA-B,TNFRSF11B
Urea Cycle	0.364	0.167	NaN	ASL
Chondroitin and Dermatan Biosynthesis	0.364	0.167	NaN	CHPF
UDP-N-acetyl-D-glucosamine Biosynthesis II	0.364	0.167	NaN	UAP1
Adenine and Adenosine Salvage III	0.364	0.167	NaN	PNP
Purine Ribonucleosides Degradation to Ribose-1-phosphate	0.364	0.167	NaN	PNP
Glycogen Biosynthesis II (from UDP-D-Glucose)	0.364	0.167	NaN	GYS1
IL-15 Production	0.330	0.107	NaN	RELA,MAP3K11,PTK7

Ingenuity Canonical Pathways	-log(p-value)	Ratio	z-score	Proteins
Estrogen Biosynthesis	0.327	0.103	2.000	HSD17B10,POR,HSD17B12,CYP2S1
Thioredoxin Pathway	0.316	0.143	NaN	TXN
Phosphatidylcholine Biosynthesis I	0.316	0.143	NaN	PCYT1A
Inositol Pyrophosphates Biosynthesis	0.316	0.143	NaN	IPMK
Tryptophan Degradation to 2-amino-3-carboxymuconate Semialdehyde	0.316	0.143	NaN	KMO
Glycoaminoglycan-protein Linkage Region Biosynthesis	0.316	0.143	NaN	B3GAT3
Phototransduction Pathway	0.310	0.098	NaN	PRKACB,PRKAR2A,PRKACA,PRKAR1B,GUCY1B1
D-myo-inositol (1,4,5)-trisphosphate Degradation	0.309	0.111	NaN	SYNJ2,INPPL1
Fatty Acid α-oxidation	0.309	0.111	NaN	ALDH2,ALDH3A2
Calcium Signaling	0.285	0.091	-0.535	RAP2B,PRKACB,RAP1B,AKAP5,CAMK1D,MYL6,PRKAR2A,TPM1,TPM2,ATP2A2,RAP1A,MYL9,CACNA1F,NFAT5,PPP3CB,CAMKK1,PRKACA,PRKAR1B
Nur77 Signaling in T Lymphocytes	0.280	0.094	NaN	CASP3,PPP3CB,HLA-B,MAPK7,MAP3K2
Phosphatidylethanolamine Biosynthesis II	0.276	0.125	NaN	PCYT2
Salvage Pathways of Pyrimidine Deoxyribonucleotides	0.276	0.125	NaN	APOBEC3G
Sphingomyelin Metabolism	0.276	0.125	NaN	SMPD2
Role of IL-17F in Allergic Inflammatory Airway Diseases	0.276	0.095	1.000	RELA,TRAF3IP2,IL1B,RPS6KA5
Eicosanoid Signaling	0.270	0.092	NaN	PTGES,PTGS1,TBXAS1,GGT1,PAFAH1B1,PAFAH1B3
Unfolded protein response	0.266	0.093	NaN	SREBF2,OS9,CEBPB,HSPA2,CEBPG
Maturity Onset Diabetes of Young (MODY) Signaling	0.261	0.100	NaN	HNF1B,INS
Methionine Degradation I (to Homocysteine)	0.261	0.100	NaN	PRMT3,AHCYL2
Dermatan Sulfate Biosynthesis	0.253	0.091	-1.342	CHST7,CHPF,B3GAT3,CHST14,NDST1
Role of JAK2 in Hormone-like Cytokine Signaling	0.252	0.094	NaN	IRS1,IRS2,SIRPA
Endoplasmic Reticulum Stress Pathway	0.240	0.095	NaN	CASP3,CASP7
Glutathione Redox Reactions I	0.221	0.091	NaN	GPX1,GSTT1
Calcium Transport I	0.214	0.100	NaN	ATP2A2
Acyl-CoA Hydrolysis	0.190	0.091	NaN	ACOT8
Purine Nucleotides De Novo Biosynthesis II	0.190	0.091	NaN	IMPDH1
Basal Cell Carcinoma Signaling	0.000	0.087	-0.447	DVL2,WNT7A,DVL1,FZD6,FZD7,BMP1
Differential Regulation of Cytokine Production in Intestinal Epithelial Cells by IL-17A and IL-17F	0.000	0.087	NaN	LCN2,IL1B
nNOS Signaling in Neurons	0.000	0.087	NaN	CAPN5,PPP3CB,PRKCD,PFKM
TREM1 Signaling	0.000	0.086	-0.816	ITGB1,RELA,GRB2,ITGA5,IL1B,AKT3
Nucleotide Excision Repair Pathway	0.000	0.086	NaN	ERCC6,ERCC3,XPC
EIF2 Signaling	0.000	0.085	-1.069	RPL32,PABPC1,ATF3,PIK3C2A,GRB2,ACTB,FGFR1,AGO2,CCND1,RRAS2,IRS1,AGO3,INS,MRAS,AKT3,IRS2,PIK3R2,ATM
Phospholipases	0.000	0.085	-0.447	HMOX1,PLCB4,PAFAH1B1,PLD1,PAFAH1B3
Altered T Cell and B Cell Signaling in Rheumatoid Arthritis	0.000	0.084	NaN	RELA,TRAF3,SPP1,TGFB1,HLA-B,IL1B,CHUK
Lipid Antigen Presentation by CD1	0.000	0.083	NaN	AP1G2,PSAP
Assembly of RNA Polymerase III Complex	0.000	0.083	NaN	BRF2
Role of JAK1, JAK2 and TYK2 in Interferon Signaling	0.000	0.083	NaN	RELA,STAT2
Bupropion Degradation	0.000	0.083	NaN	POR,CYP2S1
Glutathione-mediated Detoxification	0.000	0.083	NaN	ANPEP,GSTT1
Glycogen Degradation II	0.000	0.083	NaN	PGM5
Mevalonate Pathway I	0.000	0.083	NaN	HMGCR
Pregnenolone Biosynthesis	0.000	0.083	NaN	MICAL3
BER pathway	0.000	0.083	NaN	PNKP
Sumoylation Pathway	0.000	0.083	2.121	RHOC,STUB1,DNMT3A,SIRT1,SENP3,RFC1,MAPK12,SNCA
Th2 Pathway	0.000	0.082	-0.905	TGFBR2,TGFBR1,PIK3C2A,GRB2,TGFB1,IRS1,FGFR1,HLA-B,PSEN2,IRS2,PIK3R2,ATM
Superpathway of Melatonin Degradation	0.000	0.081	2.236	POR,B3GAT3,MAOA,CYP2S1,IL4I1
Cell Cycle: G2/M DNA Damage Checkpoint Regulation	0.000	0.080	NaN	CDKN2A,YWHAH,ABL1,ATM
tRNA Charging	0.000	0.079	NaN	CARS,IARS2,IARS
Crosstalk between Dendritic Cells and Natural Killer Cells	0.000	0.079	NaN	RELA,ACTB,HLA-B,TNFSF10,TLN1,ACTG1,NECTIN2
Thyroid Cancer Signaling	0.000	0.077	NaN	RRAS2,MRAS,CCND1

Ingenuity Canonical Pathways	-log(p-value)	Ratio	z-score	Proteins
Role of IL-17A in Psoriasis	0.000	0.077	NaN	CCL20
Heparan Sulfate Biosynthesis	0.000	0.077	-0.447	PPME1,CHST7,CES2,B3GAT3,NDST1
Leukotriene Biosynthesis	0.000	0.077	NaN	GGT1
Bile Acid Biosynthesis, Neutral Pathway	0.000	0.077	NaN	SCP2
Th1 Pathway	0.000	0.077	-0.333	PIK3C2A,GRB2,IRS1,FGFR1,HLA-B,LGALS9,PSEN2,IRS2,PIK3R2,ATM
Chondroitin Sulfate Biosynthesis	0.000	0.076	-1.000	CHST7,CHPF,B3GAT3,NDST1
Transcriptional Regulatory Network in Embryonic Stem Cells	0.000	0.074	NaN	SET,CDYL,GATA6,L1CAM
Th1 and Th2 Activation Pathway	0.000	0.072	NaN	TGFBR2,TGFBR1,PIK3C2A,GRB2,TGFB1,IRS1,FGFR1,HLA-B,LGALS9,PSEN2,IRS2,PIK3R2,ATM
Cell Cycle Control of Chromosomal Replication	0.000	0.071	NaN	CDK17,CDK6,CDK9,CDK2
DNA Double-Strand Break Repair by Non-Homologous End Joining	0.000	0.071	NaN	ATM
Chondroitin Sulfate Degradation (Metazoa)	0.000	0.071	NaN	TMEM2
Glycogen Degradation III	0.000	0.071	NaN	PGM5
Dermatan Sulfate Biosynthesis (Late Stages)	0.000	0.071	NaN	CHST7,CHST14,NDST1
Methylglyoxal Degradation III	0.000	0.071	NaN	AKR1A1
Heparan Sulfate Biosynthesis (Late Stages)	0.000	0.069	-1.000	PPME1,CHST7,CES2,NDST1
Acetone Degradation I (to Methylglyoxal)	0.000	0.069	NaN	POR,CYP2S1
Role of Wnt/GSK-3β Signaling in the Pathogenesis of Influenza	0.000	0.069	-0.447	DVL2,WNT7A,DVL1,FZD6,FZD7
Calcium-induced T Lymphocyte Apoptosis	0.000	0.067	2.000	PPP3CB,PRKCD,HLA-B,ATP2A2
Chondroitin Sulfate Biosynthesis (Late Stages)	0.000	0.067	NaN	CHST7,CHPF,NDST1
Histidine Degradation VI	0.000	0.067	NaN	MICAL3
Neuroprotective Role of THOP1 in Alzheimer's Disease	0.000	0.063	1.633	PRKACB,HGFAC,DPP4,HLA-B,PRKAR2A,PRKACA,PRKAR1B
Nicotine Degradation III	0.000	0.063	NaN	POR,B3GAT3,CYP2S1
Assembly of RNA Polymerase II Complex	0.000	0.060	NaN	GTF2A2,ERCC3,GTF2A1
RAN Signaling	0.000	0.059	NaN	KPNA1
Oxidative Phosphorylation	0.000	0.058	-0.816	VPS9D1,ATP5PF,UQCRH,COX5B,CYB5A,COX15
Differential Regulation of Cytokine Production in Macrophages and T Helper Cells by IL-17A and IL-17F	0.000	0.056	NaN	IL1B
Nicotine Degradation II	0.000	0.056	NaN	POR,B3GAT3,CYP2S1
Valine Degradation I	0.000	0.056	NaN	ACADSB
Complement System	0.000	0.056	NaN	C3,CFB
Notch Signaling	0.000	0.054	NaN	PSEN2,NUMBL
Cardiomyocyte Differentiation via BMP Receptors	0.000	0.053	NaN	MAP3K7
Ubiquinol-10 Biosynthesis (Eukaryotic)	0.000	0.053	NaN	MICAL3
Melatonin Degradation I	0.000	0.053	NaN	POR,B3GAT3,CYP2S1
Retinol Biosynthesis	0.000	0.053	NaN	DDHD2,CES2
Primary Immunodeficiency Signaling	0.000	0.046	NaN	TAP1,TAP2
Graft-versus-Host Disease Signaling	0.000	0.046	NaN	HLA-B,IL1B
GABA Receptor Signaling	0.000	0.043	NaN	CACNA1F,AP1G2,MRAS,ADCY6
Role of Cytokines in Mediating Communication between Immune Cells	0.000	0.039	NaN	TGFB1,IL1B
B Cell Development	0.000	0.035	NaN	HLA-B
Thyroid Hormone Metabolism II (via Conjugation and/or Degradation)	0.000	0.028	NaN	B3GAT3
nNOS Signaling in Skeletal Muscle Cells	0.000	0.025	NaN	CACNA1F
Role of Hypercytokinemia/hyperchemokinemia in the Pathogenesis of Influenza	0.000	0.024	NaN	IL1B
Autoimmune Thyroid Disease Signaling	0.000	0.024	NaN	HLA-B
Communication between Innate and Adaptive Immune Cells	0.000	0.023	NaN	HLA-B,IL1B
Allograft Rejection Signaling	0.000	0.021	NaN	HLA-B

Ratio: items found divided total pathway items. NaN: indicates those pathways where no activity pattern was present. Positive and negative z-scores indicate up- or downregulation of the pathways, 0 indicates no change.

Supplemental Table 3. Validation of proteomic data with transcriptomic datasets

				HPOD Proteomics, scr vs CKAP4 KD			*mRNA, DKD vs healthy (Levin et al)*			*mRNA DKD vs healthy (Woroniecka et al)*			*mRNA, DKD vs healthy (Ju et al)*		
**Protein**	**Protein name(s)**	**Protein class**	**Function**	**fold change**	**adjusted p value**		**fold change**	**adjusted p value**		**fold change**	**adjusted p value**		**fold change**	**adjusted p value**	
**CKAP4**	Cytoskeleton-associated protein 4 (Climp-63, p63)	CKAP4	Cytoskeleton linker	0.426	2.04E-05	***	0.713	1.01E-03	***	0.284	9.05E-04	***	0.494	8.51E-01	ns
CDC42	Cell division control protein 42 homolog	small GTPase	mainly filopodia formation	0.925	1.87E-03	**	1.105	4.86E-01	ns	0.338	6.70E-02	ns	0.495	6.94E-02	ns
RAC1	Ras-related C3 botulinum toxin substrate 1	small GTPase	mainly lamellipodia formation	0.784	1.46E-03	**	0.795	6.31E-02	ns	0.326	4.97E-04	***	0.483	2.91E-01	ns
RAC2	Ras-related C3 botulinum toxin substrate 2	small GTPase	mainly lamellipodia formation	0.759	4.17E-04	****	2.341	6.03E-05	***	5.077	1.90E-02	*	6.169	3.45E-06	***
RAC3	Ras-related C3 botulinum toxin substrate 3	small GTPase	mainly lamellipodia formation	1.089	6.17E-01	ns	0.999	9.99E-01	ns	0.477	2.43E-01	ns	0.460	3.00E-03	**
RAP1A	Ras-related protein Rap-1A	small GTPase	integrin activation and clustering	0.765	2.82E-02	*	1.147	2.84E-01	ns	0.467	4.78E-01	ns	0.472	5.25E-01	ns
RAP1B	Ras-related protein Rap-1B	small GTPase	integrin activation and clustering	0.675	9.87E-06	***	1.041	7.68E-01	ns	0.492	8.79E-01	ns			
RAP2A	Ras-related protein Rap-2A	small GTPase	integrin activation and clustering	0.882	1.65E-01	ns	1.071	7.27E-01	ns	0.428	1.50E-02	*	0.486	5.89E-01	ns
RAP2B	Ras-related protein Rap-2B	small GTPase	integrin activation and clustering	0.717	1.20E-04	***	1.160	4.54E-01	ns	2.757	2.70E-02	*	2.369	1.00E-03	***
RHOA	Transforming protein RhoA	small GTPase	stress fibers formation and focal adhesion stability	1.002	7.27E-01	ns	1.139	1.22E-01	ns	0.493	8.96E-01	ns	2.099	1.30E-01	ns
RHOB	Rho-related GTP-binding protein RhoB	small GTPase	stress fibers formation and focal adhesion stability	1.000	9.86E-01	ns	0.403	8.18E-06	***	0.443	2.47E-01	ns	0.415	6.10E-02	ns
RHOBTB1	Rho-related BTB domain-containing protein 1	small GTPase	Rho protein stability				1.657	1.07E-03	**	2.864	6.30E-02	ns	3.110	2.10E-02	*
RHOBTB2	Rho-related BTB domain-containing protein 2	small GTPase	Rho protein stability				0.514	1.13E-07	***	0.346	2.00E-03	**	0.300	1.21E-04	***
RHOC	Rho-related GTP-binding protein RhoC	small GTPase	stress fibers formation and focal adhesion stability	0.685	2.78E-02	*	1.088	6.32E-01	ns				3.154	8.05E-04	***
RHOD	Rho-related GTP-binding protein RhoD	small GTPase	vesicle transport, filopodia formation	0.973	7.52E-01	ns	1.333	1.91E-01	ns	2.400	3.60E-02	*	2.196	2.00E-03	**
RHOF	Rho-related GTP-binding protein RhoF (RIF)	small GTPase	Vesicle transport, filopodia formation	1.182	3.61E-02	*	0.874	1.33E-01	ns	2.278	1.91E-01	ns	0.459	3.05E-01	ns
RHOG	Rho-related GTP-binding protein RhoG	small GTPase	mainly lamellipodia formation	0.806	1.57E-03	**	1.214	1.92E-01	ns	0.486	7.50E-01	ns	2.634	1.40E-04	***
RHOH	Rho-related GTP-binding protein RhoH	small GTPase	not cytoskeleton related, immune cell function				2.067	5.69E-02	ns	3.394	5.00E-03	**	2.118	5.26E-01	ns
RHOJ	Ras homolog family member J (TCL)	small GTPase	mainly filopodia formation				0.888	6.68E-01	ns						
RHOQ	Rho-related GTP-binding protein RhoQ (TC10)	small GTPase	mainly filopodia formation				0.639	1.30E-04	***	0.324	4.49E-04	***	0.467	5.06E-01	ns
RHOU	Rho-related GTP-binding protein RhoU (WRCH)	small GTPase	filopodia and lamellipodia formation				0.448	5.81E-07	***						
RND1	Rho-related GTP-binding protein Rho6	small GTPase	stress fibers destruction and focal adhesion instability				0.531	5.09E-02	ns	0.460	3.02E-01	ns	0.454	3.16E-01	ns
RND2	Rho-related GTP-binding protein RhoN	small GTPase	stress fibers destruction and focal adhesion instability				0.979	9.65E-01	ns	0.486	4.24E-01	ns	0.477	1.52E-01	ns
RND3	Rho-related GTP-binding protein RhoE	small GTPase	stress fibers destruction and focal adhesion instability	1.075	3.54E-02	*	0.829	5.35E-01	ns	2.227	5.23E-01	ns	0.478	6.35E-01	ns
ARHGAP1	Rho GTPase Activating Protein 1	small GTPases GAP	deactivation of small GTPases (GTP→GDP)	0.734	1.43E-04	***	1.335	4.74E-03	**	2.208	5.50E-02	ns	2.073	1.93E-01	ns
ARHGAP5	Rho GTPase Activating Protein 5	small GTPases GAP	deactivation of small GTPases (GTP→GDP)	0.845	2.55E-04	***	0.843	7.76E-02	ns	0.448	4.52E-01	ns	0.316	6.41E-05	***
ARHGAP6	Rho GTPase Activating Protein 6	small GTPases GAP	deactivation of small GTPases (GTP→GDP)	0.826	4.36E-02	*	1.395	1.72E-01	ns	2.144	3.17E-01	ns	2.049	5.42E-01	ns
ARHGAP9	Rho GTPase Activating Protein 9	small GTPases GAP	deactivation of small GTPases (GTP→GDP)	0.813	1.49E-03	**	1.646	1.52E-01	ns						
ARHGAP10	Rho GTPase Activating Protein 10	small GTPases GAP	deactivation of small GTPases (GTP→GDP)	1.076	3.32E-02	*	1.008	9.81E-01	ns	2.320	7.10E-02	ns	2.049	6.77E-01	ns
ARHGAP15	Rho GTPase Activating Protein 15	small GTPases GAP	deactivation of small GTPases (GTP→GDP)				2.360	2.73E-04	***	4.383	4.00E-03	**	3.782	2.00E-03	**
ARHGAP17	Rho GTPase Activating Protein 17	small GTPases GAP	deactivation of small GTPases (GTP→GDP)	1.111	1.85E-03	**	1.059	7.02E-01	ns	0.421	8.00E-02	ns	2.235	1.40E-02	*
ARHGAP18	Rho GTPase Activating Protein 18	small GTPases GAP	deactivation of small GTPases (GTP→GDP)	0.586	1.39E-05	***	0.904	5.28E-01	ns						
ARHGAP19	Rho GTPase Activating Protein 19	small GTPases GAP	deactivation of small GTPases (GTP→GDP)				0.416	1.39E-06	***	0.012	4.01E-06	***	0.251	5.27E-04	***
ARHGAP21	Rho GTPase Activating Protein 21	small GTPases GAP	deactivation of small GTPases (GTP→GDP)	1.046	2.29E-01	ns	0.617	7.60E-07	***						
ARHGAP22	Rho GTPase Activating Protein 22	small GTPases GAP	deactivation of small GTPases (GTP→GDP)							2.336	5.30E-02	ns	2.231	1.03E-01	ns
ARHGAP23	Rho GTPase Activating Protein 23	small GTPases GAP	deactivation of small GTPases (GTP→GDP)	0.745	4.74E-02	*	0.447	6.12E-03	**						
ARHGAP24	Rho GTPase Activating Protein 24	small GTPases GAP	deactivation of small GTPases (GTP→GDP)				1.271	2.79E-01	ns	0.477	5.10E-01	ns	0.420	6.00E-03	**
ARHGAP26	Rho GTPase Activating Protein 26	small GTPases GAP	deactivation of small GTPases (GTP→GDP)	1.110	8.15E-03	**	0.905	6.87E-01	ns	2.272	1.07E-01	ns	2.099	6.50E-02	ns
ARHGAP27	Rho GTPase Activating Protein 27	small GTPases GAP	deactivation of small GTPases (GTP→GDP)	1.241	5.28E-02	ns	1.419	2.39E-01	ns						
ARHGAP28	Rho GTPase Activating Protein 28	small GTPases GAP	deactivation of small GTPases (GTP→GDP)				0.515	3.36E-03	**	0.069	6.76E-06	***	0.464	5.81E-01	ns
ARHGAP29	Rho GTPase Activating Protein 29	small GTPases GAP	deactivation of small GTPases (GTP→GDP)	0.656	9.68E-06	***	0.774	5.21E-02	ns	0.289	2.00E-03	**	0.484	6.97E-01	ns
ARHGAP30	Rho GTPase Activating Protein 30	small GTPases GAP	deactivation of small GTPases (GTP→GDP)				2.080	5.17E-03	**						
ARHGAP32	Rho GTPase Activating Protein 32	small GTPases GAP	deactivation of small GTPases (GTP→GDP)				0.906	7.90E-01	ns	0.466	3.80E-01	ns	0.409	5.28E-05	***
ARHGAP33	Rho GTPase Activating Protein 33	small GTPases GAP	deactivation of small GTPases (GTP→GDP)				0.367	5.18E-04	***	2.218	2.43E-01	ns	0.457	7.00E-03	**
ARHGAP35	Rho GTPase Activating Protein 35 (GLFR1)	small GTPases GAP	deactivation of small GTPases (GTP→GDP)	1.057	3.37E-02	*	0.919	4.78E-01	ns	2.418	1.30E-02	*	0.433	1.92E-06	***
ARHGAP42	Rho GTPase Activating Protein 42	small GTPases GAP	deactivation of small GTPases (GTP→GDP)	1.016	5.28E-01	ns	1.298	3.65E-01	ns						
RACGAP1	Rac GTPase Activating Protein 1	small GTPases GAP	deactivation of small GTPases (GTP→GDP)				1.477	9.90E-02	ns	2.011	9.54E-01	ns	2.435	7.50E-02	ns
ARHGDIA	Rho GDP Dissociation Inhibitor Alpha	small GTPases GDI	deactivation of small GTPases (GDP)	0.839	1.45E-03	**	1.033	8.81E-01	ns	0.465	5.44E-01	ns	0.477	2.16E-01	ns
ARHGDIB	Rho GDP Dissociation Inhibitor Beta	small GTPases GDI	deactivation of small GTPases (GDP)				1.068	7.98E-01	ns	2.326	3.79E-01	ns	2.590	1.00E-03	***
GDI1	GDP Dissociation Inhibitor 1	small GTPases GDI	deactivation of small GTPases (GDP)	1.126	1.36E-03	**									
GDI2	GDP Dissociation Inhibitor 2	small GTPases GDI	deactivation of small GTPases (GDP)	0.885	1.63E-05	***	1.302	3.20E-03	**	2.547	3.00E-03	**	2.108	2.43E-01	ns
ARHGEF1	Rho Guanine Nucleotide Exchange Factor 1	small GTPases GEF	activation of small GTPases (GDP→GTP)	0.880	6.73E-04		1.103	6.18E-01	ns	0.459	3.07E-01	ns	0.486	3.74E-01	ns
ARHGEF2	Rho Guanine Nucleotide Exchange Factor 2	small GTPases GEF	activation of small GTPases (GDP→GTP)	1.199	2.43E-03	**	0.990	9.74E-01	ns	2.107	5.34E-01	ns	2.152	2.10E-02	*
ARHGEF5	Rho Guanine Nucleotide Exchange Factor 5	small GTPases GEF	activation of small GTPases (GDP→GTP)	0.855	7.22E-03	**	0.956	8.95E-01	ns	0.489	7.57E-01	ns	0.474	3.18E-01	ns
ARHGEF6	Rho Guanine Nucleotide Exchange Factor 6	small GTPases GEF	activation of small GTPases (GDP→GTP)	0.899	6.92E-02	ns	1.371	7.63E-02	ns	0.488	9.00E+00	ns	2.990	1.23E-05	***
ARHGEF7	Rho Guanine Nucleotide Exchange Factor 7 (BETA PIX)	small GTPases GEF	activation of small GTPases (GDP→GTP)	0.738	2.86E-04	***	0.997	9.91E-01	ns	0.467	3.65E-01	ns	0.492	6.65E-01	ns
ARHGEF10	Rho Guanine Nucleotide Exchange Factor 10	small GTPases GEF	activation of small GTPases (GDP→GTP)	0.955	9.34E-02	ns	0.626	3.16E-02	*	0.125	9.15E-09	***	0.427	2.00E-03	**
ARHGEF10L	Rho Guanine Nucleotide Exchange Factor 10 like	small GTPases GEF	activation of small GTPases (GDP→GTP)	1.203	6.58E-03	**	1.303	1.63E-01	ns	2.123	4.03E-01	ns	0.494	7.71E-01	ns
ARHGEF11	Rho Guanine Nucleotide Exchange Factor 11	small GTPases GEF	activation of small GTPases (GDP→GTP)	1.077	3.67E-01	ns	0.717	8.99E-03	**	0.446	2.50E-02	*	0.456	1.60E-02	*
ARHGEF12	Rho Guanine Nucleotide Exchange Factor 12	small GTPases GEF	activation of small GTPases (GDP→GTP)	0.930	1.13E-02	*	0.645	2.27E-05	***	0.057	5.58E-08	***	0.410	2.60E-02	*
ARHGEF17	Rho Guanine Nucleotide Exchange Factor 17	small GTPases GEF	activation of small GTPases (GDP→GTP)	1.005	8.90E-01	ns	0.614	6.14E-04	***	0.408	2.80E-02	*	0.448	7.30E-02	ns
ARHGEF18	Rho Guanine Nucleotide Exchange Factor 18	small GTPases GEF	activation of small GTPases (GDP→GTP)	1.108	6.80E-03	**	0.732	2.29E-01	ns	0.345	3.00E-03	**	0.401	4.00E-03	***
ARHGEF26	Rho Guanine Nucleotide Exchange Factor 26	small GTPases GEF	activation of small GTPases (GDP→GTP)				0.321	1.07E-05	***						
ARHGEF28	Rho Guanine Nucleotide Exchange Factor 28	small GTPases GEF	activation of small GTPases (GDP→GTP)	0.793	1.81E-03	**	1.147	5.01E-01	ns						
ARHGEF37	Rho Guanine Nucleotide Exchange Factor 37	small GTPases GEF	activation of small GTPases (GDP→GTP)				0.382	9.59E-08	***						
ARHGEF40	Rho Guanine Nucleotide Exchange Factor 40	small GTPases GEF	activation of small GTPases (GDP→GTP)				0.685	6.11E-04	***						
RAP1GDS1	Rap1 GTPase-GDP Dissociation Stimulator 1	small GTPases GEF	activation of small GTPases (GDP→GTP)				1.058	6.93E-01	ns	0.395	2.50E-02	*	2.147	1.76E-01	ns
RAPGEF2	Rap Guanine Nucleotide Exchange Factor 2	small GTPases GEF	activation of small GTPases (GDP→GTP)	0.996	9.65E-01	ns	0.788	6.82E-02	ns	0.390	2.10E-02	*	0.464	3.40E-02	*
RAPGEF3	Rap Guanine Nucleotide Exchange Factor 3	small GTPases GEF	activation of small GTPases (GDP→GTP)				0.678	8.80E-04	***	0.238	2.87E-05	***	0.432	1.96E-01	ns
RAPGEF6	Rap Guanine Nucleotide Exchange Factor 6	small GTPases GEF	activation of small GTPases (GDP→GTP)	0.549	2.33E-03	**	0.904	6.79E-01	ns	0.441	2.91E-01	ns	2.014	9.07E-01	ns
ITGA1	Integrin alpha-1	Integrin	laminin and collagen receptor	0.613	1.85E-04	***	0.978	9.21E-01	ns	0.331	7.00E-03	**			
ITGA2	Integrin alpha-2	Integrin	collagen receptor	0.922	1.82E-03	**	1.481	1.52E-02	*	2.341	8.00E-02	ns	0.490	6.61E-01	
ITGA3	Integrin alpha-3	Integrin	adhesion to GMB	0.586	6.22E-06	***	0.506	6.68E-06	***	0.175	1.23E-05	***	0.468	5.24E-01	
ITGA4	Integrin alpha-4	Integrin	cell-cell adhesion				2.436	2.63E-04	***	2.111	9.20E-02	ns	2.936	2.00E-03	***
ITGA5	Integrin alpha-5	Integrin	fibronection receptor	0.800	8.18E-04	***	1.118	6.69E-01	ns	0.485	6.81E-01	ns	2.342	3.00E-03	**
ITGA6	Integrin alpha-6	Integrin	laminin receptor (ITGA6/B4)	1.053	2.22E-01	ns	1.341	4.61E-02	*	2.239	3.84E-01	ns	0.446	2.80E-02	*
ITGA8	Integrin alpha-8	Integrin	fibronectin receptor (ITGAV/A8)				0.863	6.04E-01	ns	0.145	9.45E-04	***	0.487	8.39E-01	ns
ITGA11	Integrin alpha-11	Integrin	collagen receptor	1.620	1.73E-02	*	1.303	3.57E-01	ns						
ITGAL	Integrin alpha-L	Integrin	receptor for ICAM				2.043	5.41E-03	**				2.526	5.74E-04	***
ITGAV	Integrin alpha-V	Integrin	fibronectin/laminin receptor (ITGAV/B3)	0.498	2.40E-05	***	0.729	1.28E-01	ns	0.284	3.53E-04	***	0.449	2.31E-01	ns
ITGB1	Integrin beta-1	Integrin	fibronectin receptor	0.361	4.46E-06	***	1.098	6.76E-01	ns	0.408	6.00E-02	ns	0.471	3.00E-03	**
ITGB2	Integrin beta-2	Integrin	receptor for ICAM				2.096	5.69E-04	***	5.704	2.00E-03	**	10.666	1.59E-05	***

Protein	Protein name(s)	Protein class	Function	HPOD Proteomics, scr vs CKAP4 KD			mRNA, DKD vs healthy (Levin et al)			mRNA DKD vs healthy (Woroniecka et al)			mRNA, DKD vs healthy (Ju et al)		
				fold change	adjusted p value		fold change	adjusted p value		fold change	adjusted p value		fold change	adjusted p value	
ITGB3	Integrin beta-3	Integrin	fibronectin receptor (ITGAV/B3)	0.384	4.26E-06	***	1.172	6.26E-01	ns	0.355	**	ns	2.062	5.52E-01	ns
ITGB4	Integrin beta-4	Integrin	laminin receptor (ITGA6/B4)	1.155	2.70E-04	***	2.575	2.27E-05	***	2.328	1.80E-02	*	0.486	1.17E-01	ns
ITGB5	Integrin beta-5	Integrin	fibronectin receptor	0.629	1.64E-04	***	0.499	5.33E-07	***	0.237	1.42E-05	***	0.486	6.93E-01	ns
ITGB6	Integrin beta-6	Integrin	fibrillin receptor (ITGAV/B6)				2.772	1.65E-03	**	2.474	1.10E-02	*	2.805	2.70E-02	*
ITGB8	Integrin beta-8	Integrin	fibronectin receptor	1.267	7.52E-03	**	0.833	4.85E-01	ns	0.449	5.52E-04	***	0.152	3.16E-01	ns
FERMT2	Fermitin family homolog 2 (Kindlin-2)	Integrin modulator	scaffolder for TLN	0.953	1.00E-04	***	0.696	6.22E-05	***	0.147	5.18E-07	***	0.347	6.08E-04	***
FERMT3	Fermitin family homolog 3 (Kindlin-3)	Integrin modulator	scaffolder for TLN	1.051	4.21E-01	ns	1.290	3.41E-01	ns						
ILK	Integrin-linked protein kinase	Integrin modulator	integrin modulation, growth factors	0.946	2.67E-02	*	1.032	8.88E-01	ns	0.450	5.33E-01	ns	2.088	3.18E-01	ns
ILKAP	Integrin-linked kinase-associated serine/threonine phosphatase 2C	Integrin modulator	integrin modulation, growth factors	1.087	7.23E-02	ns	0.818	5.99E-02	ns	0.446	1.58E-01	ns	0.443	1.62E-01	ns
ITGB1BP1	Integrin beta-1-binding protein 1 (ICAP1)	Integrin modulator	inhibits ITGB1 clustering	1.306	3.06E-02	**	1.003	9.89E-01	ns	0.425	3.01E-01	ns	2.142	3.20E-01	ns
PTK2	Focal adhesion kinase 1 (FAK)	Integrin modulator	activation of PXN	0.876	2.02E-04	***	1.134	2.95E-01	ns	0.300	1.40E-04	***	0.438	7.00E-03	**
PXN	Paxillin	Integrin modulator	integrin/ECM signal transduction	0.834	4.54E-03	**	1.020	9.17E-01	ns	2.233	1.00E-02	**	0.465	1.20E-02	*
SRC	Proto-oncogene tyrosine-protein kinase Src	Integrin/actin modulator	RAP signalling, FAK activation, actin polymerization	0.636	3.01E-06	***	1.314	1.80E-01	ns	2.239	2.40E-02	*	0.470	1.30E-02	*
TLN1	Talin 1	Integrin/actin modulator	actin/integrin interaction	0.466	5.13E-06	***	0.839	1.93E-01	ns	2.081	5.12E-01	ns	0.428	2.70E-02	*
TLN2	Talin 2	Integrin/actin modulator	actin/integrin interaction	0.843	2.63E-04	***	1.252	4.20E-01	ns	0.401	2.32E-01	ns	0.334	3.10E-02	*
VCL	Vinculin	Integrin/actin modulator	actin/integrin interaction	0.793	7.68E-05	***	1.014	9.48E-01	ns	0.307	1.40E-02	**	0.495	7.75E-01	
CFL1	Cofilin-1	Actin modulator	Binds and depolymerize F-actin	0.603	1.11E-06	***	1.254	1.62E-03	**	0.461	1.77E-01		2.275	1.00E-03	***
CFL2	Cofilin-2	Actin modulator	Binds and depolymerize F-actin	0.640	5.24E-04	***	0.998	9.94E-01	ns						
PFN1	Profilin-1	Actin modulator	actin filaments polymerization	1.281	6.28E-02	ns	1.293	1.16E-02	*	5.326	2.00E-02	*	2.359	2.18E-01	ns
PFN2	Profilin-2	Actin modulator	actin filaments polymerization	0.979	5.78E-01	ns	0.863	3.33E-01	ns	0.422	8.80E-02	ns	0.455	8.30E-02	ns
PLEC	Plectin	Actin/microtubules modulator	crosslinker actin/microtubules/intermediate filaments	0.960	9.57E-02	ns	0.690	2.67E-04	***	0.486	7.05E-01	ns	0.453	9.33E-04	***
APC	Adenomatous polyposis coli protein	Microtubules modulator	centrosome modulator	1.306	4.70E-01	ns	0.859	3.17E-01	ns	0.255	5.81E-04	***	0.413	6.16E-04	***
CLASP1	Cytoplasmic linker associated protein 1	Microtubules modulator	promotes nucleation (+TIP)	0.703	2.50E-06	***	1.121	6.30E-01	ns				0.456	2.00E-02	*
CLASP2	Cytoplasmic linker associated protein 2	Microtubules modulator	promotes nucleation (+TIP)	0.946	1.23E-01	ns	0.964	8.26E-01	ns	0.227	6.00E-03	**	0.418	3.00E-03	**
DPYSL2	Dihydropyrimidinase-related protein 2	Microtubules modulators	enhances microtubules assembly	1.330	1.40E-04	***	0.941	7.87E-01	ns	0.381	3.40E-02	*	0.406	1.00E-03	***
DNAH17	Dynein axonemal heavy chain 17	Microtubules modulators	microtubuli associated motor protein				0.484	5.41E-03	**	0.482	1.20E-02	*	0.467	3.31E-01	ns
DNAH9	Dynein axonemal heavy chain 9	Microtubules modulators	microtubuli associated motor protein				0.388	1.79E-04	***	0.466	2.79E-01	ns	0.450	5.00E-03	**
DST	Dystonin 1	Microtubules modulators	crosslinker actin/microtubules	0.791	2.23E-04	***	0.582	1.15E-06	***	0.201	9.88E-07	***	2.301	6.00E-03	**
DYNC1H1	Cytoplasmic dynein 1 heavy chain 1	Microtubules modulators	intracellular motility via microtubules	0.996	8.15E-01	ns	1.017	9.39E-01	ns	2.169	6.73E-01	ns	0.450	4.70E-02	*
DYNC2H1	Cytoplasmic dynein 2 heavy chain 1	Microtubules modulators	intracellular motility via microtubules	1.071	5.77E-01	ns	0.718	6.97E-05	***				0.393	5.96E-04	***
KATNA1	Katanin 1	Microtubules modulators	promotes microtubules disassembly	0.739	3.20E-03	**	1.119	4.87E-01	ns	2.032	8.58E-01	ns	2.255	4.00E-04	**
MACF1	Microtubule –actin crosslinking factors 1	Microtubules modulators	crosslinker actin/microtubules	0.788	1.03E-04	***	0.880	3.33E-01	ns	0.353	8.00E-03	**	0.478	4.16E-01	ns
MAP1A	Microtubules associated protein 1A	Microtubules modulators	microtubules stabilizer	0.632	6.00E-04	***	0.569	3.72E-04	***	0.362	7.39E-04	***	0.438	5.72E-04	***
MAP1B	Microtubules associated protein 1B	Microtubules modulators	microtubules stabilizer	0.937	1.70E-01	ns	0.683	5.29E-02	ns	0.107	2.19E-04	***			
MAP2	Microtubules associated protein 2	Microtubules modulators	microtubules stabilizer				0.788	3.05E-01	ns	0.497	9.10E-01	ns	0.471	3.96E-01	ns
MAP6	Microtubules associated protein 6	Microtubules modulators	microtubules stabilizer				0.432	9.90E-05	***						
MAPT	Microtubules associated protein Tau	Microtubules modulators	microtubules stabilizer				1.100	7.81E-01	ns	0.475	2.72E-01	ns	0.361	3.43E-04	***
MARK4	Microtubule Affinity Regulating Kinase 4	Microtubules modulators	microtubules organization	1.020	7.71E-01	ns	0.619	1.72E-03	**	0.361	1.20E-02	*	0.480	3.13E-01	ns
KIF2C	Kinesin family member 2C (MCKA)	Microtubules modulators	microtubules depolymerization (+TIP)							2.352	8.00E-02	ns	2.462	7.00E-03	**
TUBG1	Tubulin gamma-1 chain (Gamma-1-tubulin) (GCP-1)	g-TuRC complex	microtubules nucleation	0.979	4.04E-01	ns	1.110	5.32E-01	ns	2.293	4.81E-01	ns	2.249	1.79E-01	ns
TUBG2	Tubulin gamma-2 chain (Gamma-2-tubulin)	g-TuRC complex	microtubules nucleation	0.883	5.65E-02	ns	0.906	5.55E-01	ns	0.473	5.12E-01	ns	0.462	7.00E-02	ns
TUBGCP2	Gamma-tubulin complex component 2	g-TuRC complex	microtubules nucleation	1.097	3.26E-02	*	0.969	8.62E-01	ns	2.301	3.90E-02	*	0.457	3.00E-03	**
TUBGCP3	Gamma-tubulin complex component 3	g-TuRC complex	microtubules nucleation	1.128	2.19E-02	*	1.028	9.18E-01	ns	0.328	4.00E-03	**	0.437	3.00E-03	**
TUBGCP4	Gamma-tubulin complex component 4	g-TuRC complex	microtubules nucleation	0.953	3.36E-01	ns	1.122	7.03E-01	ns	0.466	2.02E-01	ns	2.045	6.90E-01	ns
TUBGCP5	Gamma-tubulin complex component 5	g-TuRC complex	microtubules nucleation	1.223	3.28E-02	*	1.082	7.68E-01	ns	2.320	2.57E-01	ns	2.015	7.70E-01	ns
TUBGCP6	Gamma-tubulin complex component 6	g-TuRC complex	microtubules nucleation	1.147	7.67E-02	ns	1.047	8.66E-01	ns						
TUBA1A	Tubulin alpha-1A chain	tubulin	microtubules				1.388	2.16E-02	*	2.267	1.38E-01	ns	2.979	5.86E-05	***
TUBA1B	Tubulin alpha-1B chain	tubulin	microtubules	0.820	3.02E-03	**	1.393	1.18E-01	ns	2.981	7.60E-02	ns	2.390	1.27E-04	***
TUBA1C	Tubulin alpha-1C chain	tubulin	microtubules	0.869	8.00E-03	**	1.150	6.32E-01	ns	2.959	1.65E-01	ns	2.626	2.62E-06	***
TUBA3C	Tubulin alpha-3C chain	tubulin	microtubules							2.715	1.20E-02	*	2.193	3.70E-02	*
TUBA3D	Tubulin alpha-3D chain	tubulin	microtubules							2.912	9.00E-03	**			
TUBA4A	Tubulin alpha-4A chain	tubulin	microtubules	0.760	1.94E-03	**	1.403	3.37E-02	*	2.882	4.50E-02	*	2.868	2.29E-04	***
TUBA8	Tubulin alpha-8 chain (alpha chain like 2) (TUBAL2)	tubulin	microtubules							2.089	4.10E-01	ns	0.492	5.44E-01	ns
TUBAL3	Tubulin alpha chain-like 3	tubulin	microtubules	0.815	3.53E-01	ns				2.283	4.27E-01	ns	0.349	2.20E-02	*
TUBB	Tubulin beta chain	tubulin	microtubules	0.855	7.75E-05	***	1.832	4.53E-03	**	2.576	3.13E-01	ns	2.514	3.00E-03	**
TUBB2A	Tubulin beta-2A chain	tubulin	microtubules	0.925	1.68E-01	ns	0.663	6.75E-03	**	0.328	2.80E-02	*	2.161	3.12E-01	ns
TUBB2B	Tubulin beta-2B chain	tubulin	microtubules	1.133	1.89E-01	ns	1.327	3.47E-01	ns	3.145	2.00E-02	*	4.287	1.00E-03	***
TUBB2C	Tubulin beta-2C chain	tubulin	microtubules							2.170	6.99E-01	ns	2.069	4.69E-01	ns
TUBB3	Tubulin beta-3 chain	tubulin	microtubules	0.894	5.27E-01	ns				2.086	8.19E-01	ns	2.717	9.77E-05	***
TUBB4A	Tubulin beta-4A chain	tubulin	microtubules	0.768	2.55E-03	**	1.989	5.48E-02	ns						
TUBB4B	Tubulin beta-4B chain	tubulin	microtubules	0.951	7.51E-02	ns	1.024	9.21E-01	ns						
TUBB6	Tubulin beta-6 chain	tubulin	microtubules	0.834	5.26E-04	***	0.966	8.66E-01	ns	0.322	1.00E-02	**	3.104	4.61E-06	***
TUBD1	Tubulin delta chain	tubulin	microtubules, centriole replication				0.675	3.32E-02	*	0.457	1.61E-01	ns	0.480	2.67E-01	ns
TUBE1	Tubulin epsilon chain	tubulin	microtubules, centriole replication				0.904483079	0.65050							

Significative p <0.05 are highlighted in green. Upregulation (unlogged FC>20%) in blue, whereas downregulation (unlogged FC<20%) in red. Functions from data mining (Uniprot). Abbreviations used: GAP = GTPase activating protein, GEF = guanine nucleotide exchange factor, GDI = GTP dissociation inhibitor, + TIP = plus end tracking protein (accretion side of microtubules), GBM = glomerular basement membrane, ECM = extracellular matrix.
