# Supplementary material for: Cytoskeleton-associated protein 4 affects podocyte cytoskeleton dynamics in diabetic kidney disease
Source: JCI Insight. 2025 Jun 10;10(14):e181298. doi: 10.1172/jci.insight.181298 (PMC12288980; doi:10.1172/jci.insight.181298)

Full unedited blots for Figure 1E

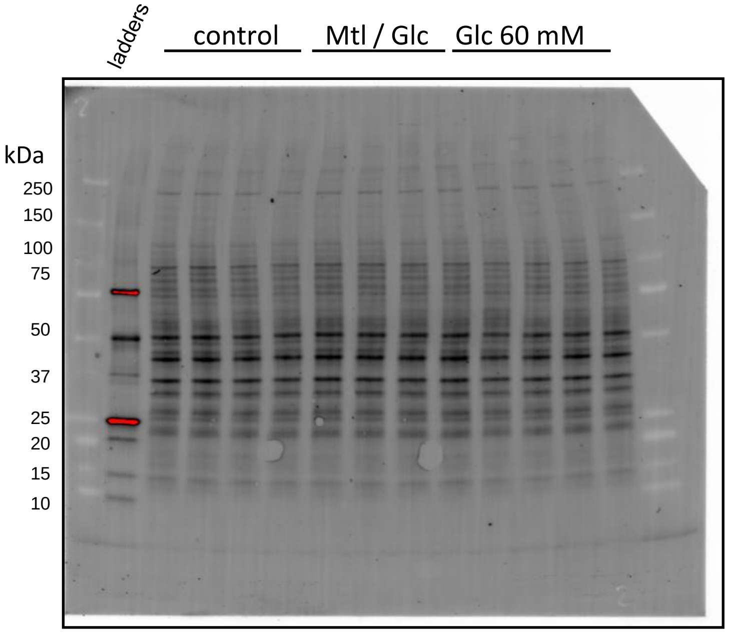

Total protein blot

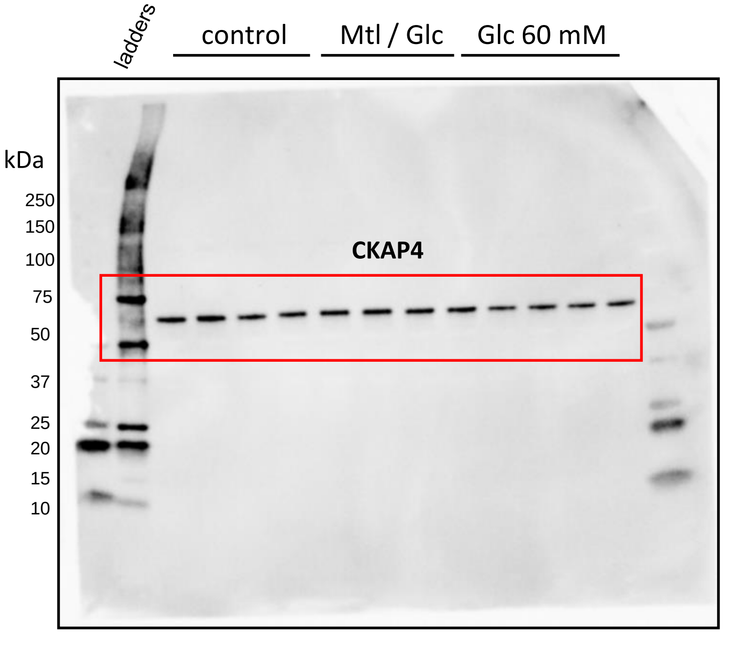

Full unedited blots for Figure 1F, part 1

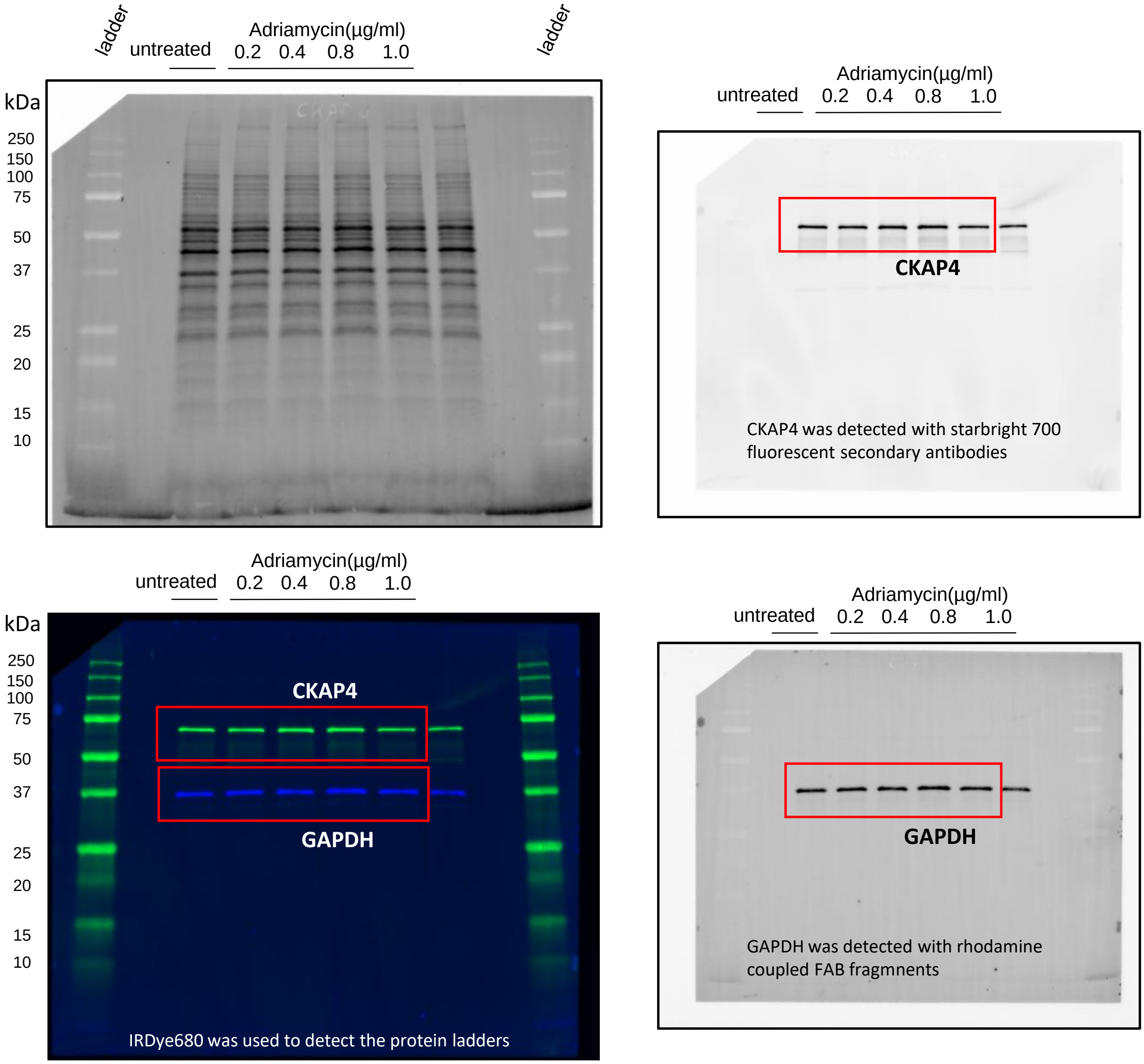

The same membrane used for CKAP4 and GAPDH was cut at 37 kDa and used for the detection of total CASP 3 (31 kDa)

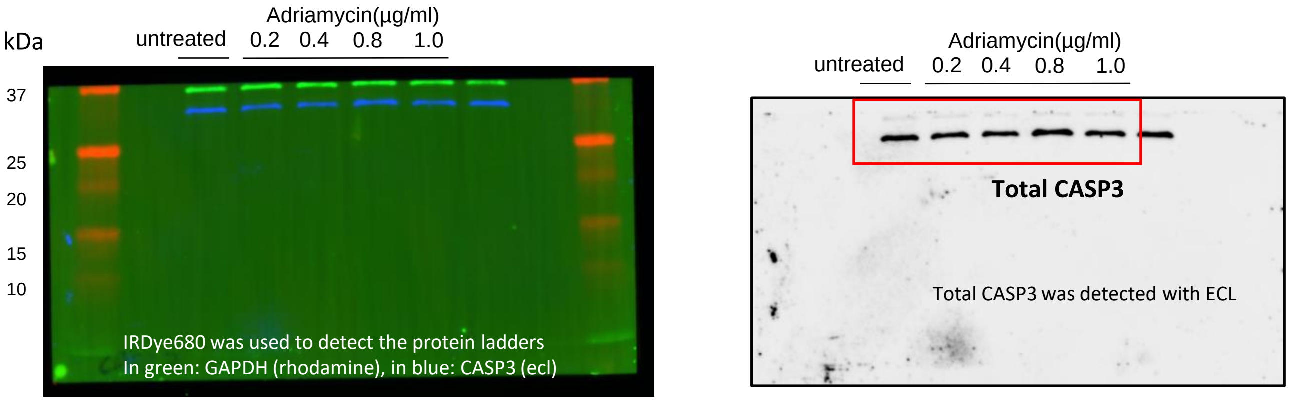

Full unedited blots for Figure 1F, part 2

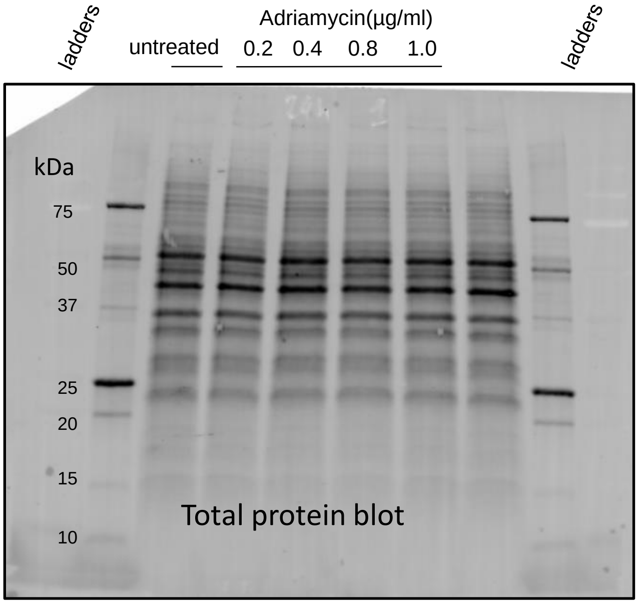

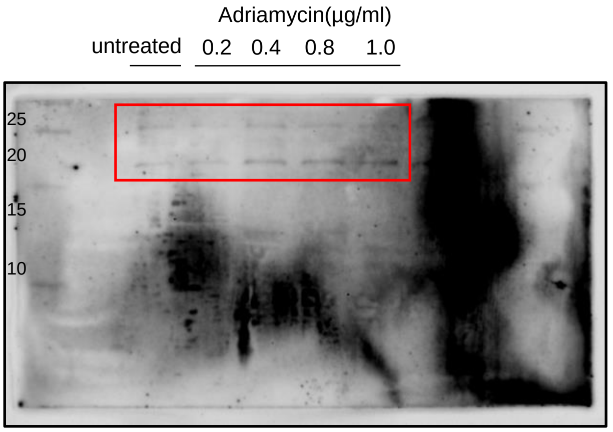

Cleaved CASP3

The membrane was cut at 25 Kda

Full unedited representative blots for Figure 3B

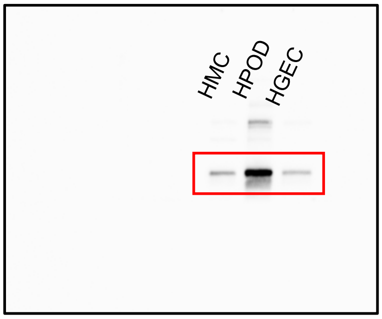

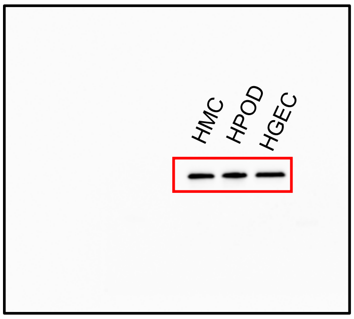

For Figure 3B, we have decided to use GAPDH as normalization factor instead of the total lane signal. Even when the lanes are equally loaded in terms of µg of total protein, protein content was broadly different when comparing the three cell types investigated (different proteomes).
Thus, we decide against using total lane normalization (only) in this case.

Full unedited blots for Figure 5B

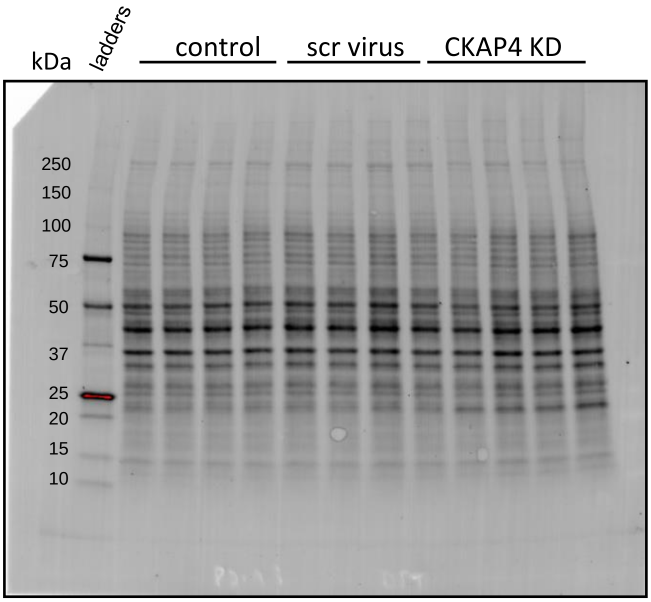

Total protein blot

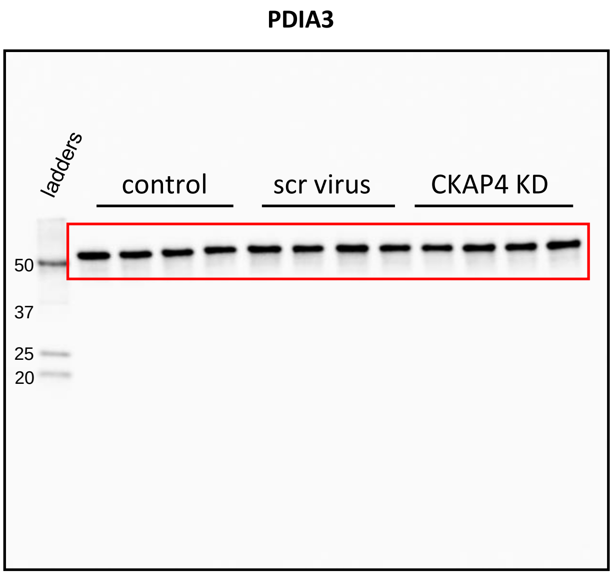

Full unedited blots for Figure 5C

ladders

kDa control scr virus CKAP4 KD

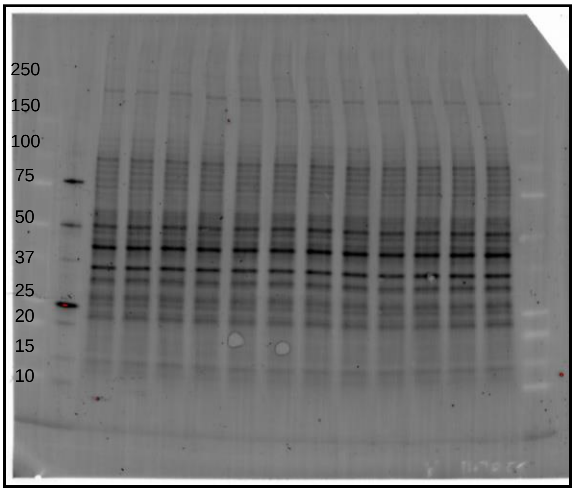

Total protein blot

ATF6α intact 100 kDa

kDa control scr virus CKAP4 KD

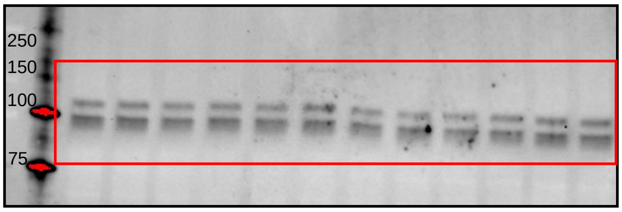

kDa ladders control scr virus CKAP4 KD

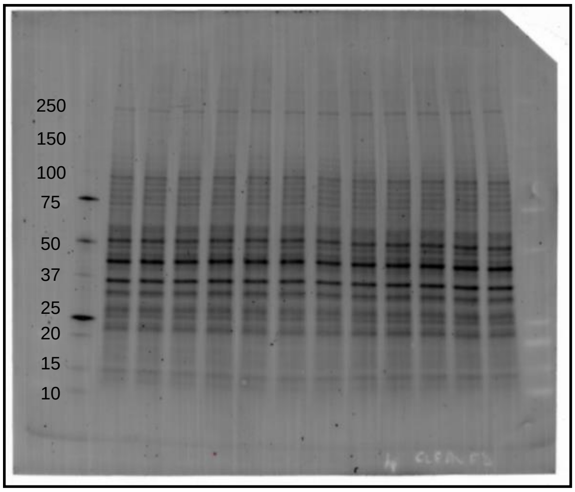

Total protein blot

ATF6α cleaved 36 kDa

control scr virus CKAP4 KD

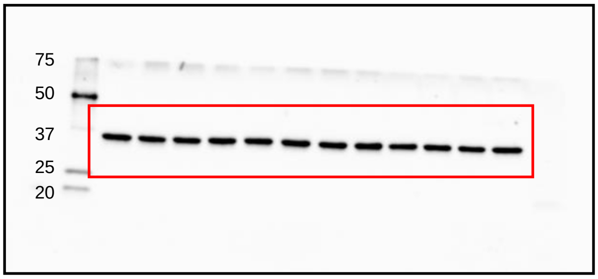

Full unedited blots for Figure 6A

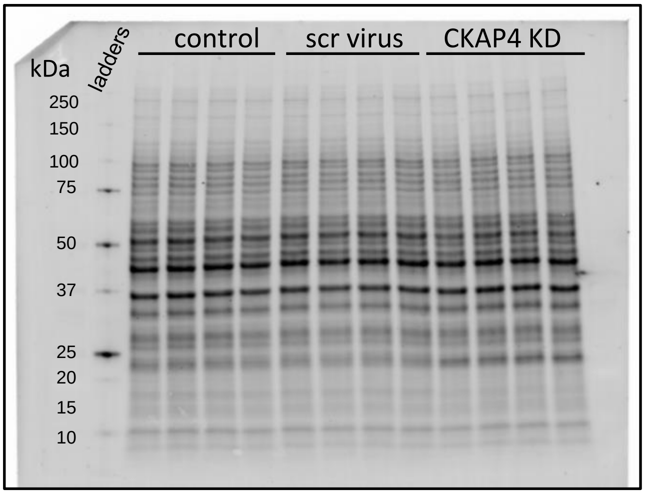

Total protein blot

ACTN4

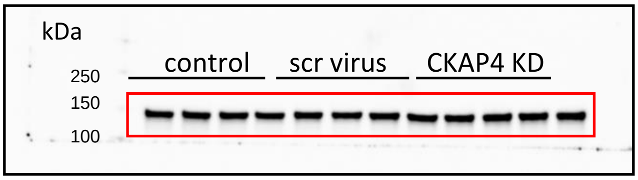

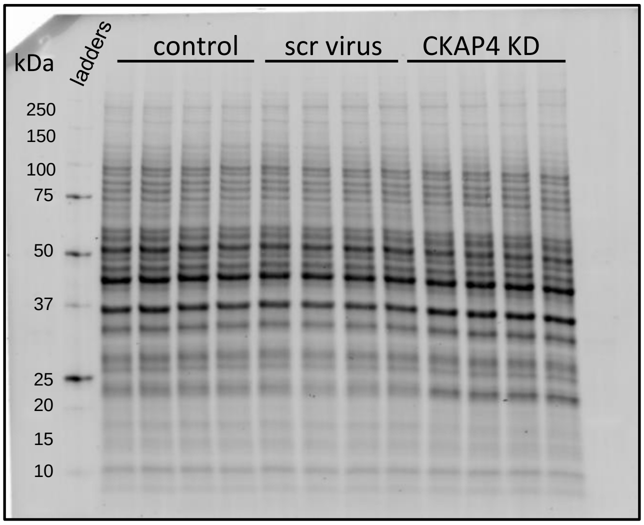

Total protein blot

TUBA - TUBB

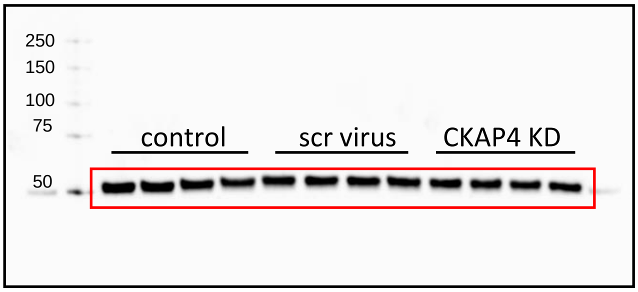

Full unedited blots for Figure 7D

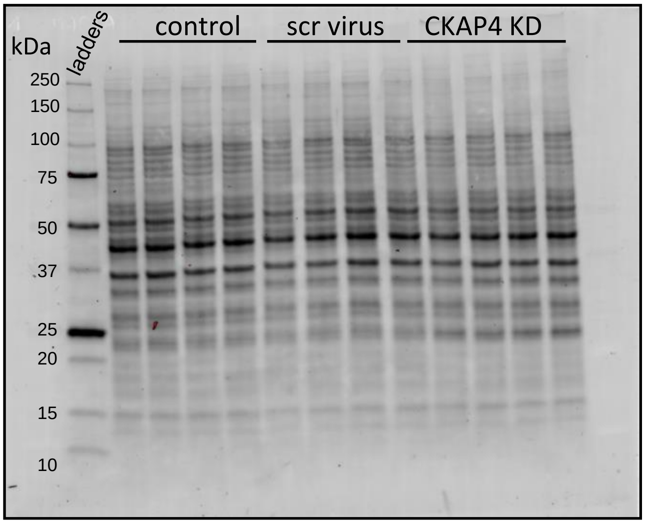

Total protein blot

MAP1A

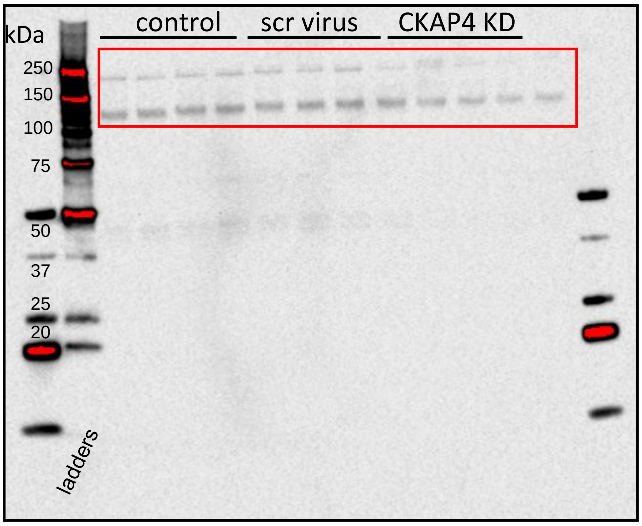

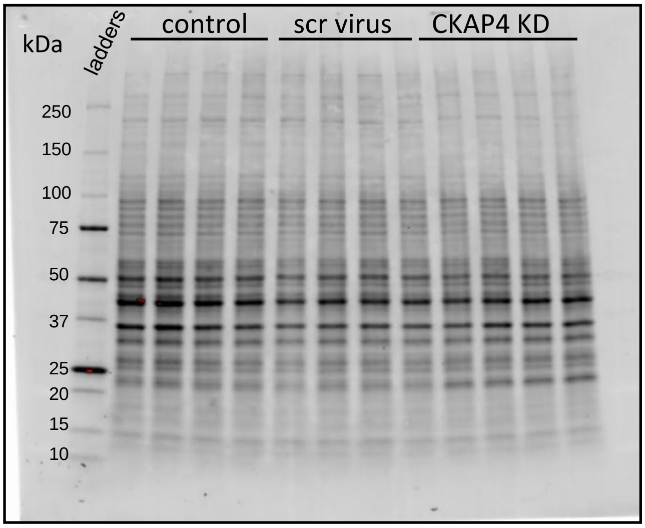

Total protein blot

DST

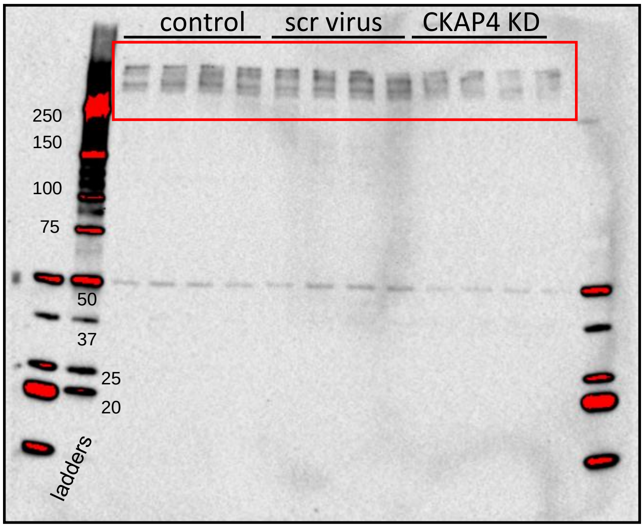

Full unedited blots for Figure 8B, part 1

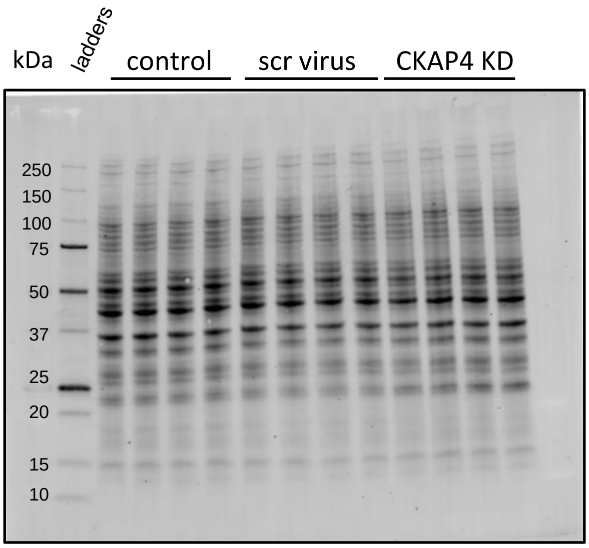

Total protein blot

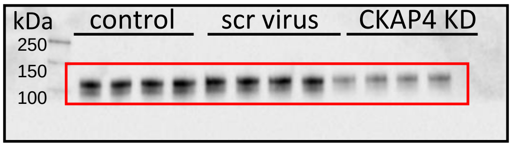

Total ITGB1

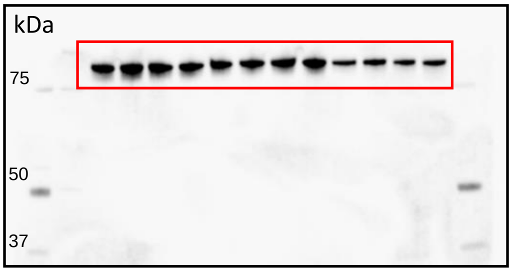

CKAP4

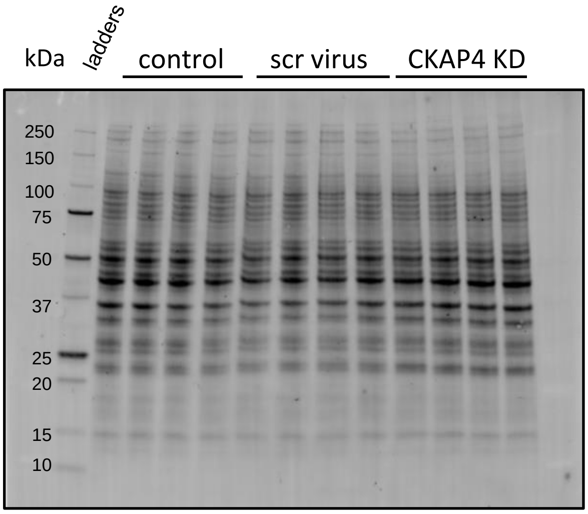

Total protein blot

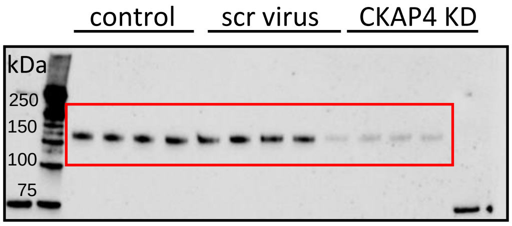

Active ITGB1

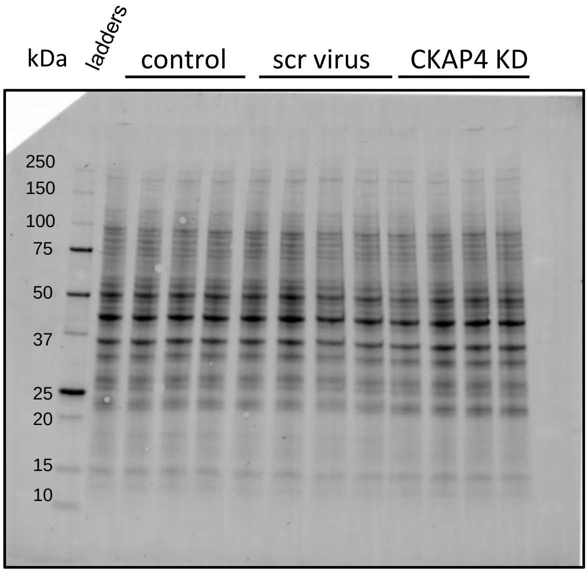

Total protein blot

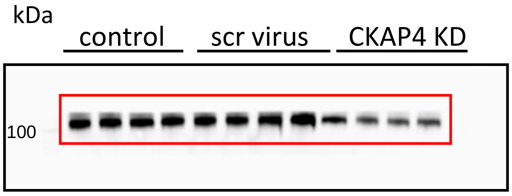

ITGB3

Full unedited blots for Figure 8B, part 2

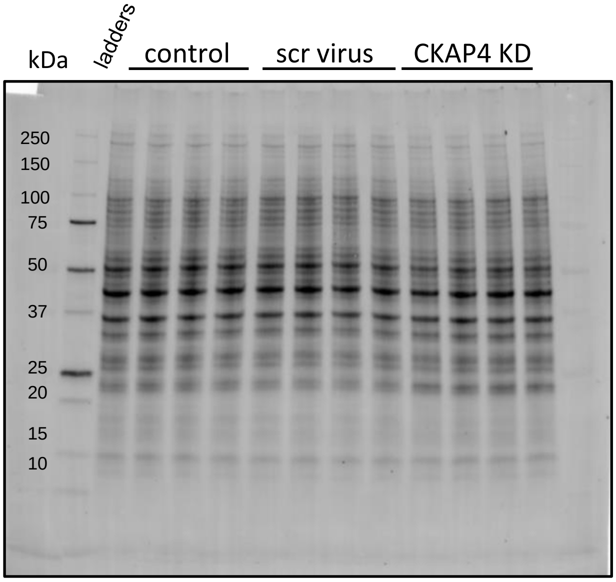

Total protein blot

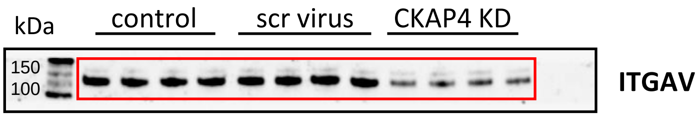

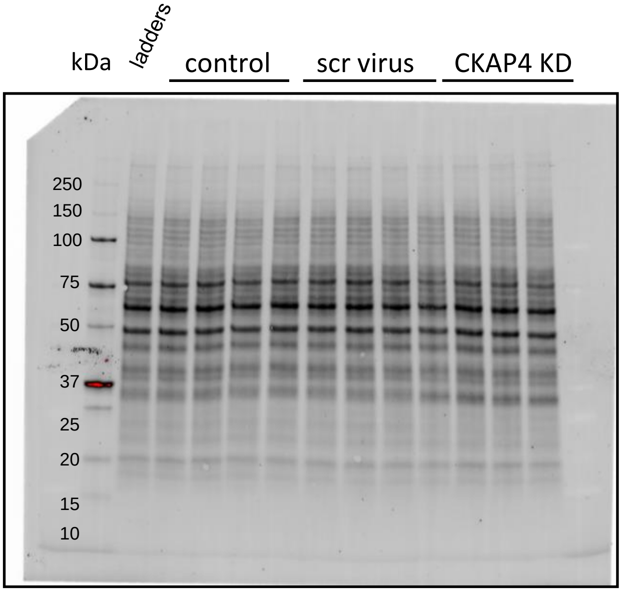

Total protein blot

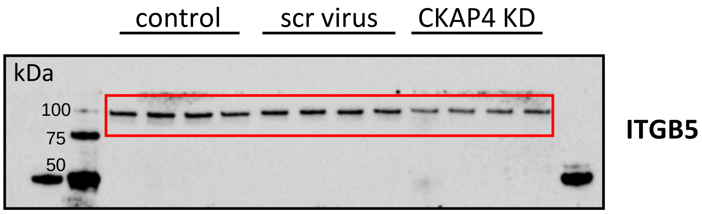

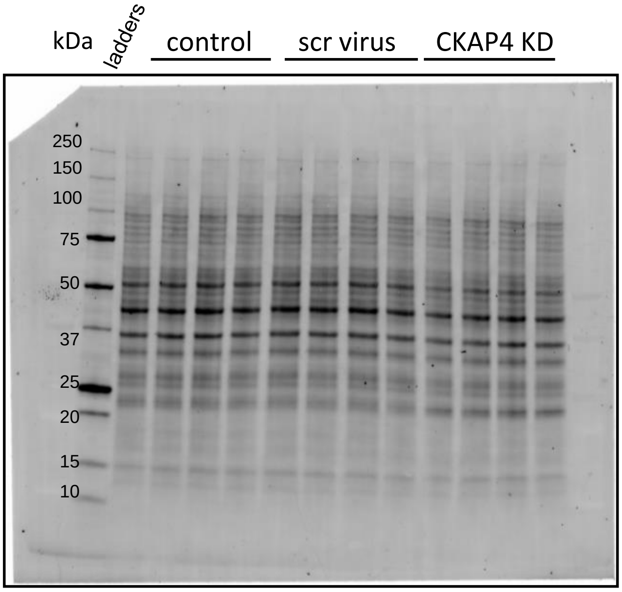

Total protein blot

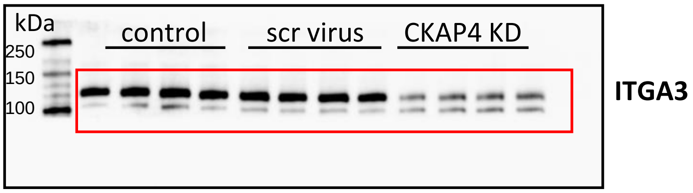

Full unedited blots for Figure 8B, part 3

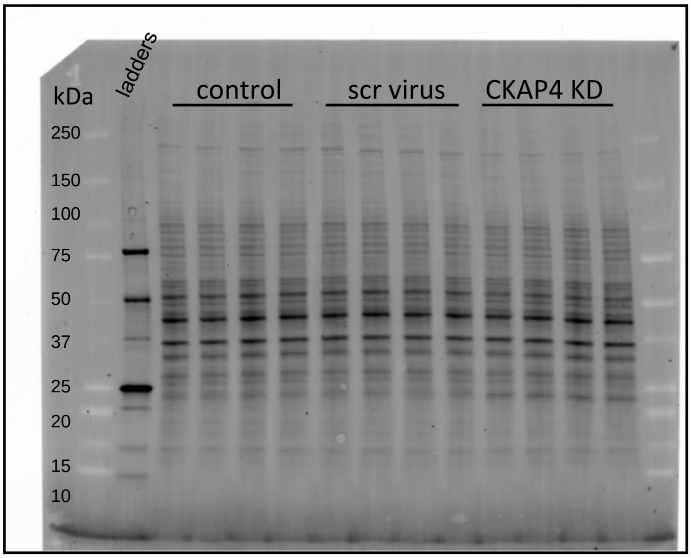

Total protein blot

TLN1

kDa

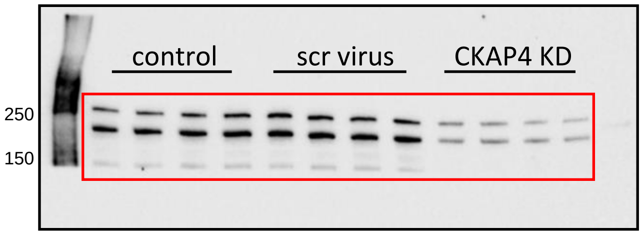

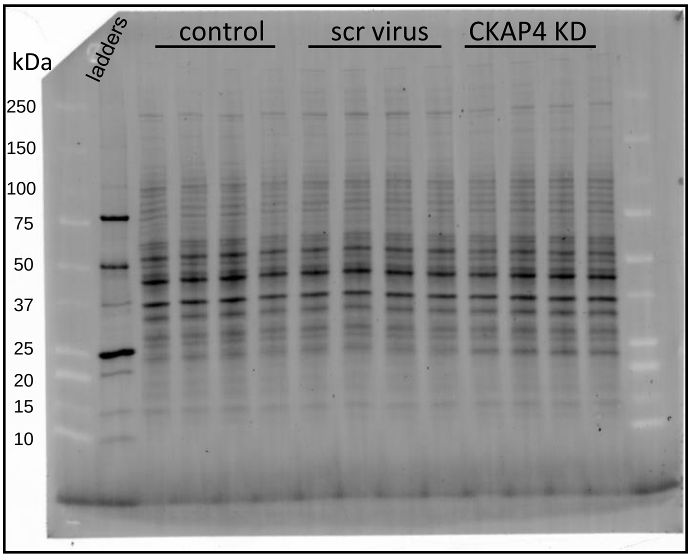

Total protein blot

RAP1A/1B

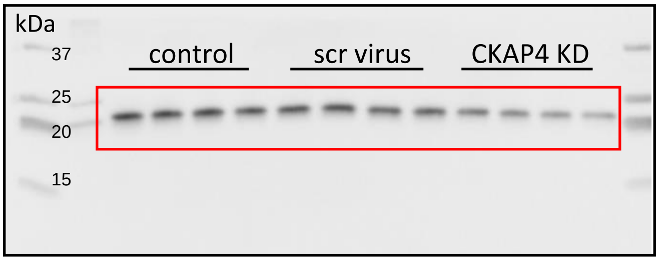

Full unedited blots for Figure 8D

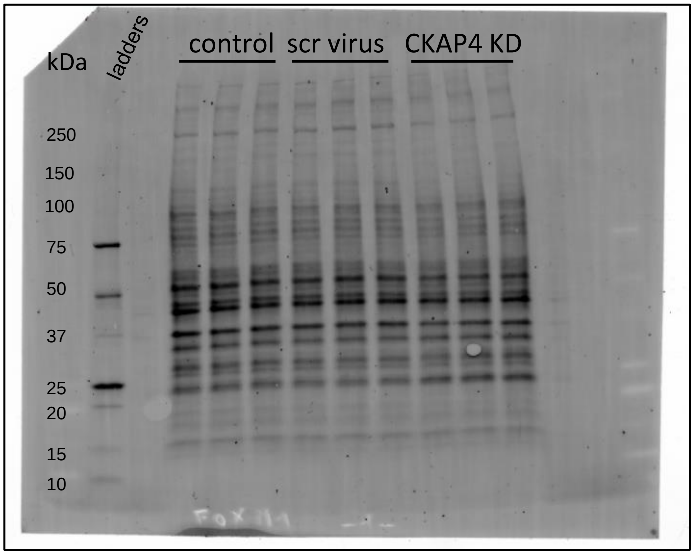

Total protein blot

FOXM1

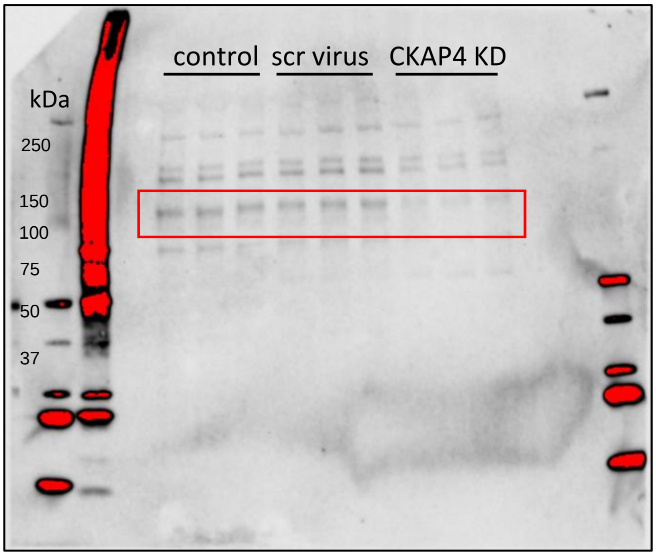

Full unedited blots for Supplemental Figure 1

A

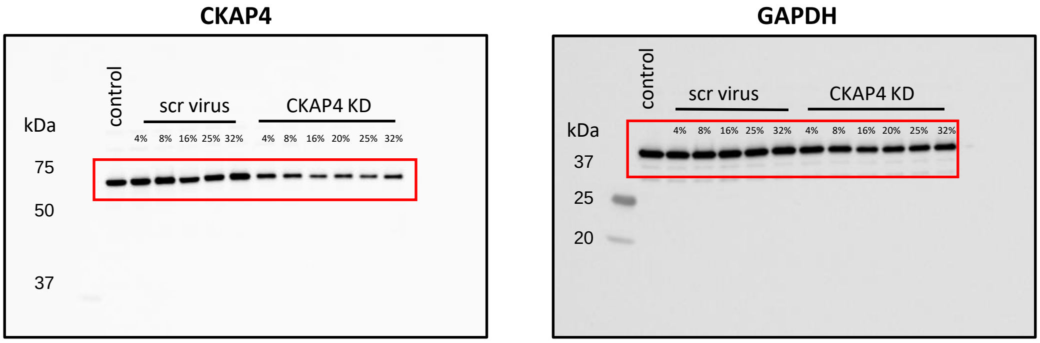

B

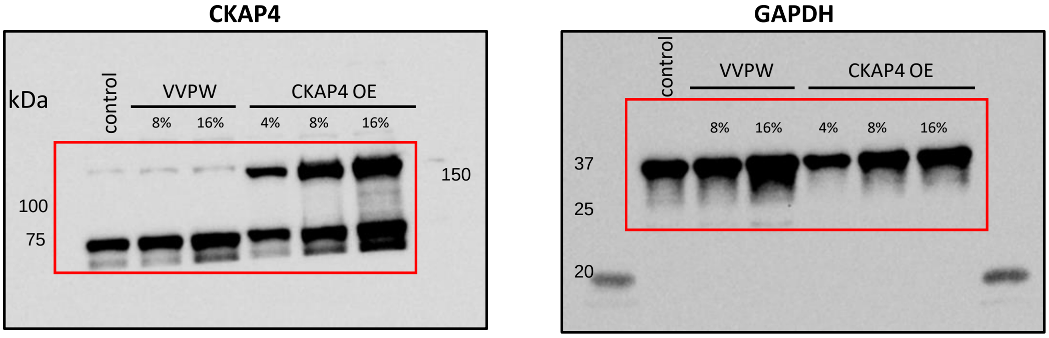

Supplement: Unedited blot and gel images [file jciinsight-10-181298-s181.pdf]
